# The impact of temporal hydrogen regulation on hydrogen exporters and their domestic energy transition

**DOI:** 10.1038/s41467-025-62873-w

**Published:** 2025-08-12

**Authors:** Leon Schumm, Hazem Abdel-Khalek, Tom Brown, Falko Ueckerdt, Michael Sterner, Maximilian Parzen, Davide Fioriti

**Affiliations:** 1 Research Center on Energy Transmission and Storage (FENES), Faculty of Electrical and Information Technology, University of Applied Sciences (OTH) Regensburg, Regensburg, Germany; 2 Department of Digital Transformation in Energy Systems, Technische Universität Berlin, Berlin, Germany; 3 Fraunhofer Research Institution for Energy Infrastructures and Geothermal Systems IEG, Cottbus, Germany; 4 Albert-Ludwigs Universität Freiburg, Faculty of Environment and Natural Resources, Freiburg im Breisgau, Germany; 5 Open Energy Transition, Bayreuth, Germany; 6 Potsdam Institute for Climate Impact Research, Potsdam, Germany; 7 University of Edinburgh, Institute for Energy Systems, Edinburgh, United Kingdom; 8 University of Pisa, Department of Energy Systems, Territory and Construction Engineering, Pisa, Italy

**Keywords:** Energy modelling, Renewable energy

## Abstract

As global demand for green hydrogen rises, potential hydrogen exporters move into the spotlight. While exports can bring countries revenue, large-scale on-grid hydrogen electrolysis for export can profoundly impact domestic energy prices and energy-related emissions. Our investigation explores the interplay of hydrogen exports, domestic energy transition and temporal hydrogen regulation, employing a sector-coupled energy model in Morocco. We find substantial co-benefits of domestic carbon dioxide mitigation and hydrogen exports, whereby exports can reduce market-based costs for domestic electricity consumers while mitigation reduces costs for hydrogen exporters. However, increasing hydrogen exports in a fossil-dominated system can substantially raise market-based costs for domestic electricity consumers, but surprisingly, temporal matching of hydrogen production can lower these costs by up to 31% with minimal impact on exporters. Here, we show that this policy instrument can steer the welfare (re-)distribution between hydrogen exporting firms, hydrogen importers, and domestic electricity consumers and hereby increases acceptance among actors.

## Abstract (web summary)

Although exporting hydrogen can raise domestic power costs, applying temporal matching with renewables can cut them by 31%, reduce emissions and steer welfare among exporters and consumers, offering a fairer approach to the global hydrogen trade.

## Introduction

The global energy environment is experiencing fundamental upheaval, driven by the need to reduce greenhouse gas emissions and transition to a low-carbon future^1,2^. In this context, hydrogen has emerged as a viable clean energy carrier capable of addressing the issues of decarbonizing numerous sectors such as industry, transport, heating and power generation^3–7^. A growing number of countries are investigating green hydrogen production and use as a crucial component of their strategy to cut greenhouse gas emissions and meet ambitious climate targets^3,8^. Simultaneously, several countries are positioning themselves as potential exporters of hydrogen and Power-to-X products–such as synthetic fuels or chemicals produced using renewable electricity–discovering an opportunity to leverage their renewable energy resources and technological advances^9^. These countries are expected to play a substantial role in the global energy market by supplying clean hydrogen and therefore contributing to global decarbonization efforts^9^. However, pursuing both (on-grid) hydrogen exports and national energy transition raises questions on welfare redistribution, prices and co-benefits that require in-depth analysis, additionally shining a light on the role of temporal hydrogen regulation.

**Fig. 1:**
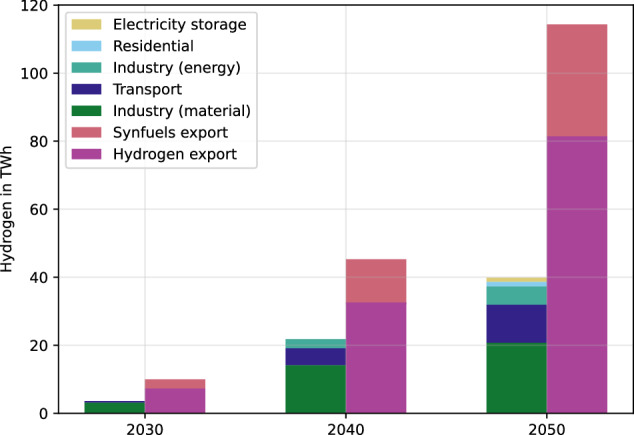
Hydrogen strategy of Morocco^10^. The export volume rises up to 115 TWh in 2050, the domestic demand up to 40 TWh in 2050. The largest share of domestic demand is in the industry sector. The exports are dominated by hydrogen, followed by synthetic fuels.

**Fig. 2:**
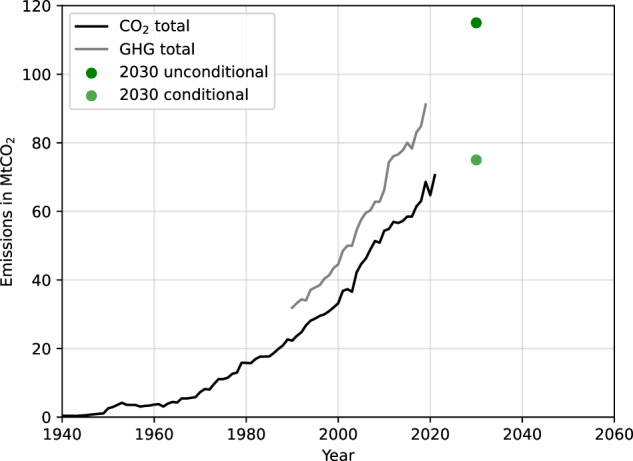
Morocco’s historical CO_2_ and greenhouse gas emissions (GHG). While emissions are rising, emission targets are in the range between 75 MtCO_2_e (conditional) and 115 MtCO_2_e (unconditional)^11^. GHG greenhouse gases.

Morocco serves as a blueprint for investigating these dependencies. It has implemented various strategies and policies to reach its climate targets and promote hydrogen exports. Figure 1 shows the hydrogen strategy of Morocco^10^, including national and export demands. By 2030, the total hydrogen demand adds up to 13.9 terrawatt-hours (TWh) and ramps up to 67.9 TWh in 2040 and 153.9 TWh in 2050, clearly listing higher demands for export than for domestic use. The hydrogen generation is backed by 5.2 gigawatts (GW) of renewable electricity in 2030, 23 GW in 2040, and 57.4 GW in 2050 in the export sector^10^. To cover the national demand of hydrogen, a Renewable Energy deployment of 1.6 GW in 2030, 7.0 GW in 2040 and 10.3 GW in 2050 is planned in Morocco’s Hydrogen Strategy^10^. In addition to the hydrogen strategy, Morocco has obliged to climate targets. Figure 2 displays the historical emissions and targets of Morocco. According to the self-defined national climate pledges under the Paris Agreement (NDC’s), Morocco aims to limit historically rising greenhouse gas emissions to 75 MtCO_2_e (conditional), respectively, 115 MtCO_2_e (unconditional) excluding emissions of Land Use, Land Use-Change and Forestry by 2030^11^. As stated by the Climate Action Tracker^11^, Morocco has not yet submitted a net-zero target.

Both the hydrogen export strategy and climate targets imply a substantial expansion of renewable electricity capacities. Furthermore, both strategies require an energy infrastructure development of e.g. electricity grid, hydrogen pipelines, CO_2_ network, ports and a considerable scale-up of electrolysers. Limited resources (e.g. land availability, renewable potentials, workforce, capital) require careful planning to minimize conflicts and maximize possible synergies. Even though Morocco has vast solar and wind resources^12–14^, it is a net importer of energy^15^, and green hydrogen could help reduce its dependency on energy imports. The proximity to Europe, which is expected to be a major hydrogen importer, makes Morocco an attractive potential exporter, opening the door to substantial economic opportunities and local value chains^16^.

These conditions are the motivation of several studies^17–25^ examining the potential of hydrogen in Africa and synergies with European demand. A more detailed study of Morocco has been undertaken by Boulakhbar et al.^26^ examining challenges in integrating Renewable Energies, Khouya et al.^27^ determining the Levelized Cost of Hydrogen based on concentrated solar power and wind farms, and Touili et al.^13^ investigating the potential of hydrogen from solar energy. Hampp et al.^28^ investigates various Power-to-X products and their transportation to Europe, and Eichhammer et al.^29^ highlights diverse opportunities and challenges related to exporting hydrogen and Power-to-X products from Morocco. Ishmam et al.^30^ assesses the impact of hydrogen exports on social factors and domestic jobs, going beyond a simplified energy potential analysis. While several studies^17,18,28^ have examined the potential of hydrogen as a low-carbon energy carrier and others have explored various countries’ climate targets and aspirations^26^, a substantial research gap remains regarding the integrated investigation of both perspectives. A recent study by Müller et al.^31^ contributes to this research gap by analysing energy transition pathways and green hydrogen exports in Algeria. They identify impacts on total system cost along four scenarios by applying an energy model with limited spatial (one node) and temporal (288 time slices per year) resolution. While this approach is a valuable contribution to this research gap, the study does not specifically outline synergies and conflicts for hydrogen exporters and domestic electricity consumers and differentiate between those interest groups. Due to their limited spatial and temporal resolution, this approach is unsuitable to identify transmission bottlenecks, infrastructure expansion requirements, and detailed system dynamics. However, their study design successfully combines the dimension of domestic energy transition and green hydrogen exports.

Apart from hydrogen exports and domestic carbon dioxide (CO_2_) mitigation, temporal hydrogen regulation plays a decisive role in prices for domestic consumers and hydrogen exporters. Temporal hydrogen regulation defines the rules for green hydrogen production when there is no direct connection between the electrolyser and green electricity production. Temporal matching in selected European countries is investigated in Zeyen et al.^32^, whereas Ruhnau et al.^33^ points out the effect of relaxing simultaneity requirements of renewable electricity supply and hydrogen generation on project design, economics, and power sector emissions. The effect of regulatory options on social welfare and carbon emissions is assessed in Brauer et al.^34^. They recommend that the regulation on the spatial dimension may not be too stringent, however they outline that the benefits of strict temporal matching (e.g. decline in emissions) exceed the moderate economic disadvantages. In addition, the need for such regulation diminishes in highly renewable electricity systems^34^. The interplay of additionality criteria and time matching requirements is investigated in Giovanniello et al.^35^, highlighting how additionality drives the emissions impact of temporal hydrogen regulation. While Zeyen et al.^32^ contextualizes the role of temporal hydrogen regulation with decarbonization scenarios of Germany and the Netherlands, the integration of domestic CO_2_ mitigation and temporal hydrogen regulation remains limited across the mentioned studies.

While most of the studies presented above focus exclusively on either hydrogen exports, domestic climate mitigation, or temporal hydrogen regulation, one study^31^ integrates two of these aspects. However, the complex relationship between all three dimensions remains only partially addressed in the existing literature. These studies miss interactions between on-grid hydrogen electrolysis and the domestic electricity system, fail to uncover potential synergies and conflicts for both hydrogen exporters as well as domestic electricity consumers with respect to different temporal hydrogen regulation regimes.

This study introduces a sector-coupled energy model (encompassing the residential, services, industry, transport and agriculture demand sectors) for the target region, the inclusion of modelling the temporal hydrogen regulation, and the evaluation of the synergies among hydrogen exports, domestic energy transition and hydrogen regulation. Additionally, we present a broad scenario vector, sweeping along the three scenario dimensions of: (i) Domestic CO_2_ mitigation: The domestic CO_2_ mitigation varies between 0–100% based on Morocco’s current emissions of 72 MtCO_2_e, (ii) Hydrogen export: The hydrogen export volume varies from 1–120 TWh, in accordance with Morocco’s hydrogen export ambitions of 114.7 TWh and (iii) Temporal hydrogen regulation: The temporal matching (of additional renewable electricity and the electrolyser electricity demand) varies between: no regulation, annual, monthly and hourly matching. The temporal matching regulation is implemented via a minimum constraint, stating that the renewable electricity generation is required to be equal or higher than the electricity demand for electrolytic hydrogen within every year (annual matching), month (monthly matching) or hour (hourly matching). We use the term hourly matching despite our three-hourly model because the principle of matching electricity demand for electrolysis and renewable electricity supply at each timestep is the same. This term effectively captures the mechanism, regardless of the model’s resolution. In the no regulation case, no such minimum constraint is applied. While our model is static, the scenario design allows exploration of different sequences in export and mitigation ambition.

This three-dimensional scenario space results in 264 model runs (excluding sensitivity analysis) to grasp the full extent of the interaction between various parameters. A 3-hourly resolution is chosen to capture energy system dynamics (e.g. renewable electricity generation profiles, energy storage operation) with its diurnal and seasonal variations in energy supply and demand. Similar studies^36,37^ find a minor underestimation of short-term battery storage and onshore wind and a minor overestimation of solar photovoltaics (PV) and hydrogen storage compared to an hourly resolution, overall justifying the reduction of the model size. A spatial resolution of 14 nodes represents the geographical heterogeneity of renewable electricity resources, demand centres and energy networks. Furthermore, the spatial resolution provides insights into land use conflicts and competing renewable electricity resources as well as a spatial differentiation of export ports. Since the 14 nodes align with the Global Administrative Areas level 1 regions^38^, policy advisement can be tailored to specific regions considering domestic needs and constraints.

Shortcomings of studies on highly renewable energy systems in Africa such as low temporal and spatial resolutions as well as the lack of sector-coupled energy models as pointed out in Oyewo et al.^39^ are tackled in this study. The analysis of Morocco’s energy system contributes to the expanding research that aims to inform policymaking and decision-making regarding sustainable energy transitions. This study provides insights into the pathways unlocking synergies and reducing conflicts between hydrogen exports and national energy transition, enabling Morocco and other potential exporting regions to follow a harmonious and sustainable trajectory while facing similar energy system planning challenges.

Here, we show the substantial co-benefits of domestic carbon dioxide mitigation and hydrogen exports, whereby exports can reduce market-based costs for domestic electricity consumers while mitigation reduces costs for hydrogen exporters. Under various hydrogen export volumes, climate targets and temporal matchings, we demonstrate that temporal hydrogen regulation can lower the market-based costs for domestic electricity consumers by up to 31% with minimal impact on exporters. This study presents a fully sector-coupled capacity expansion and dispatch model of Morocco, which includes both gas pipelines and electricity networks. We use costs referring to model inputs and system costs, while prices denote endogenous shadow prices (representing idealized market prices) derived from the model. Payments made by consumers (calculated as energy price times energy volume) are described as market-based (consumer) costs based on idealized market prices or, alternatively, (consumer) payments to highlight their financial impact. These payments only translate into one component of consumer electricity bills if whole-sale electricity prices are passed through to the retail tariff. Additional components in retail electricity prices depend on region-specific regulation and are not included here.

## Results

### Impacts of domestic CO_2_ mitigation on the electricity system

**Fig. 3:**
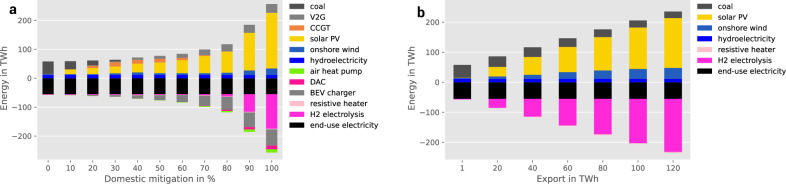
Electricity supply and demand at fixed export levels and increasing domestic CO_2_ mitigation and vice versa with hourly temporal hydrogen regulation. Panel **a** shows the electricity supply and demand at fixed export (1 TWh) and 0–100% domestic CO_2_ mitigation. Increasing domestic CO_2_ mitigation first phases out carbon-intensive coal generation in favour of combined-cycle gas turbines (CCGT), at medium to high domestic CO_2_ mitigation the electricity system is fully renewable supported by flexibility through Vehicle-to-Grid (V2G) and sector coupling. Increasing electricity demands include Battery Electric Vehicles (BEV) and hydrogen (H_2_) generation for other sectors. Panel **b** shows the electricity supply and demand at fixed domestic CO_2_ mitigation (0%) and 1–120 TWh export. At increasing hydrogen exports the additional electricity required for hydrogen electrolysis is covered by onshore wind and solar photovoltaics (PV), as imposed by the temporal hydrogen regulation. See Supplementary Fig. 4 for electricity supply and demand if no hydrogen regulation is in place and Supplementary Fig. 9 for hydrogen exports up to 200 TWh. DAC direct air capture.

First of all, we investigate the supply and demand of Morocco’s electricity system depending on domestic CO_2_ mitigation (scenario dimension 1). Figure 3 depicts the supply and demand of the electricity system at increasing climate mitigation ambitions from 0% to 100%. At low climate ambitions, the electricity demand is mainly covered by coal power, existing (brownfield) capacities of onshore wind, hydro, and combined-cycle gas turbines (CCGT) play only a minor role. At increasing domestic CO_2_ mitigation, coal power is phased out in favour of solar PV, furthermore a fuel switch from coal to gas (CCGT) is observable at increasing emission reductions. At medium climate mitigation ambitions, the dipatchable power in the electricity system is provided by CCGT. Further increasing the climate ambitions, wind onshore and especially solar PV penetrate and dominate the electricity system complemented by dispatchable power from Vehicle-to-Grid providing an almost fully renewable electricity sector at 100% emission reduction. While mitigation targets can (in part) be met with technologies like fossil gas with CCS or Direct Air Capture, the model finds that increasing renewable capacities, particularly solar PV and wind, is the most cost-effective solution (Fig. 3). As mitigation efforts intensify, renewables become the cost-optimal choice for decarbonizing the energy system due to their cost-competitiveness and large-scale availability.

At 70% and above, the electricity demand of eletrolysers increases substantially to supply hydrogen allowing a switch from fossil oil products to Fischer-Tropsch fuels in various sectors (see Supplementary Fig. 18). The Fischer-Tropsch demand in the transport sector increases, even though the increasing Battery Electric Vehicle (BEV) diffusion is counterbalancing the demand for oil products (see Supplementary Fig. 2). Both electrolysers (see Supplementary Fig. 16) and BEVs drive the electricity demand substantially, up to two times of the domestic end-use electricity demand. Additionally, the electricity demand for air heat pumps increases, supporting the defossilisation in the heating sector and supplying heat for Direct Air Capture required for synthetic fuels.

### Impacts of hydrogen exports on the electricity system

Here, we analyse the hydrogen export ramp up (scenario dimension 2). The electricity supply at increasing hydrogen exports is displayed in Fig. 3. The deployment of hydrogen exports requires a scale up of onshore wind and solar PV accordingly, as defined by the green hydrogen constraint outlined in Green hydrogen policy. Without the temporal hydrogen regulation, the additional electricity demand of hydrogen exports is covered by coal power plants and CCGT (by increasing the capacity factor of existing brownfield coal and CCGT capacities) and an expansion of open-cycle gas turbines (OCGT) installation and supply as pointed out in Supplementary Fig. 4. The cost-optimal solution of the model shows, that increasing hydrogen exports do not necessarily require additional renewable capacities. Instead, the electricity demand for hydrogen electrolysis is covered by additional electricity supply from fossil generation. However, if the cost-optimal solution is constrained by the green hydrogen policy, an increase of hydrogen exports goes along with additional renewable electricity capacities as defined in the EU’s delegated Act. In contrast to the exponential increase of electricity demand observable at increasing climate mitigation ambitions displayed in Fig. 3, the electricity demand increases linearly with the hydrogen export ambitions.

### Co-benefits of domestic CO_2_ mitigation and hydrogen exports

In an integrated analysis of domestic CO_2_ mitigation (scenario dimension 1) and hydrogen exports (scenario dimension 2) this study outlines that both domestic CO_2_ mitigation and hydrogen exports profit from each other and show co-benefits. In this section, we i) show the effects of hydrogen exports on market-based costs for domestic electricity consumers, ii) the role of domestic CO_2_ mitigation on hydrogen export cost, and lastly iii) common co-benefits.

**Fig. 4:**
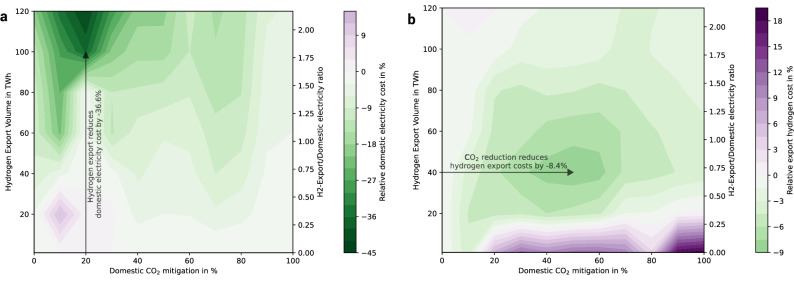
The effects of hydrogen exports on domestic electricity consumers and domestic CO_2_ mitigation on hydrogen exporters. Panel **a** shows the effect of hydrogen (H_2_) exports on domestic electricity cost with hourly hydrogen regulation. Therefore, the effect of hydrogen exports is isolated by normalizing the costs to 1 TWh hydrogen export in each column. This approach allows for a vertical interpretation in Panel **a** only, displaying the effects of hydrogen exports (vertical) at a certain domestic CO_2_ mitigation level. Panel **b** shows the effect of domestic CO_2_ mitigation on hydrogen export cost with hourly hydrogen regulation. Therefore, the effect of domestic CO_2_ mitigation is isolated by normalizing the costs to 0% CO_2_ mitigation in each row. This approach allows for a horizontal interpretation in Figure Panel **b** only, displaying the effects of domestic CO_2_ mitigation (horizontal) at a certain hydrogen export quantity. Domestic electricity consumers profit from increasing hydrogen exports, especially at low domestic CO_2_ mitigation and high exports. Hydrogen exporters profit from domestic CO_2_ mitigation at medium mitigation efforts. See Supplementary Fig. 17 for absolute market-based costs for domestic electricity consumers and hydrogen exporters, Supplementary Fig. 6 for the interplay of hydrogen exports and domestic CO_2_ mitigation if no hydrogen regulation is in place and Supplementary Fig. 10 for hydrogen exports up to 200 TWh.

First, Fig. 4 Panel a illustrates how domestic electricity consumers benefit from a stronger hydrogen export industry at different decarbonization targets. The costs are normalized to the cost level at 1 TWh hydrogen export for each domestic CO_2_ mitigation level, showing the relative impact of hydrogen exports on domestic electricity costs. Negative values indicate that hydrogen exports reduce average market-based electricity costs for domestic consumers. Figure 4 shows, that hydrogen exports decrease the relative domestic electricity costs by up to 45%, especially at mitigation levels below 40% and exports above 50 TWh. In these ranges, the domestic electricity consumers profit from excess green electricity originating from additional renewable energy capacities required for hydrogen export. These renewable capacities i.) displace fossil generation with high short-run marginal costs (price spillover) and ii.) provide excess electricity to the domestic electricity system (energy spillover), elaborated in detail in the section on the Impacts of hydrogen regulation on electricity prices and in the Supplementary Figs. 12–14. However, as the domestic electricity system undergoes deeper decarbonization (domestic CO_2_ mitigation above 50–60%), the cost-reducing effect of hydrogen exports weakens. Here, the impact of additional renewable electricity capacities is only marginal, since the electricity system is already highly or fully renewable electricity based and neither price spillover nor energy spillover are effective as outlined in the section Discussion. The only scenario in which an increase of hydrogen export leads to higher domestic electricity costs is at a low CO_2_ mitigation level of 10% and exports of 20 TWh. This effect arises due to the initial increase in electricity demand from hydrogen electrolysis, which raises electricity cost, as seen in Supplementary Fig. 6 (without temporal regulation). Without regulation, this cost increase continues with higher exports. However, with regulation, the cost-reducing effect of hydrogen exports eventually dominates through the effect of energy and price spillovers, as discussed in the section on Impacts of hydrogen regulation on electricity prices, mitigating further cost increases and significantly decreasing the market-based costs for domestic electricity consumers.

Second, Fig. 4 Panel b shows how hydrogen exporters benefit from domestic CO_2_ mitigation. The costs are normalized to the cost level at 0% CO_2_ mitigation for each hydrogen export volume, displaying the relative impact of domestic CO_2_ mitigation on hydrogen export costs. At hydrogen export volumes of 25–75 TWh, increasing domestic CO_2_ mitigation up to 40–60% reduces hydrogen export costs by up to 9%. This cost reduction is driven by improvements in system flexibility–such as BEV charging, energy storage, and expanded transmission capacities (Fig. 3)–which also benefit hydrogen exporters. However, at even higher domestic CO_2_ mitigation levels (70–100%), further renewable expansion is required, tapping into sites with less favourable weather conditions in Morocco. This ultimately raises electricity costs, as seen in Supplementary Fig. 7d. At higher hydrogen export volumes, this effect diminishes. As the hydrogen export system grows significantly larger than the domestic electricity system, shared infrastructure effects become less relevant as shown in Fig. 4 and in the sensitivity analysis for higher exports in Supplementary Fig. 10b. The only scenario where domestic CO_2_ mitigation leads to a notable increase in hydrogen export costs occurs at low export volumes below 20 TWh. Here, advances in domestic CO_2_ mitigation increase hydrogen export costs by up to 19% compared to a scenario without domestic CO_2_ mitigation. This cost increase results from lower electrolysis capacity factors compared to the 0% CO_2_ mitigation scenario, where we observe high electrolysis capacity factors of above 80% (see Supplementary Fig. 7b). The top-left region of Fig. 4 Panel b indicates that at certain export levels, hydrogen export costs become largely independent of domestic CO_2_ mitigation. This is driven by temporal hydrogen regulation further investigated in the section on Impacts of hydrogen regulation on electricity prices. Here, hydrogen export infrastructure decouples from the domestic electricity system, the cost is increasingly independent of domestic CO_2_ mitigation. When hydrogen exports reach twice the size of domestic electricity demand (Fig. 4), the influence of the domestic electricity system on hydrogen export costs becomes negligible.

Third, we derive co-benefits for domestic CO_2_ mitigation and hydrogen exports. Within the area of 40–60% CO_2_ mitigation and 50–100 TWh hydrogen exports, i) domestic electricity consumers profit from hydrogen exports compared to very low (1 TWh) exports and ii) hydrogen exporters decrease their cost compared to no (0%) domestic CO_2_ mitigation. Hence, both domestic CO_2_ mitigation and hydrogen exports have cost-reducing effects on hydrogen exporters and domestic electricity consumers respectively. They benefit from each other (co-benefit) at medium mitigation (40–60%) and moderate to high hydrogen exports (50–100 TWh).

### Impacts of hydrogen regulation on electricity prices

Hydrogen regulation has strong effects on electricity system dispatch (and emissions), increasing the prices and consumer cost for hydrogen exporters whilst decreasing for domestic electricity consumers. This section shows the mechanisms of temporal matching in the 120 TWh export and 0% domestic CO_2_ mitigation scenario.

As the temporal matching becomes stricter, additional renewable capacities and complementing hydrogen storage are required to meet the electricity demand of electrolysis on a time base defined by the temporal matching constraint, as shown in Supplementary Fig. 5. These renewable capacities i.) push out fossil generation with high short-run marginal costs (price spillover) and ii.) provide excess electricity to the domestic electricity system (energy spillover). Both spillover effects are linked, the energy spillover causes a merit order effect affecting the domestic electricity prices.

**Fig. 5:**
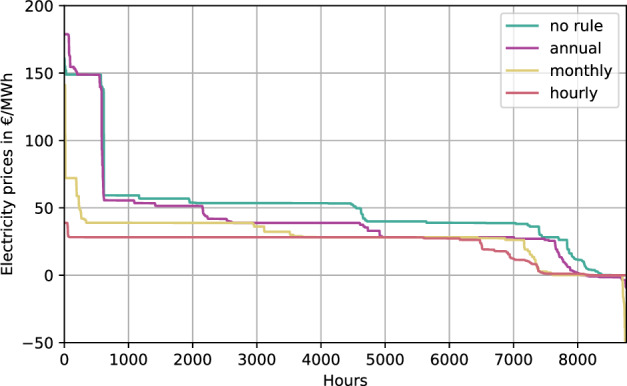
The effect of temporal hydrogen regulation on electricity prices. Figure shows the price duration curve at 120 TWh export and 0% domestic CO_2_ mitigation. Stricter temporal hydrogen regulation pushes the price duration curve towards the left, as additional renewable electricity capacities phase out fossil generation with higher short-term marginal costs than renewables. Negative prices below −50 € per megawatt-hour (€ MWh^−1^) are cut off.

The price spillover is observable in Fig. 5 depicting the price duration curve at 120 TWh export and 0% domestic CO_2_ mitigation. The price duration curve describes the sorted price of electricity (spatial mean) at all modelled hours of the year. Stricter hydrogen regulation pushes the price duration curve towards the left, as additional renewable electricity capacities phase out fossil generation, which has higher short-run marginal costs due to fuel costs, compared to renewable electricity. As renewables generate power when available, they reduce the dispatch of fossil generators, whose higher marginal costs make them less competitive in the short term. However, fossil fuels still play a complementary role, stepping in to fill gaps during periods of low renewable generation. This mechanism is fully captured in our capacity expansion and operation model, which accounts for both types of generation in response to price changes and regulatory shifts.

**Fig. 6:**
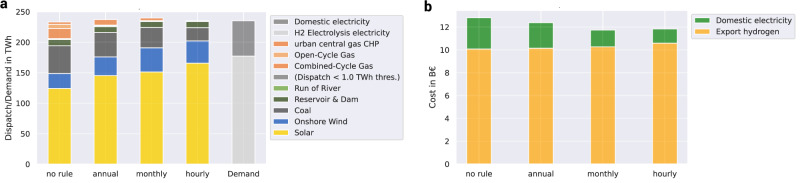
The effect of temporal hydrogen regulation on the electricity system and consumer costs. Panel **a** shows the electricity dispatch and demand and Panel **b** shows the cost for consumers for various (hydrogen) temporal matching regimes in the 120 TWh export and 0% CO_2_ mitigation scenario. Stricter temporal matching decreases carbon-intensive electricity generation (coal & gas) for hydrogen generation and even domestic electricity consumers (Panel **a**). Cost for export hydrogen generation increase to fulfil the temporal matching constraint, whereas domestic electricity consumers profit from stricter hydrogen regulation (Panel **b**). CHP combined heat and power.

The energy spillover is shown in Fig. 6. In the no rule scenario, which does not constrain the electricity input of hydrogen electrolysis, the electricity supply of coal and OCGT is substantially higher than in the annual, monthly, or hourly case. If annual matching is applied, the renewable electricity supply to the electricity system equals the annual demand for electrolysis, increasing the supply of solar PV by 20 TWh while decreasing the supply of fossil electricity generation. Monthly matching increases this trend, while hourly matching completely phases out OCGT and CCGT. Figure 6 highlights the energy spillover effect: going beyond the electricity demand of hydrogen electrolysis, renewable capacities installations necessary to meet the temporal matching decarbonize the domestic electricity demand and decrease the grid emissions. This effect is steered by the strictness of temporal matching, hence stricter temporal matching rules enable a further decrease in emissions. The slight increase in total dispatch for annual and monthly matching is balanced by resistive heaters in the heating sector. In addition to the synergies between hydrogen export and domestic CO_2_ mitigation, the energy spillover outlines a clear synergy between domestic CO_2_ mitigation and temporal hydrogen regulation.

The price spillover effect results in lower electricity prices for domestic consumers. Taking the electricity demand into account, the decrease of prices results in a decrease of electricity cost shown in Fig. 6. Stricter temporal hydrogen regulation decreases the domestic electricity cost from 2.7 billion € (bn€) to 1.2 bn€. In contrast, the hydrogen cost for exporters increases from 10.1 bn€ to 10.6 bn€, carrying the cost of the temporal matching constraint and hence financing additional solar PV and hydrogen storage required to meet the electrolysis electricity demand on a certain temporal basis. See Supplementary Figs. 7,  8 for cost breakdown of hydrogen electrolysis with and without hourly temporal matching.

The combined cost borne by domestic electricity consumers and hydrogen exporters decreases with stricter temporal hydrogen regulation, while the overall system cost increases, as shown in Supplementary Fig. 5. This difference arises because electricity consumers (both domestic and hydrogen electrolysis) pay a contribution margin to the capital costs of existing coal power plants, which are pre-existing brownfield assets. Since these brownfield capacities are not part of the objective function, their capital costs are excluded from the total system cost (see Supplementary Fig. 5) but are included in consumer costs (see Fig. 6). Under stricter temporal hydrogen regulation, coal power phases out, reducing the capital contributions required from consumers for these coal assets. For domestic electricity consumers, this leads to a direct decrease in their cost. However, for hydrogen exporters, the cost slightly increases, as they bear the cost for the additional renewable capacities (see Supplementary Fig. 5) required to meet temporal hydrogen regulation. As coal capacity declines with stricter hydrogen regulation, the discrepancy between consumer costs and the total system cost decreases. This observation also holds if the coal capacity is decreased under high decarbonization scenarios where fossil fuel dispatch is minimized.

To sum up, temporal hydrogen regulation is a policy instrument acting in a twofold way. It steers the redistribution of costs between hydrogen exports and domestic electricity consumers (based on the price spillover effect), decreasing the cost for domestic electricity consumers and increasing the cost for hydrogen exporters. Second, the temporal hydrogen regulation not only decarbonizes hydrogen exports, but additionally decreases grid emissions of the domestic electricity demand via the energy spillover effect. Both effects scale with stricter temporal matching.

### Role of hydrogen regulation in mitigation-export contexts

Based on the effects of temporal hydrogen regulation in the scenario with 120 TWh exports and 0% CO_2_ mitigation presented in the previous section, we now broaden the analysis to the full scenario space of mitigation (0–100%) and export levels (0–120 TWh). Although the model is static and optimized for 2030, we interpret the scenario variation as reflecting different sequences of policy ambition – that is, whether hydrogen exports or domestic decarbonization are prioritized earlier on the pathway toward shared long-term goals. For instance, a scenario with high exports and low CO_2_ mitigation can reflect a pathway where export infrastructure is deployed early, with decarbonization following later and vice versa. To explore this sequencing, we group scenarios into three illustrative transition strategies: (i) Quick exports and slow CO_2_ mitigation, (ii) Balanced exports and CO_2_ mitigation and (iii) Slow exports and quick CO_2_ mitigation as further elaborated in Supplementary Fig. 3a. These groupings help illustrate the impacts of temporal hydrogen regulation on e.g. domestic electricity cost, even within a static modelling framework.

**Fig. 7:**
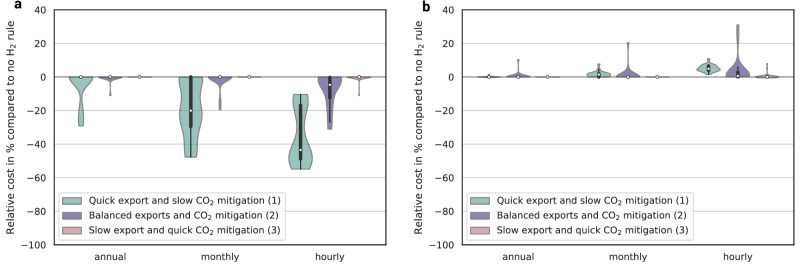
Relative change of electricity and hydrogen consumer cost depending on the temporal hydrogen regulation. Violin plots indicate median (white dot), interquartile range (thick black bar), and 1.5× interquartile range (thin black bar), using kernel density estimation to show the distribution shape of the mitigation-export scenarios, grouped into three stylized transition strategies (1–3) reflecting different sequences of export and mitigation ambition. Domestic electricity consumers (Panel **a**) profit across all export and mitigation scenarios but most in the group of slow export and quick CO_2_ mitigation scenarios. Hydrogen exporters (Panel **b**) experience higher cost with stricter temporal hydrogen regulation. The temporal hydrogen (H_2_) regulation regulates the welfare distribution between both groups. See Supplementary Fig. 11 for the sensitivity of the export quantities and Supplementary Fig. 3 for scenario grouping.

Figure 7 shows the relative change of electricity and hydrogen cost of all mitigation-export scenarios (grouped by transition strategy) in dependence of temporal matching. The effects of temporal hydrogen regulation on market-based costs for domestic electricity consumers are most dominant in the group of Quick exports and slow CO_2_ mitigation. In this group, the introduction of annual matching has already a striking effect, because the price spillover observed in Fig. 5 decreases (domestic) electricity prices. The price spillover unfolds in fossil-dominated (hence low domestic CO_2_ mitigation) systems, combined with high hydrogen exports providing a strong energy spillover effect. In the cases of high exports and no domestic CO_2_ mitigation, the cost of electricity for domestic consumers reduces up to 55% in the case of hourly matching. This effect is even more pronounced at even higher export quantities, as observable in the sensitivity analysis carried out in Supplementary Fig. 11.

In the group of Balanced exports and CO_2_ mitigation the effect of temporal hydrogen regulation is less dominant. Hourly matching reduces the market-based costs for domestic electricity consumers by up to 31% (Fig. 7). In only two single mitigation-export scenarios the annual or monthly matching decreases the cost for domestic electricity, whereas all other combinations of the Balanced exports and CO_2_ mitigation group are only sensitive to the strictest – hourly matching – hydrogen regulation. The cost increase for hydrogen exporters (Fig. 7) is minimal, the median of the Balanced exports and CO_2_ mitigation scenarios indicates a 1% increase. In only two scenarios, hourly matching regulation increases the hydrogen cost above 7% (above 11% across all groups) compared to no temporal hydrogen regulation. This is the case for 0% CO_2_ mitigation and 1 and 20 TWh export scenarios.

In the Slow exports and quick CO_2_ mitigation group, only the hourly matching has observable effects, since the electricity system is already dominated by renewable electricity and only hourly matching phases out the remaining fossil generation. Therefore, the impacts on the market-based costs for domestic electricity consumers (up to 11%) and hydrogen exporters (up to 8%) are moderate. Figure 7 shows, that temporal hydrogen regulation hedges domestic electricity consumers against rising costs across all scenario groups. Setting aside the less likely scenarios of the Quick exports and slow CO_2_ mitigation group (based on the assumption that export quantities above 80 TWh without domestic CO_2_ mitigation are unlikely), hourly matching decreases the cost of electricity for domestic consumers by up to 31%. The cost for hydrogen exporters increase only slightly in the vast majority of all mitigation-export scenarios.

## Discussion

The transition towards climate neutrality of Morocco’s energy system and the ramping up of exports up to 120 TWh are a matter of decade(s) and undergo a certain pathway. However, the broad scenario design of this study allows conclusions and recommendations beyond the specific Morocco case. Various potential hydrogen exporting countries with different prerequisites can be found in the integrated analysis of benefits and burdens for domestic electricity consumers and hydrogen exporters among the scenario dimensions of domestic CO_2_ mitigation, hydrogen export and hydrogen regulation.

Based on the main findings of the impact of temporal regulation on hydrogen exporters and their domestic CO_2_ mitigation identified in Results, we present three possible pathways (aligned with the categorization in Fig. 7) of reaching the aims of hydrogen exports and domestic CO_2_ mitigation complemented with tailored temporal hydrogen regulation recommendations. As outlined in Results, we use static scenarios to reflect different sequences of policy ambition toward shared long-term goals.

First, at quick exports and slow CO_2_ mitigation: Reaching high export levels before adequately mitigating domestic emissions leads to higher total emissions, if no strict temporal hydrogen regulation is in place. Instead, hourly matching reduces domestic electricity cost effectively and redistributes welfare from exporters to domestic consumers. This regulation plays a decisive role in this pathway, even annual or monthly matching triggers the price spillover and energy spillover effects.

Second, at balanced exports and CO_2_ mitigation: A balanced transition of both exports and mitigation offers benefits for both domestic electricity consumers and hydrogen exporters. At a medium (40–60%) CO_2_ mitigation, domestic electricity consumers profit from progressing hydrogen exports. Vice versa, hydrogen exporters benefit from domestic CO_2_ mitigation, if the hydrogen export volume is at 50–100 TWh and the domestic CO_2_ mitigation advances from 0% to a medium range of (40–60%) CO_2_ mitigation. A strict temporal hydrogen regulation enables these co-benefits in this pathway.

Third, at slow exports and quick CO_2_ mitigation: If the transition speed of domestic CO_2_ mitigation is quicker than the scale-up of hydrogen exports, there is no effect of annual or monthly temporal hydrogen regulation on costs for hydrogen exporters or domestic electricity consumers (Fig. 7). The temporal hydrogen regulation plays only a minor role in this pathway, since the electricity system is already highly or fully renewable electricity based and neither price spillover nor energy spillover are effective.

The three illustrative pathways offer certain room for manoeuvre in policy-making for various countries and their export ambitions, speed of domestic CO_2_ mitigation and temporal hydrogen regulation. However, these pathways do not reflect additional constraints as limited renewable energy ramp-up rates, the availability of workforce, additional prerequisites such as an effective carbon pricing system for carbon-based Power-to-X products and bureaucratic hurdles among others. A holistic approach and balanced policies are essential to guide the energy transition and support export ambitions effectively.

Furthermore, balancing the interests of hydrogen exporters and domestic electricity consumers involves various dimensions. First, the price of electricity for domestic businesses and households need to be considered to enable a fair discussion and balance of burdens and opportunities. This study demonstrates that electricity prices (and hence the consumer costs) are closely influenced by hydrogen export volumes and temporal hydrogen regulation. The export of hydrogen in combination with strict temporal hydrogen regulation provides the opportunity of decreasing domestic electricity prices, providing opportunities for domestic businesses and households. The chance to boost local economies by exporting hydrogen is also highlighted in Ishmam et al.^30^.

Second, the substantial deployment of solar PV and onshore wind comes along with a substantial land use. Several studies^40,41^ highlight the requirement of local acceptance of renewable energies in Morocco. From a German/European perspective, hydrogen imports are not necessarily the most economic option^42^ but also provide a way out of having to deal with the domestic acceptance of renewable energies. Shifting acceptance challenges to Morocco through renewable energy deployment and expanding hydrogen exports raises important ethical considerations that require thoughtful handling

Third, this study shows the benefits of integrating solar PV and onshore wind into the main grid for both domestic electricity consumers and hydrogen exporters. By integrating the best sites of solar PV and onshore wind, domestic electricity consumers can access the cost-cutting potentials and benefit from lower electricity prices as shown in our results. On the other hand, off-grid hydrogen generation potentially reserves the promising electricity potentials for hydrogen export only. Nonetheless, Tries et al.^43^ show the benefits of hydrogen islanding, offering cost savings for inverters and benefits for power quality. Domestic consumers may profit from these economic opportunities for hydrogen exporters indirectly through corresponding policies. Hydrogen export ambitions (fostered by e.g. the European Union) need not result in disadvantages for the domestic population per se, a proactive transition taking into account the interplay of domestic electricity prices unleashes economic opportunities for both exporters and domestic population.

The sector-coupled energy model as well as the results underly certain limitations. In this study, we leave the option open that the hydrogen for export is further synthesized to hydrogen derivatives as ammonia, Fischer-Tropsch products or sponge iron etc. We do not model the system operation of a further synthesis of export products explicitly, unlike we do for the domestic demands for hydrogen derivatives. Further synthesis of export products and linked transportation modes are investigated in Hampp et al.^28^, Galimova et al.^44^ and Verpoort et al.^45^ but not in the scope of this study. A further synthesis will likely have effects on the system operation, since the synthesis in combination with cheaper storage (e.g. oil storage is cheaper than hydrogen storage^46^) provides an additional flexibility to a certain extent but is limited by the operation flexibility of Fischer-Tropsch or Haber-Bosch processes (see Supplementary Fig. 20).

The constant hydrogen export demand assumed in this study represents pipeline operation or a further hydrogen synthesis. If the export hydrogen is not further synthesized but exported as liquified hydrogen via shipping, there are significant impacts on the energy system to expect. The demand pattern of ship export (landing, loading, travel time) triggers spikes in hydrogen demand, which would need to be buffered by large-scale hydrogen storage or by a corresponding operation of hydrogen electrolysis. As findings of Franzmann et al.^25^ point out, the cost share of hydrogen storage for export is below 5% of the final product costs in solar regions, while slightly higher for wind-dominated regions.

Additionally, the transition of certain sectors is not subject to the optimisation but exogenously defined. Such nodal shifts (e.g. in transport: share of electric vehicles) provide system benefits (e.g. Vehicle-to-Grid) for which an integrated optimisation favours e.g. higher shares of electric vehicles. Recent approaches as Zeyen et al.^8^ improve such caveats by incorporating endogenous learning, but research gaps on the endogenous demand of the transport sector remain. Furthermore, this study achieves national decarbonization under what is effectively a cap-and-trade framework. As such, results may not fully generalize to other policy contexts, such as those driven by subsidies or fixed carbon pricing, which may influence decarbonization dynamics differently.

National climate ambitions and especially the scale-up of hydrogen export require substantial amounts of raw materials (e.g. copper, cement) linked with resource limitations as well as upstream greenhouse gas emissions. As pointed out in ref. ^47^ in a global analysis, most raw material limitations required for electricity generation do not exceed geological reserves. However, material demands of e.g. electrolysers are not within the scope of Wang et al.^47^.

The cost of water through desalination and transport has a single cost for the whole country. However, the transportation costs highly depend on the terrain as pointed out by Caldera et al.^48^, hence the water costs are higher in more remote areas which is not taken into account in this study. In contrast, the effect of a higher spatial resolution of water costs is assumed to be minor, since the water desalination and transport costs do not contribute substantially to the cost of hydrogen as emphasized in ref. ^28^.

Another limitation of this study concerns the market structure in the Moroccan electricity sector, particularly regarding the integration of renewable energy. While Morocco is progressively moving toward market liberalization, with increasing penetration of renewable electricity and independent power producers, the electricity market is still partially regulated^49^. However, we want to clarify that our study does not model a liberalized market, in which individual market participants maximize their profits in equilibrium. Instead, we assume a central planner’s perspective. In a perfectly competitive market with perfect information, free entry and exit, many participants, and no transaction costs or market failures, the equilibrium reached by price-taking firms and utility-maximizing consumers coincides with the solution a benevolent central planner would choose to minimize total system costs. This is consistent with the First Fundamental Welfare Theorem^50,51^. However, in real-world liberalized markets, these assumptions are often not fully valid: some actors may lack full information, some generators may have market power, and various government interventions can move us away from equilibrium. Despite the fact that the conditions for equivalence between central planner and market outcomes are not fully satisfied in practice, we argue that our study remains relevant for future scenarios, where these conditions may increasingly hold as markets evolve towards liberalization globally^52–55^. In this context, we emphasize that the effect of temporal hydrogen regulation on domestic electricity consumers observed in the section Results is only valid where the market design is such that market prices are passed through to electricity consumers.

Additionally, our model employs a normative scenario approach, which assumes that energy systems are designed to achieve socially optimal outcomes under given constraints (e.g., cost minimization, emissions reduction). While this approach is commonly used in energy system models^2,3,8,31,32,36,39,56–64^, it does not fully capture the complexity of real-world market dynamics, where decentralized decision-making by multiple market participants plays a critical role. Nevertheless, by including endogenous prices to coordinate decentralized actors, our model provides a representation of market-based coordination. Thus, the results should be interpreted as idealized scenarios that highlight possible pathways, with actual market outcomes likely to differ due to a variety of economic and behavioural factors as outlined in Tan et al.^51^.

Concluding, our study shows the importance of integrated scenario analysis combining hydrogen exports, domestic CO_2_ mitigation and temporal hydrogen regulation. At a medium (40–60%) CO_2_ mitigation, domestic electricity consumers profit from progressing hydrogen exports. Vice versa, hydrogen exporters benefit from domestic CO_2_ mitigation, if the hydrogen export volume is at 50–100 TWh and the domestic CO_2_ mitigation advances from 0% to a medium range of (40–60%) CO_2_ mitigation. However, we show that there are also risks with respect to rising domestic electricity prices and find that hydrogen regulation via temporal matching is a decisive instrument to protect domestic consumers. Hourly matching decreases the cost for domestic electricity consumers (up to 31%) while the effect on hydrogen exports is minimal. Temporal hydrogen regulation can effectively hedge domestic electricity consumers against rising prices across mitigation and export scenarios.

This study contributes to the investigation of implications and benefits of hydrogen exports for domestic electricity consumers. Even though temporal hydrogen regulation hedges domestic electricity consumers against rising prices, further implications of hydrogen exporters on the domestic population (land use, competing renewable electricity resources, environmental concerns of desalination) are not within the scope of this study.

In summary, our study underscores that hydrogen export ambitions, as encouraged by entities like the European Union, is in favour of domestic electricity consumers at certain export quantities, if the appropriate hydrogen regulation is in place. A proactive and comprehensive transition strategy, considering the interplay between hydrogen exports and domestic CO_2_ mitigation, is key to unlocking economic opportunities for both hydrogen exporters and the domestic population.

Apart from reducing greenhouse gas emissions, temporal hydrogen regulation is a decisive policy instrument in steering the welfare (re-)distribution between i) hydrogen exporting firms and hydrogen importing countries and ii) hydrogen exporting firms and domestic electricity consumers. A fair balance can increase acceptance across actors, is crucial for a sustainable energy transition in Morocco and offers valuable insights for further countries facing similar energy challenges globally.

## Methods

### Sector-coupled energy model

The sector-coupled energy model of Morocco is based on the global electricity model PyPSA-Earth^60^ and the sector-coupling extension^65^ using linear optimisation and overnight scenarios. This capacity extension model optimises the generation, transmission and storage capacities of Morocco’s energy system by minimising the the total annualised system costs constrained by e.g. climate targets or green hydrogen policies. A more detailed mathematical problem formulation is provided in the Supplementary Method 1: Mathematical model formulation, and a system overview is provided in Supplementary Fig. 1. The model is based on the economic year of 2030, optimizing the time span of a full year. We have chosen the year 2030 to account for the relevance of hydrogen exports and domestic CO_2_ mitigation in the near-term. However, we do not expect or imply that both substantial hydrogen export volumes and the domestic CO_2_ mitigation fully materialize by the year 2030 given the time span required for the ramp up of the required technologies (Renewable electricity, hydrogen electrolyis, etc.). By fixing the modelling year to 2030 instead of modelling transformation pathways, we can isolate the effects of 0–100% mitigation (to today) and 1–120 TWh hydrogen export and contextualize our results in a comparable setting. However, this fixed-year approach has limitations. Modelling actual transition pathways (instead of a fixed year) accounts for evolving factors over time, such as technology costs and regulatory shifts. In comparison, Müller et al.^31^ employs such an approach by exogenously defining the final energy demand, hydrogen exports and emission constraint across a transition path up to 2050. Based on these input parameters, the cost-optimal power sector is modelled endogenously. This allows for a more detailed exploration of transition pathways, and enables insights of reaching (annual) climate goals under a (cumulative) CO_2_ budget.

#### Energy demand

To obtain the annual energy demand, we use the workflow presented by Abdel-Khalek et al.^65^. In a first step, the annual energy demand is obtained from the United Nations Statistics Database^66^. The latest available data is from 2020, since these energy balances are likely to show irregularities due to COVID-19 implications, the base year in this study is 2019. Based on the annual energy demand from 2019, the energy demand of 2030 is projected according to efficiency gains and activity growth rates similar to Müller et al.^67^. In a second step, the annual and national energy demand is distributed according to the production sites of Morocco. In case of subsectors where no spatially resolved production sites are available (e.g. paper industry), the demand is distributed according to GDP. Third, the annual but locationally resolved energy demand is converted to hourly demands. In this study, a constant hourly demand of the industry and agriculture sector is assumed whereas residential, services and transport are temporally resolved based on time series^36,65,68^. The resulting temporal and spatial energy demands of the sectors (i) residential, (ii) services, (iii) industry, (iv) transport (road, rail, aviation and navigation) and (v) agriculture are integrated in the sector coupled energy model, including the energy carriers electricity, hydrogen, natural gas, biomass, oil, and heat, as well as carbon dioxide in the form of emissions and feed-stock for the synthesis of other carriers (see Supplementary Fig. 19). The domestic energy services per sector remain constant throughout the scenarios presented in the Introduction, regardless of endogenous hydrogen export volumes or national carbon emission reductions. However, while energy services (e.g., demand for transportation or heating) are fixed, the primary and final energy consumption are subject to CO_2_ mitigation targets. To reflect the impact of increasing shares of BEVs in the Moroccan energy system, the share of BEVs is linked to emission reduction ambitions (see Supplementary Fig. 2) from 2% (today’s levels) up to a share of 88% at 100% domestic CO_2_ mitigation in accordance with Rim et al.^69^. In the case of low domestic CO_2_ mitigation, a low BEV share results in a high fuel demand for e.g. Internal Combustion Engine Cars, as observable in Supplementary Fig. 5. This reflects a constant energy service demand (transportation) while the primary energy is subject to CO_2_ mitigation.

#### Renewable energy sources

The sector-coupled energy model follows the data pipeline of Parzen et al.^60^ by incorporating solar PV, onshore wind and hydro power using the open source package Atlite^70^. Atlite obtains technology-specific time series of the weather year 2013 based on the ERA5 reanalysis data^71^, displayed in Supplementary Fig. 15. In addition to the time series, Atlite calculates land availabilities and linked potentials using the Copernicus Global Land Service^72^.

#### Conventional electricity generation

The conventional electricity generators are obtained from the powerplantmatching tool^73^. Powerplantmatching uses various input sources as OpenStreetMap2022, Global Energy Monitor, IRENA^74–76^ to download, filter and merge the datasets. In this study, powerplantmatching is applied to Morocco delivering the conventional power plants.

#### Electricity networks and gas pipelines

The electricity network is based on the PyPSA-Earth workflow using OpenStreetMap data^75^ as presented in ref. ^60^. First, the data is downloaded, filtered and cleaned. Second, a meshed network dataset including transformers, substations, converters as well as HVAC and HVDC components is build^60^. The brownfield gas pipeline infrastructure of Morocco is not considered in this study. The Maghreb-Europe pipeline passing through Morocco is the only pipeline in operation but currently subject to political disputes^77^. Further proposed projects are excluded due to miniscule capacity compared to Morocco’s energy system (Tendrara LNG Terminal, 100 million cubic metres per year or uncertain commissioning dates (Nigeria-Morocco Pipeline, start year 2046^78^).

### Brownfield model and capacity expansion

**Fig. 8:**
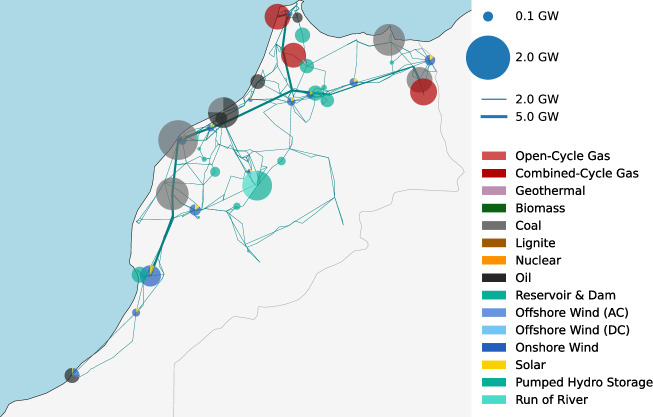
Current capacities of electricity generation and distribution in Morocco. Morocco’s electricity generation portfolio is currently dominated by fossil generation (coal and gas), includes some hydropower plants and increasing but still minor capacities of onshore wind and solar PV. The data is obtained from Parzen et al.^60^ and the visualization is based on Fioriti et al.^93^. Boundaries depicted are based on the Global Administrative Areas^38^ and are intended for illustrative purposes only, not implying territorial claims.

Following the workflow of Parzen et al.^60^, Fig. 8 shows the current capacities of electricity generation and distribution of Morocco. Based on this brownfield electricity system, the sector-coupled energy model allows the capacity expansion of storage, (electricity networks), hydrogen pipelines, renewable and conventional generators.

### Technology and cost assumptions

All technology and cost assumptions are based on the year 2030, taken from the technology-data repository. This study applies version 0.4.0 of the repository, which is available at technology-data v0.4.0. Based on a comprehensive survey of industry and academia in 2024 by Fernandez et al.^79^, which reports a combined Market Risk Premium and Risk-Free Rate of 12.9% for Morocco, we follow their findings and apply a discount rate of 13% in this study. While IRENA^80^ reports a technology-specific interest rate of 9.1% for utility-scale PV in Morocco, we apply a more conservative approach following Fernandez et al.^79^ taking into account possible higher risks of less-established technologies as water electrolysis and hydrogen infrastructure compared to utility-scale solar PV.

### Water supply

Hydrogen production through electrolysis requires fresh water, alternatives as the direct use of high saline water sources are still in the experimental stages^81^. The depletion of freshwater resources and it’s competition with other water uses is a concern, especially in regions such as Morocco which is ranked 27th among the world’s most water-stressed countries^82^. Sea Water Reverse Osmosis emerges as a feasible solution to address this issue. In this model, the additional water cost of 0.80 € m^−3^ through desalination and transport is considered in line with the base scenario for Morocco in 2030 in Caldera et al.^83^. These findings are comparable to Kettani et al.^84^ and Caldera et al.^48^ stating the water costs of 1 $ m^−3^ resp. 0.60–1.50 € m^−3^ for Morocco in 2030. Given a water demand of 9 $$l\,{{{{\rm{kg}}}}}_{{{{{\rm{H}}}}}_{2}}^{-1}$$^28^, the additional costs for electrolysers result in 0.213 € per megawatt-hour (€ MWh^−1^) based on the lower heating value.

However, the challenge of brine disposal presents environmental concerns due to treatment chemicals and high salinity as discussed by Thomann et al.^85^, Dresp et al.^86^ and Tonelli et al.^87^, highlighting the importance of a careful site selection of desalination plants. A minimum distance of four kilometres from marine protected areas is recommended in Thomann et al.^85^.

### Green hydrogen policy

A key constraint on the model is that the hydrogen exported from Morocco requires to meet sustainability criteria (green hydrogen). A variety of studies looks into various dimensions (temporal, geographical, electricity origin) of green hydrogen and their trade-offs^32–34^. Envisioning hydrogen offtakers from the European Union, the green hydrogen constraint applied in this study aligns with the Delegated regulation on Union methodology for RFNBOs of the European Commission^88^ defining green hydrogen based on the following criteria: (i) additionality: The electricity demand of electrolysers must be provided by additional renewable electricity power generation (less than 3 years before the installation of electrolysers, from 1.1.2028 onwards), and (ii) temporal correlation: Monthly matching until 31.12.2029, and hourly matching from 1.1.2030 onwards, and (iii) geographical correlation.

Those criteria are required for Power Purchase Agreements (PPA) with RE-installation. Apart from the PPAs, the delegated act also considers the possibility of: (i) direct connection of renewable electricity and electrolysers, or (ii) high share of renewable electricity in power mix (>90%) or the (iii) avoidance of renewable electricity curtailment to qualify as green hydrogen. These further options are not considered here, since a system-integrated electrolysis in a power system of a (current) share of renewable electricity below 90% is the scope of this study. The sole focus on renewable electricity curtailment and hence low total hydrogen volumes is not applicable due to expected hydrogen exports of up to multiple times of Morocco’s domestic electricity demand. The green hydrogen definition of the European Commission prior to 1.1.2028 is not applied in this study, since relevant volumes of hydrogen export are expected to materialize from 2028 onwards and is excluded by the scope of this study presented in the Introduction).

### Hydrogen export

The amount of hydrogen to be exported or further synthesized for export is implemented as an exogenous parameter in the range of 1–120 TWh, with sensitivity analysis up to 200 TWh presented in the Supplementary Discussion 3. The system boundary is the country border of Morocco, hence transport options (as shipping or pipeline) are not considered in detail. The profile for hydrogen (or derivatives) export assumed in this study is constant. This represents the operation of pipeline exports as well as the operation of a further hydrogen synthesis with limited flexibility. The export of hydrogen is allowed via a range of ports in Morocco.

The energy system model allows an endogenous spatial export decision, meaning that the total demand (and export profiles) are exogenous, but the model chooses the cost-optimal export location(s). At each port, the model has the option to build a hydrogen underground or steel tank depending on domestic geological conditions.

For the electrolysis, we use alkaline electrolysers due to their maturity, durability, and lower costs compared to proton exchange membrane electrolysers^46,89^. Alkaline electrolysers are well-suited for large-scale use, though they have slower start-up times and a more limited operating range than proton exchange membrane electrolysers^89,90^, which, in contrast, have shorter cell lifetimes as pointed out in Staffell et al.^89^.

## Supplementary information

Supplementary Information

Transparent Peer Review file

## Data Availability

The model results generated in this study have been deposited on Zenodo 10.5281/zenodo.10951650^91^. The data are openly available under a CC-BY-4.0 license. The technology data used in this study are available in the technology-data repository. This study applies version 0.4.0 of the dataset, accessible at https://github.com/PyPSA/technology-data/blob/v0.4.0/outputs/costs_2030.csv.

## Code availability

The code to reproduce the experiments is available on GitHub https://github.com/energyLS/aldehyde. The code has been archived on Zenodo under 10.5281/zenodo.15845805^92^. We also refer to the documentations of PyPSA https://pypsa.readthedocs.io and PyPSA-Earth https://pypsa-earth.readthedocs.io, and the code of PyPSA-Earth-Sec https://github.com/pypsa-meets-earth/pypsa-earth-sec.
