# Supplementary material for: The impact of temporal hydrogen regulation on hydrogen exporters and their domestic energy transition
Source: Nat Commun. 2025 Aug 12;16:7486. doi: 10.1038/s41467-025-62873-w (PMC12343794; doi:10.1038/s41467-025-62873-w)
Supplement: Supplementary file 1 — Supplementary Information [file 41467_2025_62873_MOESM1_ESM.pdf]

Supplementary Information for the impact of temporal hydrogen regulation on hydrogen exporters and their domestic energy transition

Leon Schumm[1,2,*], Hazem Abdel-Khalek[3,4,5], Tom Brown[2], Falko Ueckerdt[6], Michael Sterner[1], Maximilian Parzen[5,7], Davide Fioriti[8]

[*] *Corresponding author. Email: leon1.schumm@oth-regensburg.de*

[1]*Research Center on Energy Transmission and Storage (FENES), Faculty of Electrical and Information Technology, University of Applied Sciences (OTH) Regensburg, Seybothstr. 2, 93053 Regensburg, Germany*
[2]*Department of Digital Transformation in Energy Systems, Technische Universität Berlin, Einsteinufer 25 (TA 8), 10587 Berlin, Germany*
[3]*Fraunhofer Research Institution for Energy Infrastructures and Geothermal Systems IEG, Gulbener Straße 23, 03046 Cottbus, Germany*
[4]*Albert-Ludwigs Universität Freiburg, Faculty of Environment and Natural Resources, Tennenbacher Str. 4, 79106 Freiburg im Breisgau, Germany*
[5]*Open Energy Transition, Königsallee 52, 95448 Bayreuth, Germany*
[6]*Potsdam Institute for Climate Impact Research, Telegrafenberg, 14473 Potsdam, Germany*
[7]*University of Edinburgh, Institute for Energy Systems, EH9 3DW Edinburgh, United Kingdom*
[8]*University of Pisa, Department of Energy Systems, Territory and Construction Engineering, Largo Lucio Lazzarino, 56122 Pisa, Italy*

Supplementary Items List

Supplementary Method 1: Mathematical model formulation 3

Supplementary Method 2: System overview 4

Supplementary Method 3: Diffusion of battery electric vehicles 5

Supplementary Method 4: Scenario groups 6

Supplementary Discussion 1: Temporal hydrogen regulation 7

Supplementary Discussion 2: Hydrogen cost breakdown 9

Supplementary Discussion 3: High export sensitivity 12

Supplementary Figures 14

Supplementary References 20

Supplementary Method 1: Mathematical model formulation

The core mathematical model formulation of the sector-coupled energy model is based on the European model PyPSA-Eur described in Neumann et al.[1] and the global model PyPSA-Earth and it's sector-coupled extension presented in Abdel-Khalek et al.[2]. The objective of the optimisation is minimizing the annual energy system costs including annualized investment costs and operational expenditures of generation, storage, transmission and conversion infrastructure[1] and is described in Abdel-Khalek et al.[2] as Supplementary Equation (1):

$$
\begin{aligned}
\min_{\substack{G_{n,k},E_{n,k},C_{n,k},F_{l,c}\\ \dot{G}_{n,k,t},\dot{E}_{n,k,t},\dot{C}_{n,k,t},\dot{F}_{l,c,t}}} & \overbrace{\sum_{n\in N}\sum_{k\in K} c_k G_{n,k} + \sum_{n\in N}\sum_{k\in K} c_k E_{n,k} + \sum_{n\in N}\sum_{k\in K} c_k C_{n,k} + \sum_{l\in L} c_l F_{l,c}}^{\text{annualized capital costs}} \\
& \underbrace{+ \sum_{t\in T}\left[w_t \left(\sum_{n\in N}\sum_{k\in K} o_k \dot{G}_{n,k,t} + \sum_{n\in N}\sum_{k\in K} o_k \dot{E}_{n,k,t} + \sum_{n\in N}\sum_{k\in K} o_k \dot{C}_{n,k,t} + \sum_{l\in L} o_l \dot{F}_{l,c,t} \right)\right]}_{\text{marginal costs}}
\end{aligned}
\tag{1}
$$

where the decision variables cover both the optimal capacity of the technology and the optimal hourly dispatch. The model operates on a set of nodes N, set of carriers C, set of technologies K, set of inter-nodal connections L, and a timeframe T, which in this study is a full year. $G_{n,k}$ denotes the optimal generation capacity of an asset of technology k at node n and $\dot{G}_{n,k,t}$ denotes the optimal dispatch of that asset at timestep t. Similarly, the decision variables of storage, converters and energy flow are presented as $E_{n,k}$, $C_{n,k}$ and $F_{l,c}$ respectively where k uniquely identifies the technology for each category, c uniquely identifies the carrier, and l uniquely identifies the inter-nodal connections. The coefficients represent the different cost components: c_k represents the annualized capital cost in technology k while o_k represent the operating costs. The w_t weight variable represents the duration of the timestep t and is decided by the model configuration, in this study it is three-hourly.

In addition to the core mathematical formulation, the model consists of linear constraints on the spatio-temporal availability of renewable energy sources, storage consistency equations, generation, storage, conversion and transmission infrastructure, a limit for CO_2 emissions and transmission and a multi-period linearised optimal power flow (LOPF) formulation. All constraints are described in detail in Neumann et al.[1] resulting in a linear problem (LP).

Supplementary Method 2: System overview

Supplementary Fig. 1 shows the system overview of the applied model as presented in Abdel-Khalek et al.[2]. The sector-coupled model includes various energy carriers (liquid fossil, biomass, heat, electricity, hydrogen and natural gas), multiple sectors and network infrastructure (power transmission lines, hydrogen pipelines and natural gas network)[2].

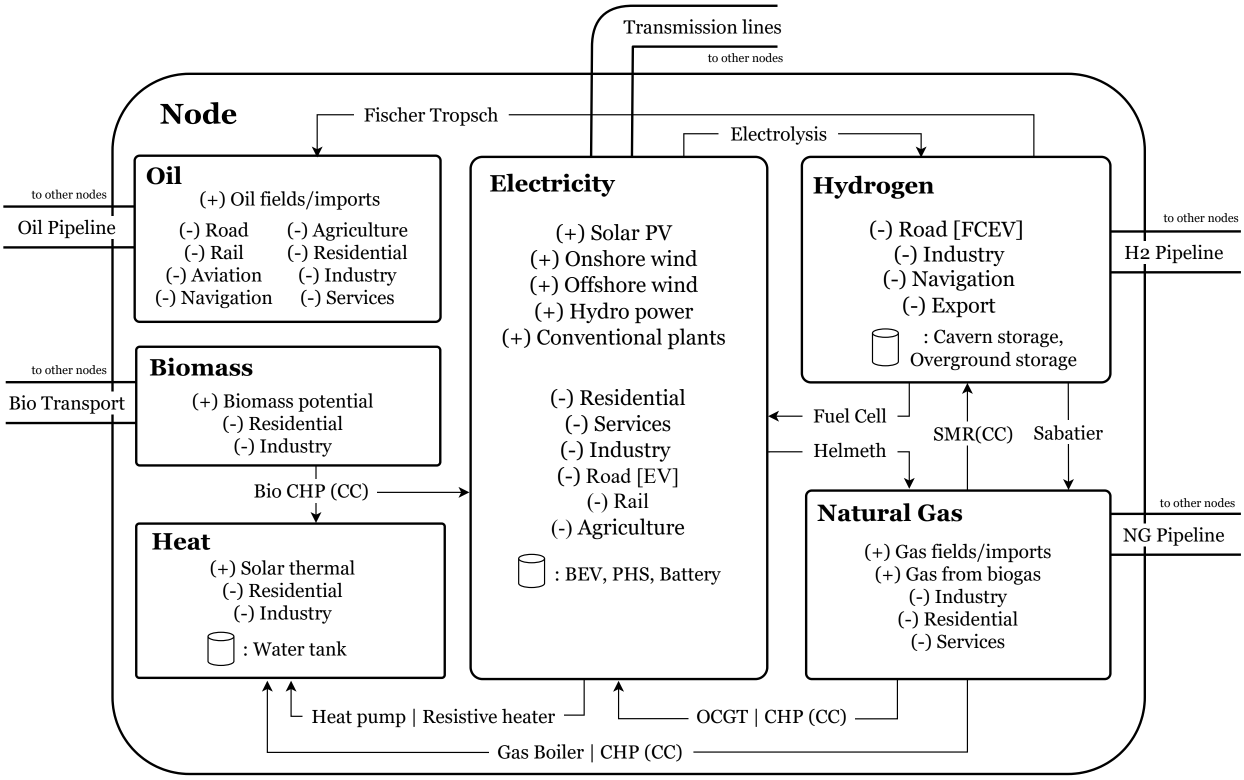

Supplementary Fig. 1: System overview of the sector-coupled model. It includes various energy carriers (liquid fossil, biomass, heat, electricity, hydrogen and natural gas), multiple sectors and network infrastructure (power transmission lines, hydrogen pipelines and natural gas network)[2]. Note, Supplementary Fig. 1 represents only a single node of the multi-nodal energy system. CHP = combined heat and power; CC = carbon capture; OCGT = open cycle gas turbine.

Supplementary Method 3: Diffusion of battery electric vehicles

Supplementary Fig. 2 shows the diffusion of battery electric vehicles depending on the domestic CO_2 mitigation, displaying electric vehicle stock in Morocco. The share rises from 2% (today's levels) up to a share of 88% at 100% domestic CO_2 mitigation in accordance with Rim et al.[3].

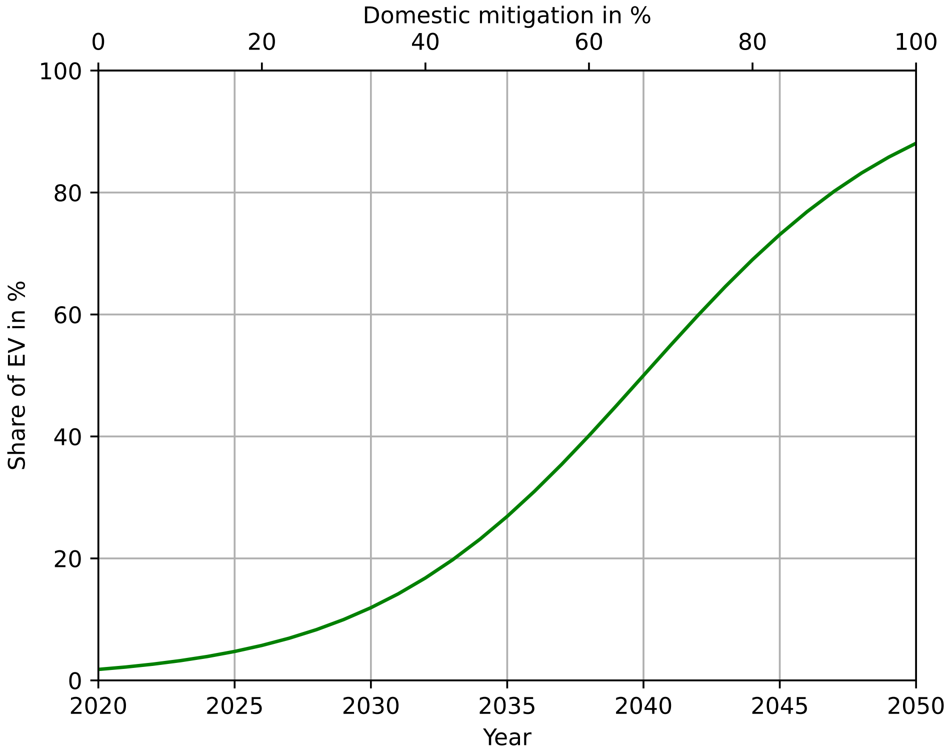

Supplementary Fig. 2: Market diffusion of Battery Electricity Vehicles (EV) in Morocco. The market diffusion is synthesized based on an s-curve with a growth rate $k = 0.2$ and inflection point $x_0 = 2040$. The share rises from 2% (today's levels) up to a share of 88% at 100% domestic CO_2 mitigation.

Supplementary Method 4: Scenario groups

Supplementary Fig. 3 shows the categorization of the mitigation-export scenarios into three stylized transition strategies: i) quick exports and slow CO_2 mitigation, ii) balanced exports and CO_2 mitigation and iii) slow exports and quick CO_2 mitigation. These groupings reflect different sequences of policy ambition evaluated at a fixed modeling year. Supplementary Fig. 3a shows the categorization for export scenarios up to 120 TWh as displayed in Figure 7. Supplementary Fig. 3b extends this categorization taking export quantities up to 200 TWh into account, as basis for the sensitivity analysis on high export scenarios shown in Supplementary Fig. 11.

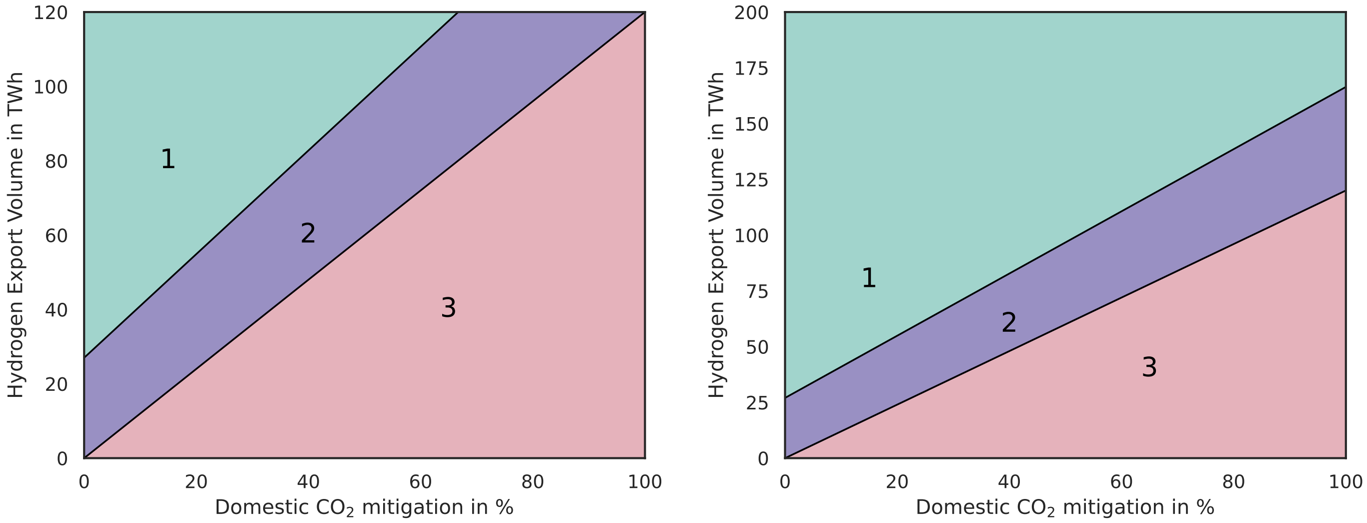

(a) Scenario selection up to 120 TWh

(b) Scenario selection up to 200 TWh

Supplementary Fig. 3: Scenario grouping of three stylized transition strategies. Scenario grouping of all scenarios up to 120 TWh (Fig. 3a) and the scenario grouping including the sensitivity on hydrogen exports of up to 200 TWh (Fig. 3b).

Supplementary Discussion 1: Temporal hydrogen regulation

Electricity supply and demand without temporal hydrogen regulation. Supplementary Fig. 4b shows the effect of increasing domestic CO_2 mitigation at constant (1 TWh) hydrogen exports without temporal matching. Since there are no substantial hydrogen exports, the difference of temporal matching (displayed in Figure 3) is only marginal. Supplementary Fig. 4a displays the hydrogen export ramp up from 1–120 TWh at 0% domestic CO_2 mitigation without temporal regulation. If the no temporal regulation is applied, the dispatch of coal and gas generators increase, which is in contrast to the hydrogen export ramp up with hourly matching, displayed in Figure 3.

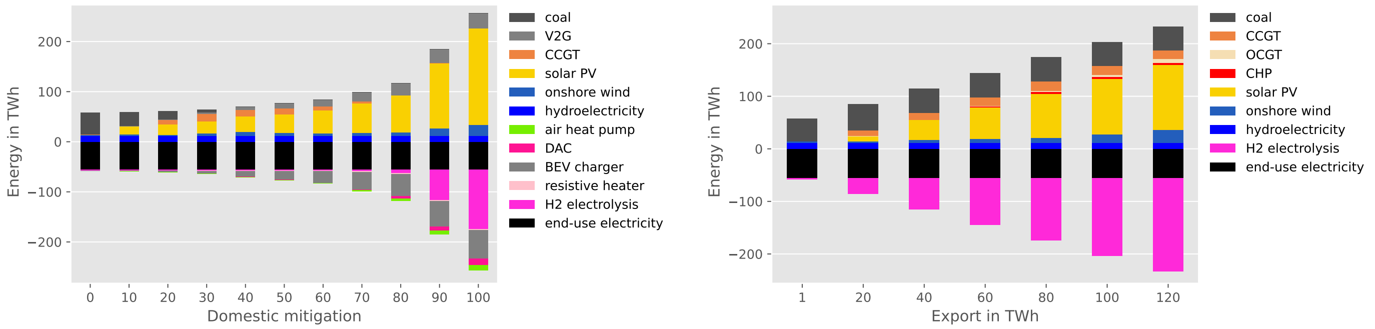

(a) Fixed (1 TWh) export and 0–100% domestic CO_2 mitigation

(b) Fixed (0%) domestic CO_2 mitigation and 1–120 TWh export

Supplementary Fig. 4: Electricity supply and demand without temporal hydrogen regulation at fixed export levels and increasing domestic CO_2 mitigation, and vice versa. In (a), increasing domestic CO_2 mitigation first phases out carbon-intensive coal generation in favor of combined cycle gas turbines (CCGT), at medium to high domestic CO_2 mitigation the electricity system is fully renewable supported by flexibility through Vehicle-to-Grid (V2G) and sector coupling. Increasing electricity demands include Battery Electric Vehicles (BEV) and hydrogen generation for other sectors. In (b), increasing hydrogen exports the additional electricity required for hydrogen electrolysis is covered by onshore wind and solar PV, as well as coal and gas power plants since no temporal hydrogen regulation is in place. DAC = direct air capture; PV = photovoltaics.

Effects of temporal matching in a high export and low CO_2 mitigation scenario on total system costs. Supplementary Fig. 5 displays the total system costs at 120 TWh export and 0% domestic CO_2 mitigation. Stricter temporal hydrogen regulation mainly increases the total CAPEX of additional solar PV, electrolysis and hydrogen storage. In return, the OPEX of fossil generation (mainly gas), decreases. The large share of oil OPEX is independent of temporal hydrogen regulation, since these costs are mainly linked to combustion engine cars with demands independent of temporal hydrogen regulation.

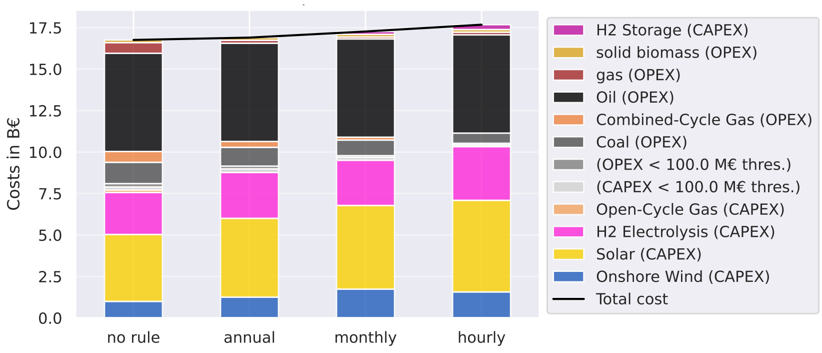

Supplementary Fig. 5: Total system costs at 120 TWh export and 0% domestic CO_2 mitigation. Stricter temporal hydrogen regulation mainly increases the total CAPEX (Capital Expenditures) of additional solar PV, electrolysis and hydrogen storage. In return, the OPEX (Operational Expenditures) of fossil generation (mainly gas), decreases. The large share of oil OPEX is independent of temporal hydrogen regulation, since these costs are mainly linked to combustion engine cars with demands independent of temporal hydrogen regulation.

Cost for domestic electricity consumers and hydrogen exporters without hydrogen regulation. Supplementary Fig. 6 shows, that increasing hydrogen exports quickly in a system that is still dominated by fossil fuels substantially raises market-based costs for domestic electricity consumers, if green hydrogen production is not regulated. At low hydrogen exports (1-20 TWh), an increase of domestic CO_2 mitigation increases the cost for hydrogen exporters.

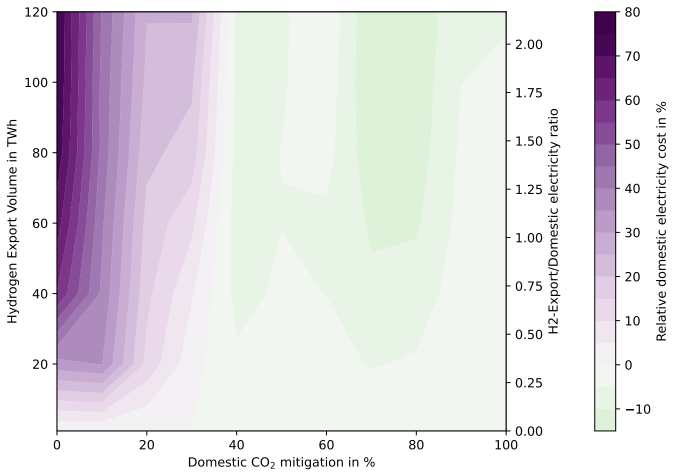

(a) Impact of hydrogen exports on domestic interests: Relative cost of electricity for domestic consumers (normalized to 1 TWh hydrogen export)

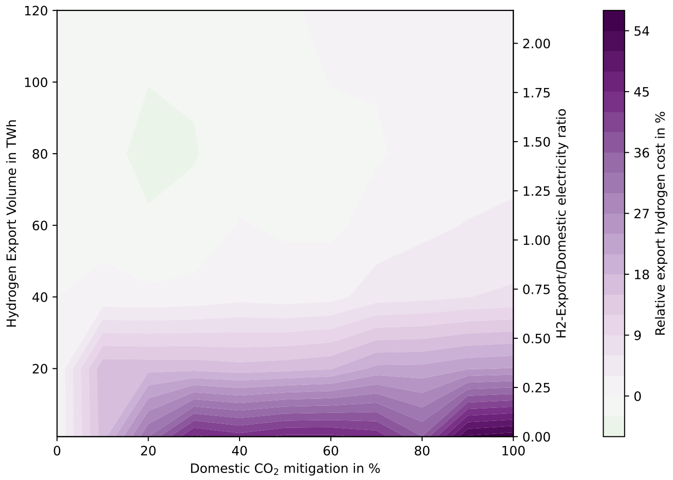

(b) Impact of domestic CO_2 mitigation on hydrogen exporter interests: Relative cost of hydrogen for exporters (normalized to 0% domestic CO_2 mitigation)

Supplementary Fig. 6: The effects of hydrogen exports on domestic electricity consumers and domestic CO_2 mitigation on hydrogen exporters without temporal hydrogen regulation. Supplementary Fig. 6a shows the effect of hydrogen exports on domestic electricity cost. Therefore, the effect of hydrogen exports is isolated by normalizing the costs to 1 TWh hydrogen export in each column. This approach allows for a vertical interpretation in Supplementary Fig. 6a only, displaying the effects of hydrogen exports (vertical) at a certain domestic CO_2 mitigation level. Supplementary Fig. 6b shows the effect of domestic CO_2 mitigation on hydrogen export cost. Therefore, the effect of domestic CO_2 mitigation is isolated by normalizing the costs to 0% CO_2 mitigation in each row. This approach allows for a horizontal interpretation in Supplementary Fig. 6b only, displaying the effects of domestic CO_2 mitigation (horizontal) at a certain hydrogen export quantity. Increasing hydrogen exports quickly in a system that is still dominated by fossil fuels substantially raises market-based costs for domestic electricity consumers, if green hydrogen production is not regulated. At low hydrogen exports (1-20 TWh), an increase of domestic CO_2 mitigation increases the cost for hydrogen exporters.

Supplementary Discussion 2: Hydrogen cost breakdown

The endogenous price of hydrogen depends on single cost components as well as the temporal hydrogen regulation. Supplementary Fig. 7 displays the cost breakdown of hydrogen whereas the combined cost in the Supplementary Fig. 7e differs from the price of hydrogen in the Supplementary Fig. 7f. This is due to the temporal hydrogen regulation, the costs for the required installation of additional renewable electricity capacities are not reflected in the cost breakdown but included in the price of hydrogen. This effect is most striking in high export and low domestic CO_2 mitigation scenarios. In constrast, if there is no temporal hydrogen regulation applied, both hydrogen cost (see Supplementary Fig. 8e) and hydrogen price (see Supplementary Fig. 8f) show similar trends across all domestic CO_2 mitigation and export scenarios.

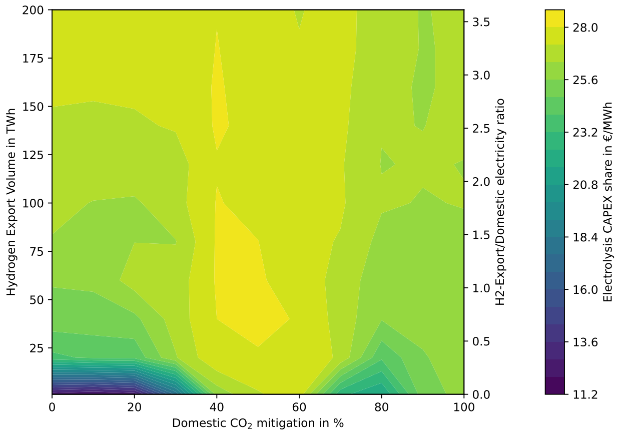

(a) CAPEX share of hydrogen costs

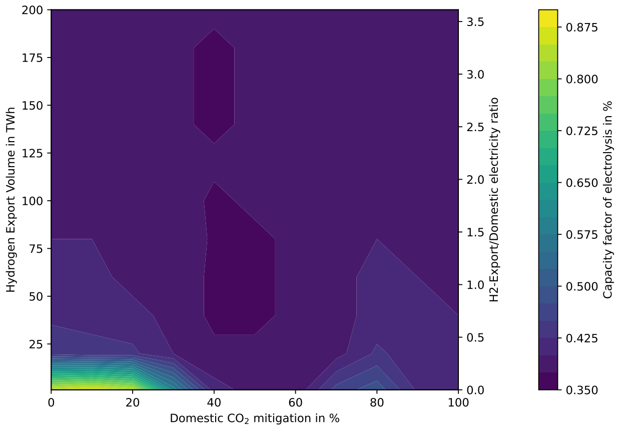

(b) Electrolysis capacity factor

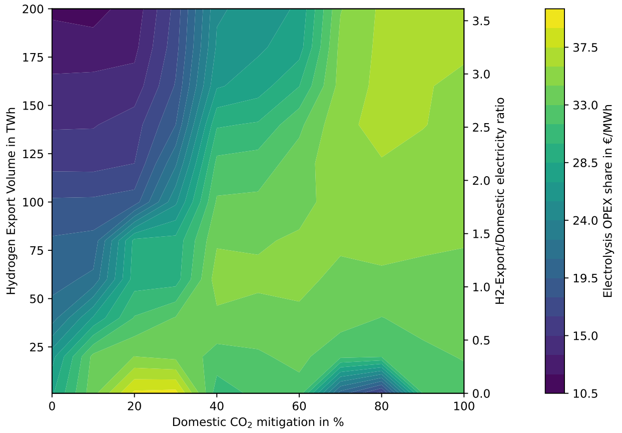

(c) OPEX share of hydrogen costs

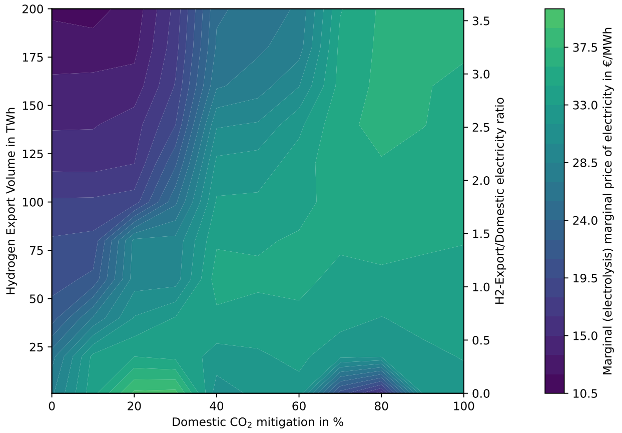

(d) Price of electricity (electrolysis demand weighted)

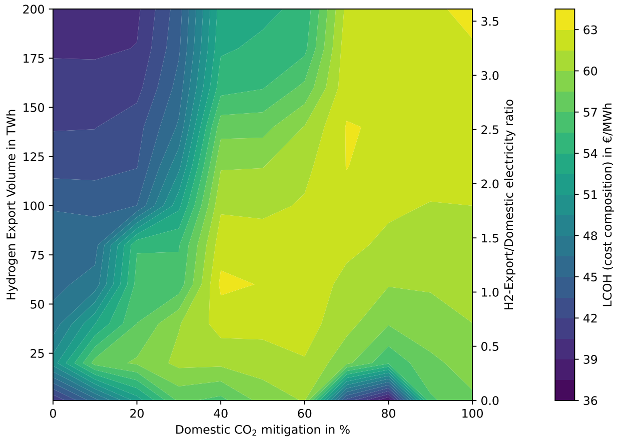

(e) Total hydrogen cost

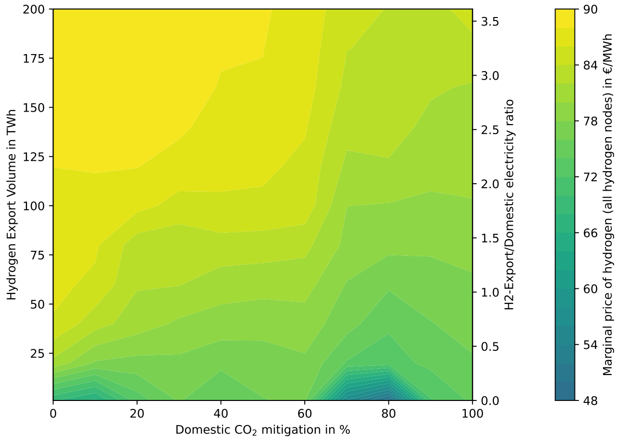

(f) Hydrogen price

Supplementary Fig. 7: Cost components of hydrogen electrolysis and the respective main influence factors on it with hourly temporal matching. The CAPEX (7a) depends on the electrolysis capacity factor (7b), whereas the OPEX (7c) is influenced by the price of electricity for electrolysis (7d). With temporal hydrogen regulation, the cost of hydrogen (7e) is not reflected by the price of hydrogen (7f), which includes the additional constraint of hourly matching and hence the installation of renewable electricity and storage capacities. CAPEX = capital expenditures; OPEX = operational expenditures; LCOH = levelized cost of hydrogen.

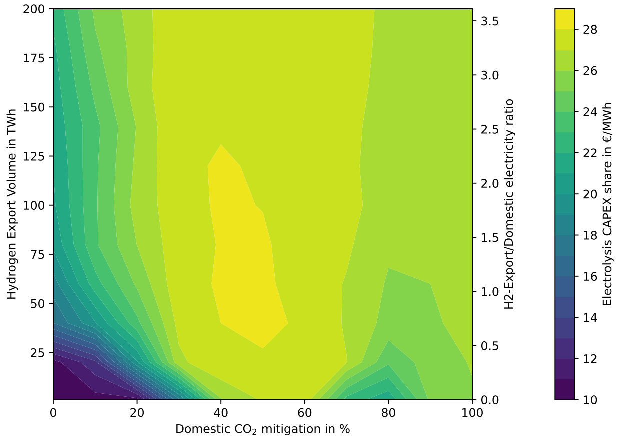

(a) CAPEX share of hydrogen costs

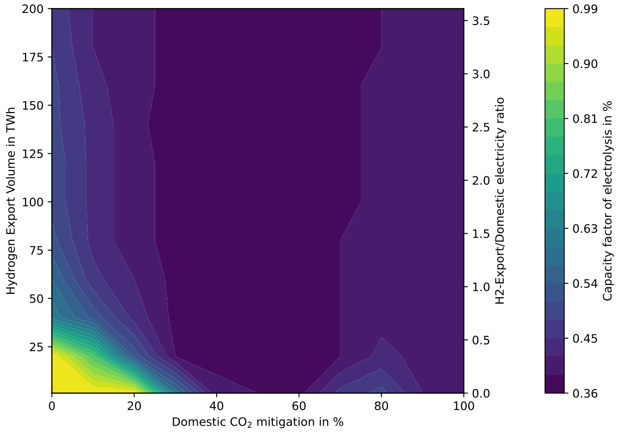

(b) Electrolysis capacity factor

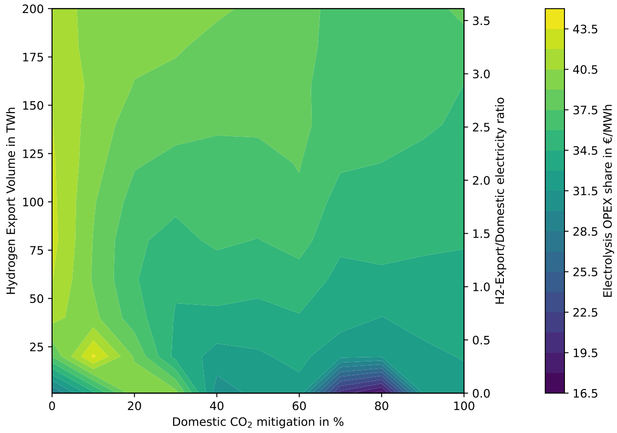

(c) OPEX share of hydrogen costs

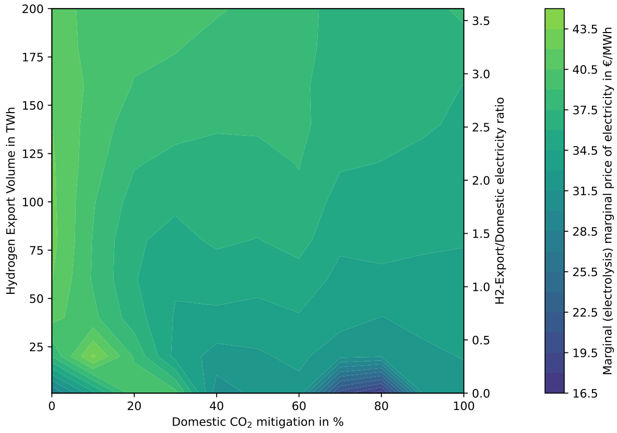

(d) Price of electricity (electrolysis demand weighted)

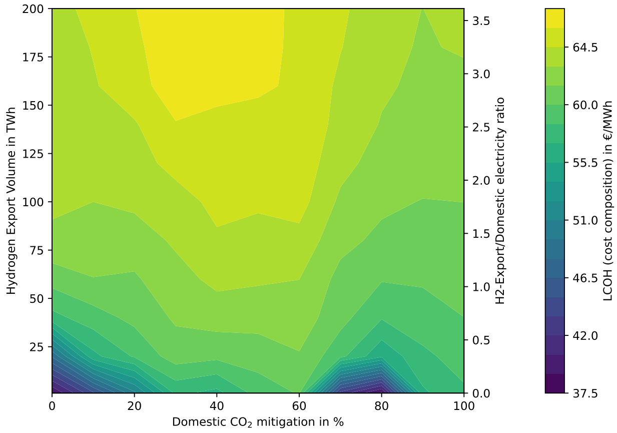

(e) Total hydrogen cost

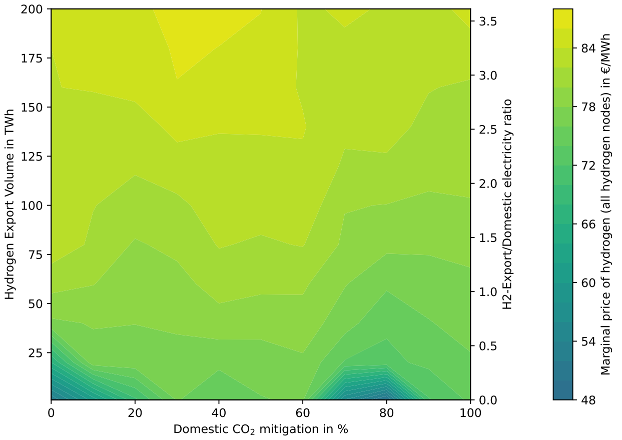

(f) Hydrogen price

Supplementary Fig. 8: Cost components of hydrogen electrolysis and the respective main influence factors on it without temporal hydrogen regulation. The CAPEX (8a) depends on the electrolysis capacity factor (8b), whereas the OPEX (8c) is influenced by the price of electricity for electrolysis (8d). With temporal hydrogen regulation, the cost of hydrogen (8e) is not reflected by the price of hydrogen (8f), which includes the additional constraint of hourly matching and hence the installation of renewable electricity and storage capacities. CAPEX = capital expenditures; OPEX = operational expenditures; LCOH = levelized cost of hydrogen.

Supplementary Discussion 3: High export sensitivity

In our main study, we investigate hydrogen export scenarios up to 120 TWh. Here, we perform a sensitivity analysis where we show the effects of hydrogen export scenarios up to 200 TWh. Supplementary Fig. 9 displays the electricity supply and demand at fixed export levels and increasing domestic CO_2 mitigation (9a) and vice versa (9b). The relative cost of domestic electricity and hydrogen export in high export scenarios (see Supplementary Fig. 10) supports the findings of the main study, as domestic electricity consumers benefit from high hydrogen export especially at low domestic CO_2 mitigation scenarios. When taking high export scenarios (up to 200 TWh) into account, the effect of temporal hydrogen regulation on electricity and hydrogen costs is stronger compared to the main study, as displayed in Supplementary Fig. 11.

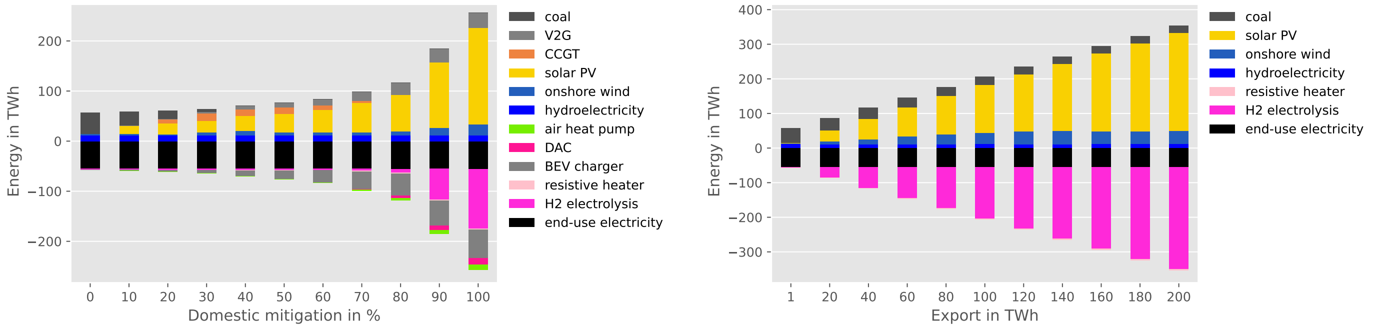

(a) Fixed (1 TWh) export and 0–100% domestic CO_2 mitigation

(b) Fixed (0%) domestic CO_2 mitigation and 1–200 TWh export

Supplementary Fig. 9: Electricity supply and demand at fixed export levels and increasing domestic CO_2 mitigation (9a) and vice versa (9b). Increasing domestic CO_2 mitigation first phases out carbon-intensive coal generation in favor of combined cycle gas turbines (CCGT), at medium to high domestic CO_2 mitigation the electricity system is fully renewable supported by flexibility through Vehicle-to-Grid (V2G) and sector coupling. Increasing electricity demands cover EVs and hydrogen generation for other sectors. At increasing hydrogen exports the additional electricity required for hydrogen electrolysis is covered by onshore wind and solar PV, as imposed by the temporal hydrogen regulation. DAC = direct air capture; PV = photovoltaics.

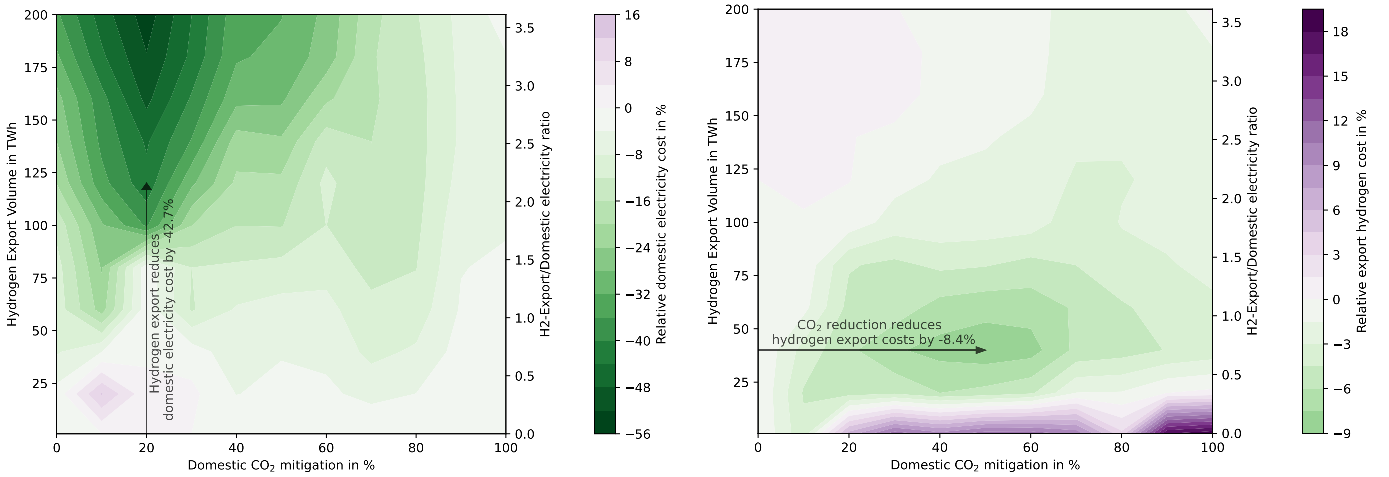

(a) Impact of hydrogen exports on domestic interests: Relative cost of electricity for domestic consumers (normalized to 1 TWh hydrogen export)

(b) Impact of domestic CO_2 mitigation on hydrogen exporter interests: Relative cost of hydrogen for exporters (normalized to 0% domestic CO_2 mitigation)

Supplementary Fig. 10: The effects of hydrogen exports on domestic electricity consumers and domestic CO_2 mitigation on hydrogen exporters. Figure 10a shows the effect of hydrogen (H_2) exports on domestic electricity cost with hourly hydrogen regulation. Therefore, the effect of hydrogen exports is isolated by normalizing the costs to 1 TWh hydrogen export in each column. This approach allows for a vertical interpretation in Figure 10a only, displaying the effects of hydrogen exports (vertical) at a certain domestic CO_2 mitigation level. Figure 10b shows the effect of domestic CO_2 mitigation on hydrogen export cost with hourly hydrogen regulation. Therefore, the effect of domestic CO_2 mitigation is isolated by normalizing the costs to 0% CO_2 mitigation in each row. This approach allows for a horizontal interpretation in Figure 10b only, displaying the effects of domestic CO_2 mitigation (horizontal) at a certain hydrogen export quantity. Domestic electricity consumers profit from increasing hydrogen exports, especially at low domestic CO_2 mitigation and high exports. Hydrogen exporters profit from domestic CO_2 mitigation at medium mitigation efforts.

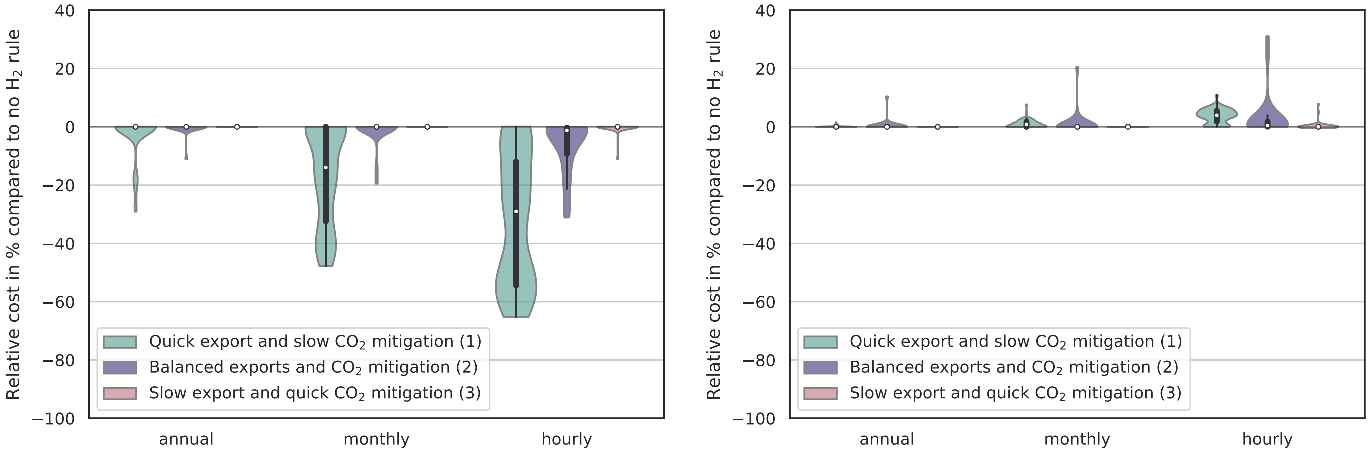

(a) Cost reduction for electricity consumers

(b) Cost increase for hydrogen exporters

Supplementary Fig. 11: Relative change of electricity (Fig. 11a) and hydrogen consumer cost (Fig. 11b) depending on the temporal hydrogen (H_2) regulation. Violin plots indicate median (white dot), interquartile range (thick black bar), and 1.5x interquartile range (thin black bar), using kernel density estimation to show the distribution shape of the mitigation-export scenarios, grouped into different speeds of transformation (1,2,3). Domestic electricity consumers profit across all export and mitigation scenarios but most in the group of slow export and quick CO_2 mitigation scenarios. Hydrogen exporters experience higher cost with stricter temporal hydrogen regulation. The temporal hydrogen regulation regulates the welfare distribution between both groups.

Supplementary Figures

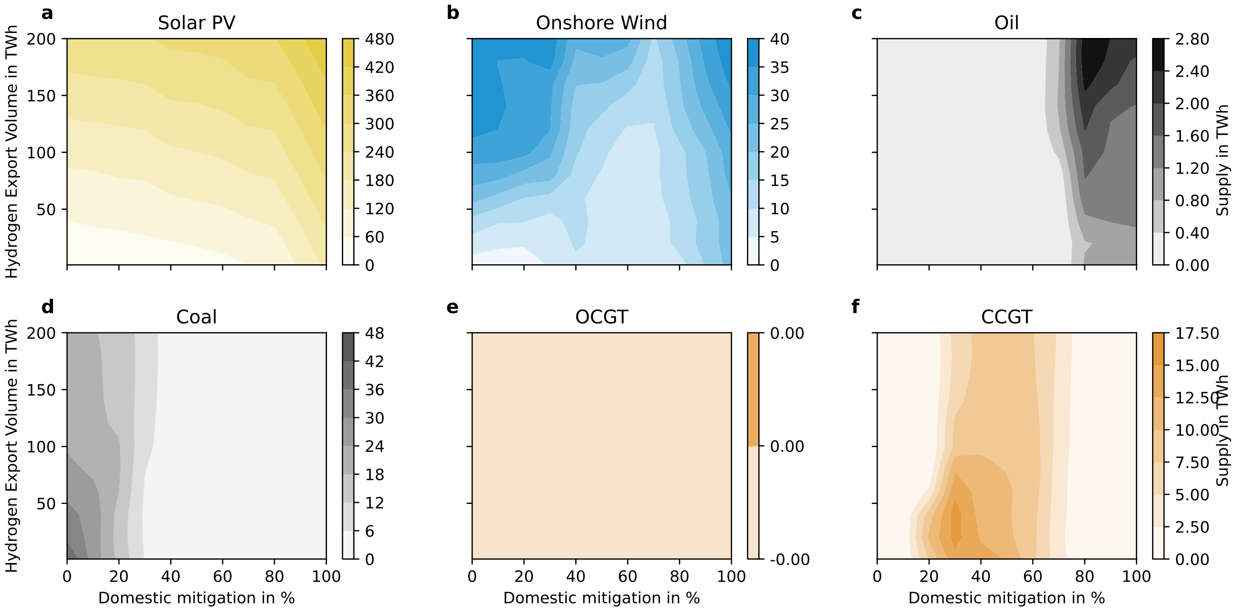

Supplementary Fig. 12: Electricity supply with hourly temporal matching. Increasing the hydrogen export volume requires additional renewable electricity supply (**a,b**), as defined in the additionality criteria. Advancing domestic CO_2 mitigation leads to the phase-out of coal power (**d**), with combined cycle gas turbines (CCGT) serving as a transitional substitute before achieving a fossil-free electricity system (**f**). Both oil generators (**c**) and open cycle gas turbines (OCGT) (**e**) generate below 3 TWh in all scenarios. PV = photovoltaics.

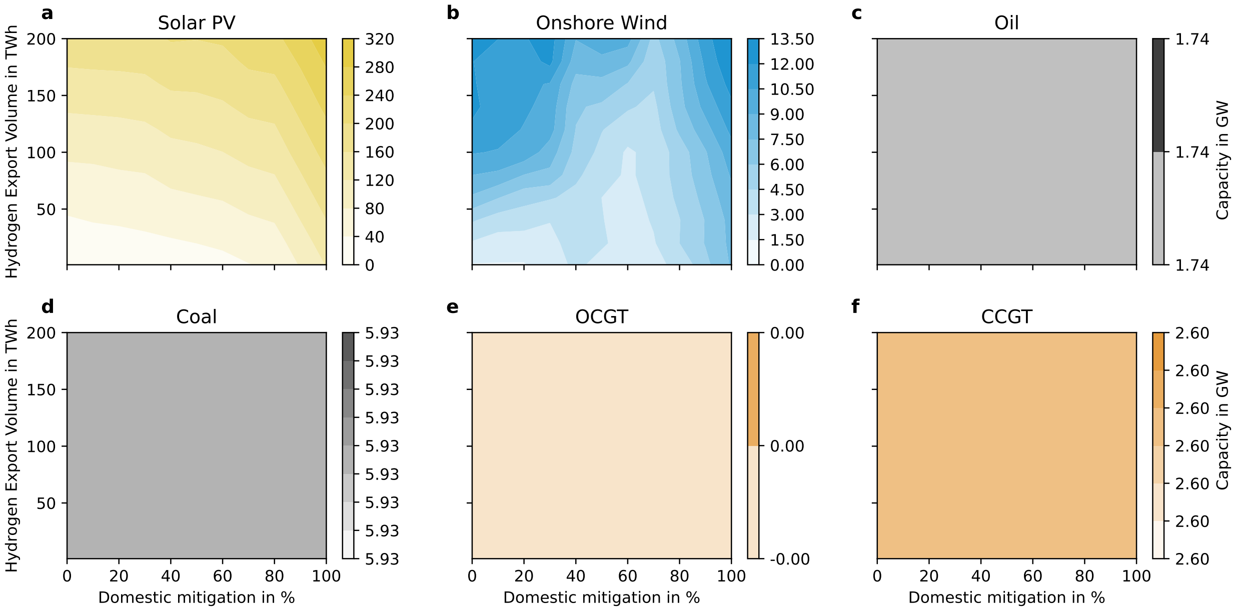

Supplementary Fig. 13: Electricity capacities with hourly temporal matching. Increasing the hydrogen export volume requires additional renewable electricity capacities (**a**,**b**), as defined in the additionality criteria. Fossil capacities (**c**-**f**) remain limited to existing brownfield assets. OCGT = open cycle gas turbine; CCGT = combined cycle gas tubine; PV = photovoltaics.

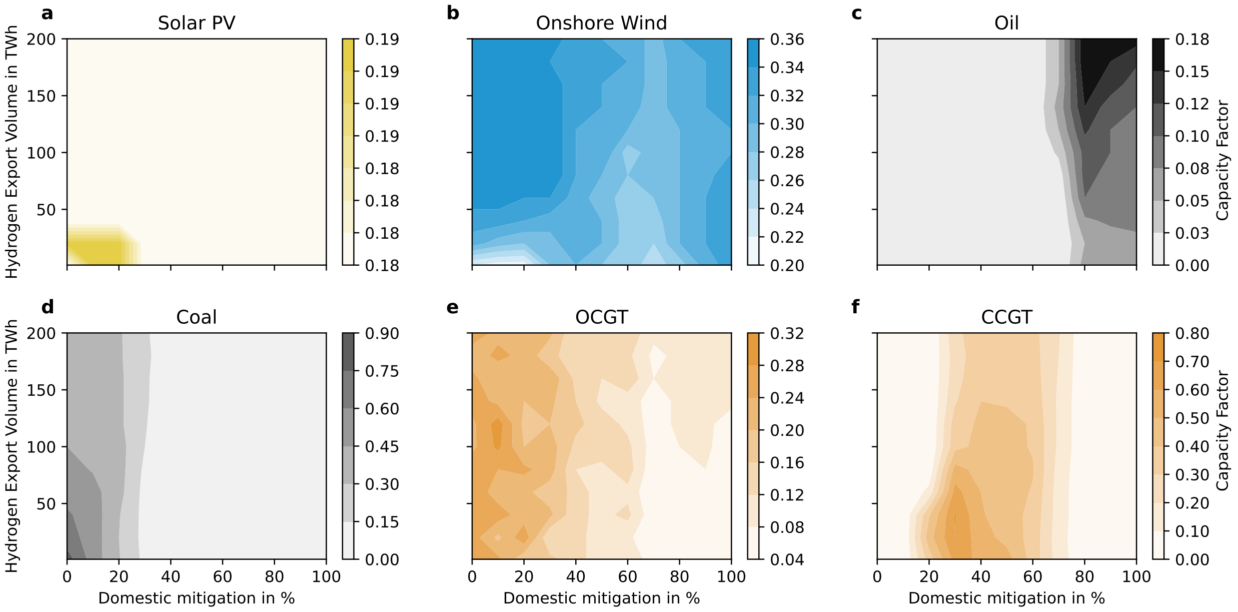

Supplementary Fig. 14: Electricity capacity factors with hourly temporal matching. Coal utilization (**d**) decreases as domestic CO_2 mitigation progresses, with combined cycle gas tubines (CCGT) acting as a substitute (**f**). In low hydrogen export scenarios, onshore wind (**b**) experiences high curtailment rates, whereas solar photovoltaics (PV) is constant (**a**). However, at higher export volumes, increased system flexibility reduces curtailment rates. The capacity factors of open cycle gas turbines (OCGT) (**e**) decrease while the oil generator capacity factors (**c**) increase up to 18%, but the total oil dispatch remains below 3 TWh.

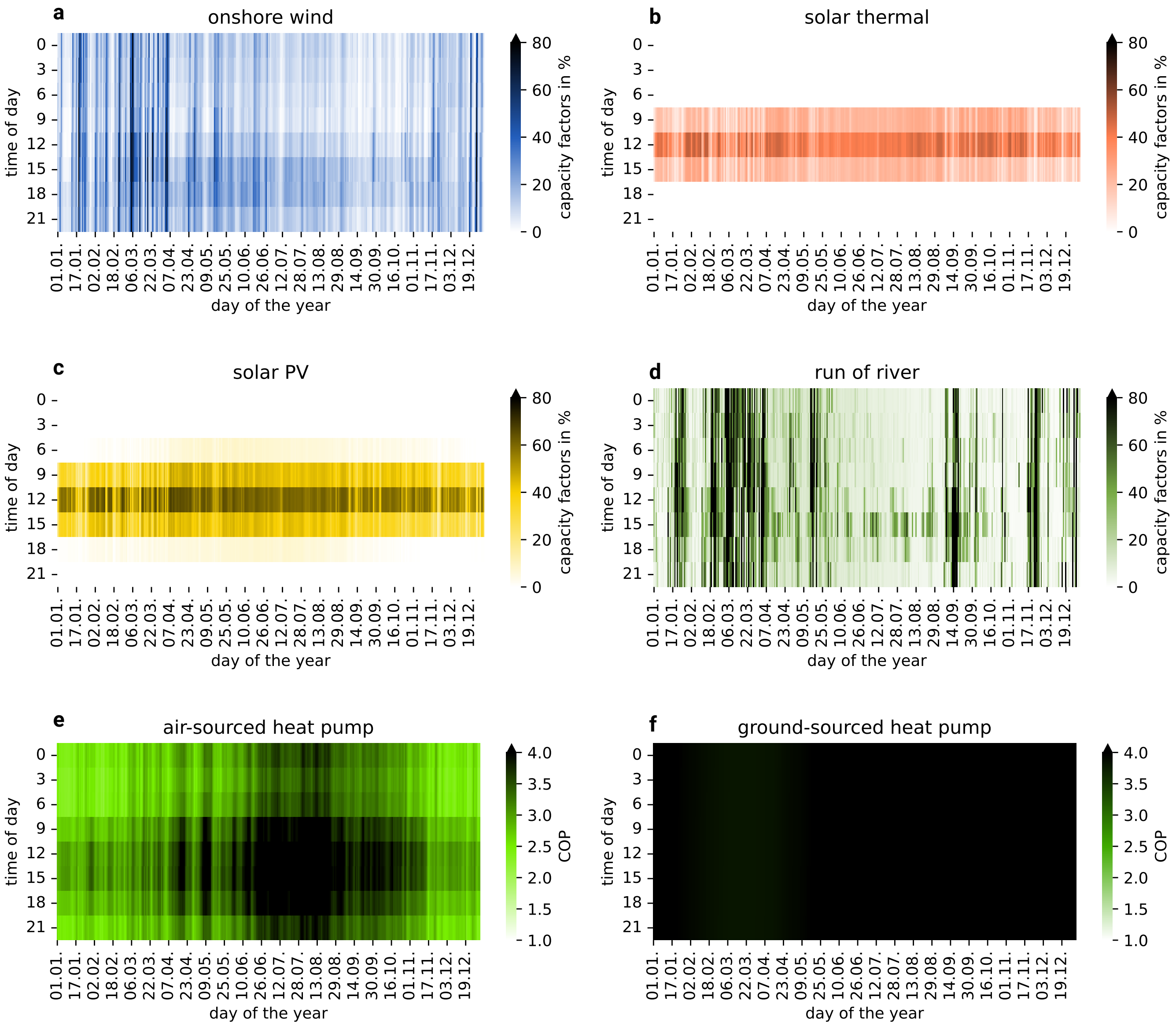

Supplementary Fig. 15: Capacity factors of renewable sources, and coefficient of performance (COP) of air-sourced and ground-sourced heat pumps. While solar thermal and solar photovoltaics (PV) show a diurnal pattern (**b**,**c**), onshore wind (**a**), run of river (**d**), and heat pumps (**e**,**f**) have a seasonal pattern.

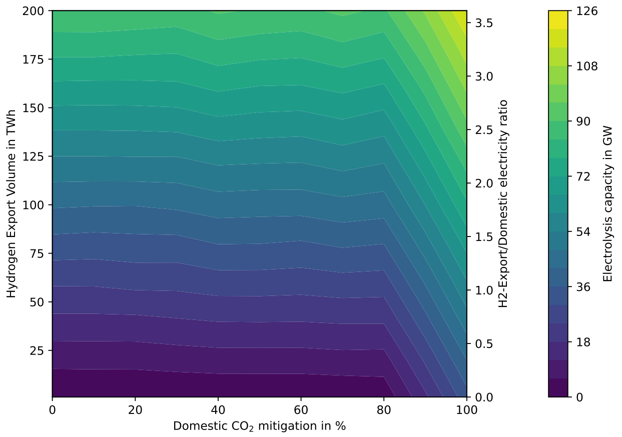

(a) Electrolysis optimal capacity

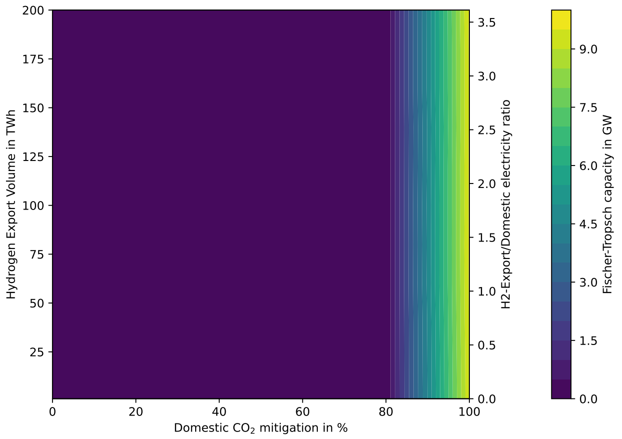

(b) Fischer-Tropsch optimal capacity

Supplementary Fig. 16: Electrolysis and Fischer-Tropsch capacities. Electrolysis capacity scales with hydrogen exports, but is also required for deep decarbonization scenarios above 80% of CO_2 mitigation. In those scenarios, Fischer-Tropsch capacities supply renewable fuels for the domestic demand.

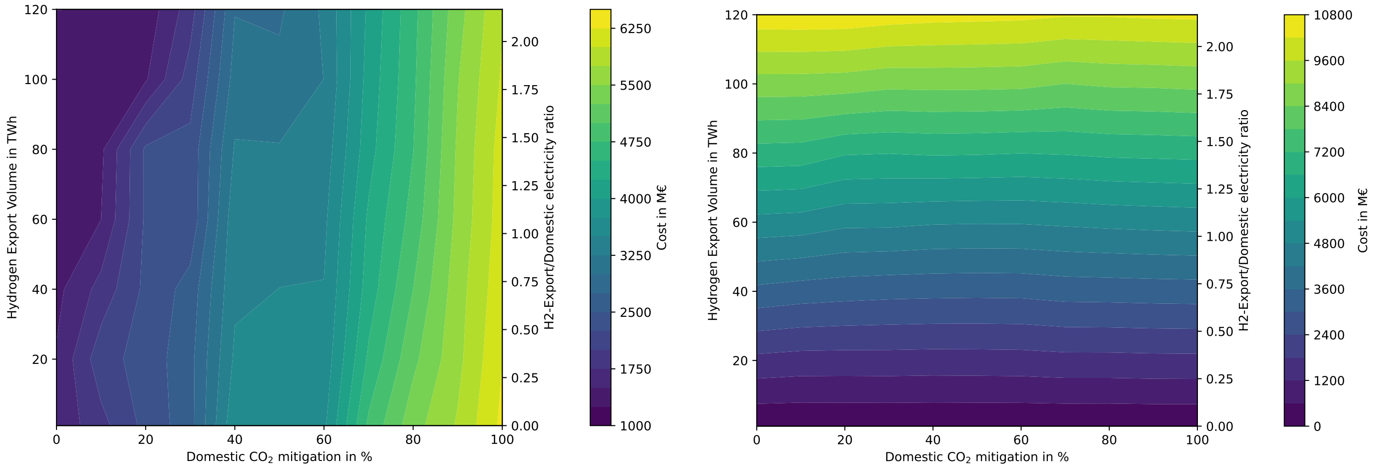

(a) Absolute cost of electricity for domestic consumers

(b) Absolute cost of hydrogen for exporters

Supplementary Fig. 17: Absolute, market-based costs for domestic electricity consumers (**a**) and hydrogen exporters (**b**). The absolute, market-based costs for domestic electricity consumers increases with domestic CO_2 mitigation, as higher electricity demand in deep decarbonization scenarios necessitates expanded electricity infrastructure. Similarly, the absolute cost for hydrogen exporters increases with hydrogen export volume, as larger exports require additional renewable generation, electrolysis capacity, and transport infrastructure.

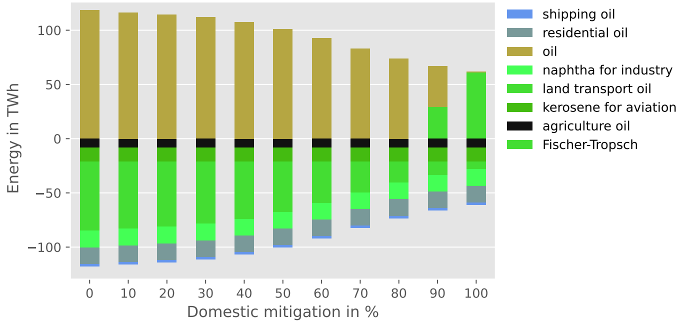

Supplementary Fig. 18: Oil balance at 1 TWh export at increasing domestic CO_2 mitigation. The exogenously defined oil demand for land transport substantially decreases due to electrification, whereas oil demands for industrial naphtha, aviation, agriculture are constant. The demand is met by fossil oil up to 80% CO_2 mitigation, then gradually replaced by Fischer-Tropsch fuels.

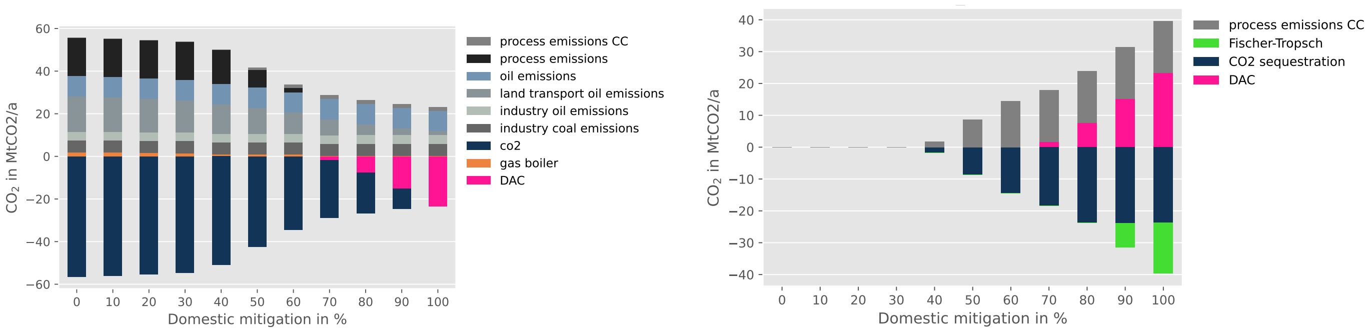

(a) CO_2 emissions and capturing of non-electricity sectors

(b) Balance of captured CO_2

Supplementary Fig. 19: CO_2 emissions and capturing (19a) as well as management of captured CO_2 (19b) with increasing emission limit at 1 TWh export volume. Deep decarbonization scenarios above 70% domestic CO_2 mitigation require direct air capture (DAC) to balance residual emissions. CC = carbon capture.

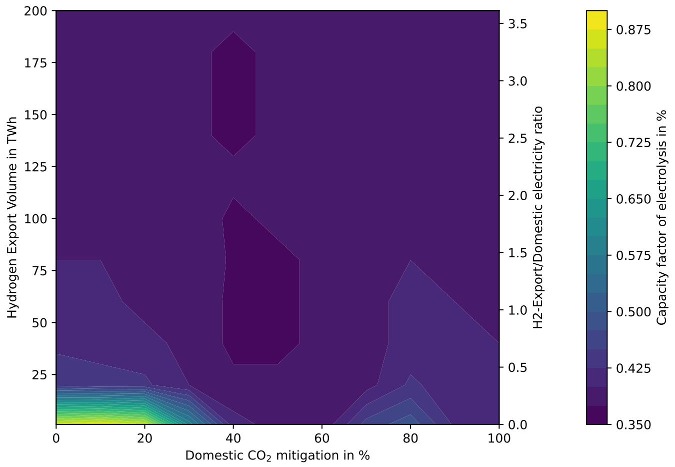

(a) Capacity factor of electrolysis

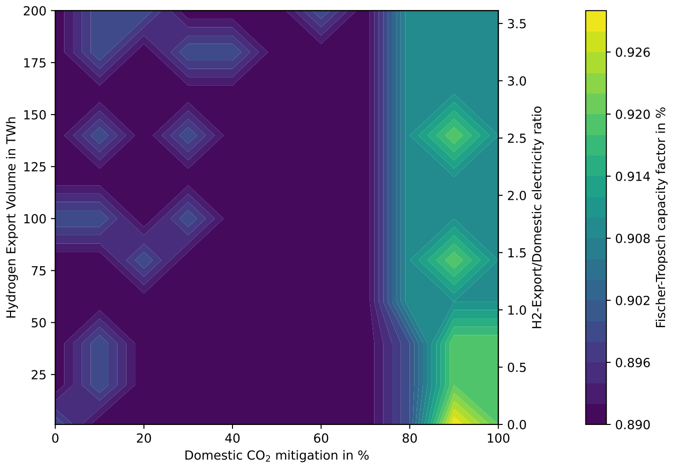

(b) Capacity factor of Fischer-Tropsch

Supplementary Fig. 20: Capacity factors of electrolysis and Fischer-Tropsch. The electrolysis has a high capacity factor in low export and low domestic CO_2 mitigation scenarios. The capacity factor for Fischer-Tropsch is close to 1 given the limited flexibility of the process.

Supplementary References

[1] Neumann, F., Zeyen, E., Victoria, M. & Brown, T. The Potential Role of a Hydrogen Network in Europe. *Joule* **7**, 1793–1817 (2023). URL 10.1016/j.joule.2023.06.016. 2207.05816.

[2] Abdel-Khalek, H. *et al.* PyPSA-Earth sector-coupled: A global open-source multi-energy system model showcased for hydrogen applications in countries of the Global South. *Applied Energy* **383**, 125316 (2025). URL https://www.sciencedirect.com/science/article/pii/S0306261925000467.

[3] Rim, B. *et al.* Morocco's Decarbonization Pathway - Part IV: Policy Recommmendations. Tech. Rep. (2021). URL https://www.policycenter.ma/sites/default/files/2022-11/PB-26-21-Enel-Green-Power-EGP-EN-PART%20IV0.pdf.
