# Supplementary material for: The impact of temporal hydrogen regulation on hydrogen exporters and their domestic energy transition
Source: Nat Commun. 2025 Aug 12;16:7486. doi: 10.1038/s41467-025-62873-w (PMC12343794; doi:10.1038/s41467-025-62873-w)
Supplement: Supplementary file 2 — Transparent Peer Review file [file 41467_2025_62873_MOESM2_ESM.pdf]

nature portfolio

Peer Review File

The impact of temporal hydrogen regulation on hydrogen exporters and their domestic energy transition

Corresponding Author: Mr Leon Schumm

This file contains all reviewer reports in order by version, followed by all author rebuttals in order by version.

Attachments originally included by the reviewers as part of their assessment can be found at the end of this file.

Version 0:

Reviewer comments:

Reviewer #1

(Remarks to the Author)
This study makes an important contribution to the emerging literature on hydrogen regulatory structures, exploring for the first time their potential impacts in a country with significant hydrogen export ambitions. The paper presents a compelling analysis with valuable and timely conclusions. I do not have much to critique regarding the analytical approach, but I believe the presentation and communication of results need to be improved before the paper can be accepted for publication. My comments to the authors are listed below:

- The colormap in Figure 4 is not very intuitive, particularly the way it normalizes to the bottom of each column (in 4a) or row (in 4b), rather than to a single point on each plot. While this is explained reasonably well in the caption, it is not the way that heatmaps are typically presented and could lead to reader confusion (e.g., why does electricity cost not change when moving from 0% to 100% mitigation in 4a). I would recommend either normalizing to a single point on each plot, e.g. 0% mitigation and 1 TWh exports, or breaking these out into individual columns/rows rather than leaving them as 2d fields if the authors prefer to keep the current normalization approach.

- The authors should be clear which cases are and are not subject to temporal regulation (e.g. in Figures 3 and 4). If these Figures are subject to hourly matching, they should include supplementary figures for the same scenarios with no rules. Figure S13 may be an example of this, but it is not explained and is never referenced in the main text.

- In general, the authors should ensure that supplementary figures are tied to the main text in some way.

- In the paragraph beginning on line 300 and in Figure 4, the use of 2030 and 2050 dates seems arbitrary and does not add explanatory value. By my reading this work uses a single-stage optimization and does not project to specific dates. The authors should consider simply using 'near-term' and 'long-term' or similar descriptors

- Figure 7 and the text accompanying it (lines 396-417) need to be better explained. Are these scenarios individual points along the line for pathway 2 in Figure 4? What does "(realistic)" mean in the figure? I understand what the figure is communicating at a high level but have no idea what specific scenarios are shown.

- Figure S14 is similarly confusing, but even moreso due to the additional unexplained scenarios. The panel titles also appear to be cut off.

- The difference in the impacts and importance of temporal regulation across the three pathways seems to be a central result. I would therefore suggest merging Figure S14 into Figure 7 so that all three pathways can be shown side by side. The figure still needs to be better explained, as discussed above.

- The study models scenarios where national decarbonization is achieved by what is effectively a cap-and-trade policy. In the limitations section, the authors should note that these results may not be fully applicable to other policy contexts, e.g. those where decarbonization is driven by subsidies or fixed carbon pricing

Additional minor comments:
- In line 123, should be "...such as low temporal..." rather than just "as"
- In Figure 3 (a), please adjust the y axis so that the bars do not hit the top of the frame
- In line 269, "this" rather than "is"

(Remarks on code availability)

Reviewer #2

(Remarks to the Author)
The reviewed manuscript analyzes the impact of transition pathways of hydrogen exporting regions on domestic and foreign stakeholders in the case of Morocco. The authors find that there are significant synergies between hydrogen export and local electricity cost , which increases the local domestic benefits of hydrogen exports. Based on this, three different development strategies of exporting countries are discussed and evaluated in terms of benefits for domestic and export costs, describing that quick exports lead to low electricity prices and balanced exports and mitigation measures lead to the lowest hydrogen costs. Temporal hydrogen regulation is another mechanism evaluated in this context showing that hourly regulation leads to lower electricity costs and emissions at the expense of hydrogen export costs.
This work presents a novel approach to evaluating expansion strategies for hydrogen exporting countries. It extends on current literature by providing a systematic overview for the evaluation of transition strategies depending on mitigation and export. In addition to the systematic overview, the impact of different temporal hydrogen regulations is considered. The manuscript is a valuable addition to the current literature. The study is structured in such way that the results not only provide new insights within energy system modeling but will also be of interest for all stakeholders of this field, such as governments, energy system designers, and various types of energy system participants.
The main design of the study is well thought out and therefore appropriate for the analysis conducted. The authors transparently are transparent about the limitations of their approach and draw conclusions only within their research design. While the main design is sound, the authors argumentation leads to some implausible conclusions due to an erroneous interpretation of Figure 4. This point is further elaborated in the section on major issues. Consequently, this part of the results, along with parts of the discussion, the conclusion, and the abstract require revision.
The method is well documented in accordance with the highest standards of the community. However, some details and context of the methodology setting are missing, which I have highlighted in the section on minor issues, and which should be resolved with small effort.

Major Issues:
• Line 288-313: As much as I like the analysis approach in these lines, I think this specific part of the analysis is not supported by the data and is misinterpreted from Figure 4. In general, Figure 4 has the following problem: In figure 4a) the z-axis is normalized to the x-axis, which means that only different values on the y-axis can be compared for ONE SPECIFIC x-value. Therefore, values at different x-values cannot be compared because they are normalized to different values. I like that you recognized this and point it out in line 242 ("displaying relative changes for domestic electricity consumers […] at a certain domestic climate change mitigation"). Figure 4b) has the same issue only with the normalization of the y-axis. Until line 287, all analysis points respect this limitation of the plot. From line 288 on, I see a violation of this limitation. For example: "A slight and short increase of domestic electricity prices in the early transformation phase (2030) is compensated by later (2050) strong (pathway 1) and medium(pathway 2) benefits for domestic electricity customers, profiting from hydrogen exports." (line301). There, values are compares along the pathway. This is not possible, as your defined normalization of the electricity and hydrogen costs changes along the pathway. If the absolute costs at (0%|1TWH) would be 20ct/kWh and 10ct/kWh at (15%|1TWH) your described "slight and short cost increase" would be a cost decrease, contradicting your analysis. Therefore, the section from line 288-313 is not evident (not necessarily wrong, just not evident). I would recommend visualizing Figure 4 in absolute terms, as this plot is prone to misinterpretation, as the author himself has done. Apart from the normalization, the figure itself is well thought-out and the reader learns a lot from it, but the definition of relative costs really reduces the interpretability of it.
• Line 443-499 in the discussion need to be revised with the updated results from the pathway analysis discussed above (Line 288-313). This is also true for parts of the conclusion and the abstract.

Minor issues:
• Line 1-21: there are no sources for your statements, please add
• Line 34: : https://doi.org/10.1016/j.ijhydene.2023.05.012 is another interesting source in this regard, as this paper present an approach to design local infrastructure for hydrogen export
Line 49: I found one really new studies in this gap: https://doi.org/10.1016/j.ijhydene.2024.04.153. Please also add this to the literature and re-elaborate the research gap (No worries, I still see a relevant research gap you close, but please update it with the newer source if applicable)
• Line 45: The paper https://doi.org/10.48550/arXiv.2407.07573 could be interesting to mention in this regard, as this paper presents a methodology for evaluating the impact of hydrogen export on social factors and local jobs
• Line 57: "point out benefits of … " on what? hydrogen cost, emissions, electricity costs..?
• Line 59 ff: If the source is worth mentioning, please also state their relevant outcome: "The effect of regulatory options on social welfare and carbon emissions is assessed in Brauer et al.", with the outcome of …
• Line 70: "Numerous studies focus on" but no citations. What does this exactly refer to? The stated studies above? Please clarify
• Line 79: "sector coupled" please specify which sector are coupled. Probably, electricity and hydrogen are meant, but in

theory various sectors could be coupled.
• Line 85: This overview is nice and helpful to understand the scope of analysis. Maybe this is a good point to explain the difference between "no regulation" and "annual" matching as raised in my comment for line 325
• Line 85: "Domestic climate change mitigation %" or "Domestic CO2 mitigation %"? That's a difference. Generally, you seem to use climate change mitigation and CO2 mitigation as a synonym, which they are not. Please explain or adapt.
• Line 111: Probably the data source for global administrative areas (GADM?) is missing
• Line 160: if the citation is meant for the whole paragraph, it should be behind the point
• Line 167: abbreviation LULUCF without explanation (Land Use, Land Use-Change and Forestry?)
Results:
• Figure 4: Please explain the definition of relative costs in the figure as well. The x label could be more specific like "Domestic CO2 mitigation %"
• Line 248: "electricity system profits from excess green electricity for H2 export". Why so? Is the curtailment decreased at higher export rates?
• Lien 263: "decreases the hydrogen export prices up to -9%." Its actual costs not prices as named in the figure 4.
• Line 263: "decreases the hydrogen export prices up to -9%." Why so? I think this point and the point from Line 248 are relevant for your conclusion in the following way: If the reason for both results is a universal property of exporting hydrogen energy systems under CO2 mitigation, then your conclusion in line 490 ("The three illustrative pathways offer certain room for manoeuvre in policy-making for various countries") is valid. If the reason for decreasing hydrogen cost just that this specific energy system situation comes from e.g. specifically cheap potentials in this area for Morocco, then the conclusion is only valid for Morocco. I think a short evaluation helps supporting your conclusions drawn.
• Line 271: failed reference probably to figure S10b)
• Line 325: "hourly basis": the model is at a resolution of 3 hours (line 97); how can the time matching be at an hourly basis?
• Line 325: Figure S16 implies that you optimized the operation of 1 year. If so, what is the difference between "no policy" and "annual"? for annual, can only green hydrogen be exported and for no policy, also hydrogen from brown coal can be exported? Please clarify
• Line 326: typo: "marginal cost OF fossil […]"
• Line 343: missing reference
• Figure 6: What is exactly the difference to Figure S9? S9 shows the total annual cost which are strictly increasing as the feasible space is further confined by temporal limitations. Until this point, I can follow your results. F6b) has decreasing "cost for consumer" with them being only part of the total annual costs. What are those cost exactly and why do they decrease? Do the consumers not have to pay the full total annual cost? Please clarify the difference.
• Line 388: something is missing. maybe "steers the redistribution OF CONSUMER COSTS between …"
• Line 392: "The energy spillover effect not only decarbonizes hydrogen exports…". I don't see how the "energy spillover" decarbonizes the hydrogen export. You defined the energy spillover as "provide excess electricity to the domestic electricity system" (line 327). So, when the green hydrogen production is limited by the temporal matching, excess green electricity is dispatched to the domestic grid reducing the dispatch of coal and therefore grid and total emissions. But by that definition energy spillover cannot impact the hydrogen emissions, as it only takes leftover energy to decarbonize local emissions, right? Also, with temporal matching of renewables to electrolysis, the exported hydrogen should not have any emissions at all. The introduction of temporal matching leads to a reduction of export hydrogen emissions as it introduces a policy for green hydrogen, but the spillover does not.
• Figure 7: I like that you chose a scale starting from 0 to show the actual deviation and therefore being unbiased.
• Line 401: "across the pathway": as far as I understood the model right, there is no pathway modelled. Do you mean the "annual optimized 2030 energy system designs across the pathway"? Because this somehow implies that you calculated a coherent pathway, which I think you did not.
Discussion:
• Line 521: On the other side exporting hydrogen is a chance to boost local economies as shown in https://doi.org/10.48550/arXiv.2407.07573
• Line 540: For the limitation, you should mention that you did not calculate transition pathways but instead approximated them by using myopic energy systems for 2030 and if there are limitations arising therefrom. Maybe a short comparison to https://doi.org/10.1016/j.ijhydene.2024.04.153 helps spotting methodology differences in myopic energy systems on a transition path vs. actual transition paths.
• Line 565: There is a publication which exactly quantifies the effect of storage sizes for LH2 export: https://doi.org/10.1016/j.ijhydene.2023.05.012 Figure 6 evaluates the cost share of hydrogen storages for export being below 5% cost for solar regions. Just as a further reference.
Methodology:
• Line 633: The setting of the model seems to unclear. Based on the methodology a transformation pathway is optimized? Is only on year optimized with the assumed CO2 reduction of 0-100% to today? If yes, which economic year and why? Is it 2030 because of line 715? What time span was optimized? As Figure S16 implies one year? Please define the general setting of the model (in methodology or the model section in the introduction). Also, a system overview (e.g. in the supplementary data) would be helpful, as you also described the impact of hydrogen demand on P2X for combustion engines demand. From the description within the manuscript, I would have never initially guessed, that this is modelled as well.
• Line 641: Maybe you can add a sentence about the demand of fuels, as this would explain the following sentence below Figure S9: "The large share of oil OPEX is independent of temporal hydrogen regulation, since these costs are mainly linked to combustion engine cars with demands independent of temporal hydrogen regulation."
• Line 677: Which weather year was used for the model?
• Line 678: "[…] of 36". I know the rule to not cite numbers directly but always add the name in front.
• Line 707: same as for Line 678
• Line 715: the link is wrong; the right one is probably: https://github.com/PyPSA/technology-data. But still, there are several

data set linked. Please cite the techno economic data clearly, as they are highly relevant for comparison and evaluation. Ideally, you are listing them directly in the paper or supplementary data to avoid confusion. I can only guess that the techno-economic data are coming from https://github.com/PyPSA/technology-data/blob/master/inputs/costs_PyPSA.csv

• Line 716: Currently, in the academic of global hydrogen the global interest rates are a highly discussed topic. Is this number "13%" just assumed or derived from a source. If second, please provide a proper citation.
• Technology is unclear: what kind of electrolysis are you using? PEM; SOFC, alkaline electrolysis? Please mention that
• Line 735 and 737: direct use of citation numbers in text

(Remarks on code availability)
The code is made available to a high degree. It includes a well-documented code package as well as installation guides and can therefore be reproduced. This is true for the code of the optimization model as well as the conducted result analysis. The only thing not being available are the input data as well as the result data.

Reviewer #3

(Remarks to the Author)
This paper seeks to investigate the interaction of domestic climate change mitigation targets, hydrogen export volumes, and carbon intensity regulation for hydrogen production in the context of Morocco using a sector-coupled energy system model. The authors implement a linear optimization of the generation, transmission, and storage capacities to minimize total annualized energy system costs, constrained by factors such as climate change targets, hydrogen export volumes, and the carbon intensity of electrolytic hydrogen production. The calculations quantify synergies between hydrogen exports and domestic climate change mitigation, such as reduced domestic emissions and lower total annualized system costs for power supply. The authors also argue that the hourly temporal matching of renewable power generation and electrolytic hydrogen production can serve as a tool to prevent domestic power generation system costs from rising.

While the topic is timely and relevant, my enthusiasm for this paper remains limited. This is primarily due to concerns about its methodology, exposition, contribution, and real-world insights. In particular, the methodology suffers from oversimplified economic assumptions that compromise the paper's usefulness and may lead to inaccurate conclusions. The following items elaborate on my concerns:

1. The key issue of the methodology is that the authors essentially treat the energy economy of a country as an engineering problem. That is, they take the perspective of a central planner and calculate the lowest cost required for the generated (and transmitted) energy to meet given demands under different constraints. But economies almost never work that way. They rely on markets and market participants making their own decisions. Regulators can set market requirements and incentivize market participants to pursue certain goals (e.g., emission reductions). The problem with the approach taken by the authors is that it fundamentally limits the contribution and real-world insights of the analysis. The approach can effectively only characterize particular relationships (e.g., between the export volume of electrolytically produced hydrogen, the emissions from power generation, and the total cost of the power generation system). Quantitative estimates of specific outcomes ignore the complexity of market economies and are, therefore, prone to error.

2. Given the approach taken, the authors should focus on characterizing relationships between the variables of interest. Instead, however, they primarily present their estimates of these variables without adequately explaining the mechanisms driving the estimates. As a result, the analysis offers limited real-world insights, restricting the implications to the specific calculations conducted by the authors.

3. In addition to the preceding methodological concerns, the methodology (i.e., the optimization problem and the solution process employed) is not clearly explained. The authors should formally introduce the model elements, discuss the decision and outcome variables, and clarify the assumptions for the decision variables. Without this information, the methodology lacks transparency.

4. It also appears that the total energy demand per sector is assumed to be constant. This assumption is not convincing since the energy demand per sector is usually affected by factors such as regulations and climate change mitigation targets. There is a substantial amount of research dedicated to estimating future energy demand as economies transition from fossil fuel to renewable electricity as the primary energy source.

5. As discussed above, the analysis in the paper ignores the complexities of market economies. One such complexity is the structure of the market (e.g., is the market highly regulated or liberalized?). This can substantially affect the extent to which changes in the costs of power generation lead to changes in consumer electricity prices. One finding of the paper is that under hourly temporal regulation, domestic electricity prices do not increase. At the same time, it remains completely unclear what market structure is assumed in the analysis, making it impossible to properly interpret the previous finding.

6. The analysis also implicitly assumes that increasing hydrogen exports and more ambitious mitigation targets will lead to higher installed renewable power capacity. But this is not necessarily the case. The authors should explore the factors influencing such investment decisions more thoroughly. They should also distinguish more clearly between short-term and long-term analysis. For example, the authors state that fossil fuel power generation has "higher marginal costs" compared to renewable energy sources. However, it remains unclear, whether the authors are referring to long-run or short-run marginal costs.

7. The study should also provide more information on technical aspects, such as the employed technologies, specifically which types of electrolyzers are used. This is important, as electrolyzers substantially differ in aspects such as cost or capacity factors and may need to be coupled with other electricity storage assets, impacting the cost of hydrogen production.

8. The concept of price spillover also requires further elaboration. Are fossil fuels entirely considered substitutes for renewables, or can they be complementary in some cases? Exploring this relationship is important because it affects how energy markets respond to price changes and policy regulations.

In addition to the preceding concerns, there are several minor issues:
• The paper seems to use the terms "cost" and "price" interchangeably, which is generally not correct.
• The writing style could be less belligerent, and there are several issues and typos (e.g., the authors write on p. 11: "Hourly matching (from 1.1.2030 onwards, until 31.12.2029 monthly)," which is logically incorrect).
• Some figures are not referenced correctly, and some GitHub links do not work properly (e.g., on pages 4, 6, and 10).
• The referencing style should be more consistent.

In sum, I cannot recommend this paper for publication in Nature Communications. If the authors seek to pursue this line of inquiry further, it appears indispensable that they reconsider the approach and exposition of the entire paper.

(Remarks on code availability)

Reviewer #4

(Remarks to the Author)
I co-reviewed this manuscript with one of the reviewers who provided the listed reports. This is part of the Nature Communications initiative to facilitate training in peer review and to provide appropriate recognition for Early Career Researchers who co-review manuscripts.

(Remarks on code availability)
I reviewed the code available on GitHub but did not perform a comprehensive analysis to rerun it and verify the reproducibility of the results. Our review primarily focused on the paper's content and its contributions rather than on reproducing the results using the code.

Version 1:

Reviewer comments:

Reviewer #1

(Remarks to the Author)
I commend the authors for comprehensively addressing my critiques and those of the other reviewers, and I am ready to recommend the paper for acceptance.

(Remarks on code availability)

Reviewer #2

(Remarks to the Author)
Most of my concerns were addressed appropriately, and the authors have added valuable background information, specifications, and refinements to the methodology description. Additionally, the inclusion of raw data provides a more detailed understanding of the work.
However, my main concern remains unresolved. As noted in my previous comments, Figure 4 is prone to misinterpretation, which previously led to incorrect conclusions being drawn, as acknowledged in the first revision. While the problematic conclusions have been adequately removed and the integration of transformation pathways has improved, Figure 4 itself has not changed. Reviewer #1 also highlighted that the chosen visualization does not effectively represent the data, as the normalization approach appears unintuitive and misaligned with the plot type. Even if one understands its limitations, it is not entirely clear what interpretations the figure allows, and what might be misinterpretations.

After further consideration, I think I understand why you chose this approach to normalization and the corresponding plots. For instance, in Figure 4(a), are you aiming to show the effect of external stakeholder interests—specifically hydrogen exports—on the local benefits of electricity costs? If so, it would make sense to examine the partial derivative of electricity costs with respect to hydrogen exports while holding CO_2 mitigation constant as you did. Similarly, for Figure 4(b), are you illustrating how internal stakeholder interests in CO_2 reduction result in benefits for external stakeholders through reduced hydrogen export costs? If this is your intention, then the plot becomes more logical.
This storyline, however, does not come across clearly in the current manuscript. I recommend revising lines 139–147 and 148–165 to emphasize this narrative. With these updates, your analysis would better support the adapted conclusions presented in line 166 and beyond.
Additionally, to help readers avoid misinterpreting the figure, I suggest incorporating visual aids to guide interpretation. As

this is hard to describe, I have included a suggestion in the attached files (Suggestion_Figure_4.png) to illustrate what would have helped me interpret the figure correctly from the outset.

If my interpretation of Figure 4 does not align with your intent, this indicates residual ambiguity that should be even more addressed before publication.

(Remarks on code availability)
The code is made available to a high degree. It includes a well-documented code package as well as installation guides and can therefore be reproduced. This is true for the code of the optimization model as well as the conducted result analysis. Also the input data as well as the result data are available.

Reviewer #3

(Remarks to the Author)
The authors have substantially revised the manuscript and addressed some of my earlier comments or argued why doing so is not feasible within the scope of their analysis. However, several core concerns remain.

1) As pointed out before, the analysis takes the perspective of a central planner who effectively makes all decisions in the model economy. The authors argue that this approach is common in energy system modeling and can provide some real-world insights, even if it is not reflective of most market economies. I agree with this. However, the authors also state that they assume a liberalized market. This contradicts the modeling approach! Either the analysis assumes a central planner seeking to minimize the total cost of the energy system to meet a given energy demand. Or the analysis assumes a liberalized market in which individual market participants maximize their profits in equilibrium. Both approaches require specific assumptions. Importantly, the results of the two modeling approaches are often fundamentally different!

2) In response to point 5 of my earlier comments, the authors write: “low marginal price excess electricity from these [renewable] sources penetrates the market, reducing prices for domestic consumers. This dynamic does not rely on the specific market structure alone but rather on the availability of competitive renewable electricity that influences marginal prices. […] [E]ven in a more regulated market with a vertically integrated utility, this mechanism would hold. […] Thus, the relationship between hourly regulation and consumer electricity prices is relatively independent of the market design.”

Again, the authors confuse economic concepts. High renewable power generation in some hours suppresses prices in the wholesale electricity market in those hours. But whether this temporary price suppression in the wholesale market results in lower electricity prices for residential consumers depends on many factors, including the market structure, market regulation, retail electricity tariffs, and wholesale market prices during hours of low renewable power generation. So-called “hourly regulation” may lead to more renewable energy deployment and thus to more frequent and larger suppressions of wholesale market prices. Yet, the relationship between this hourly regulation and consumer electricity prices is generally not independent of the market design.

This issue is crucial because the author’s assumptions about the market design remain unclear. At the same time, the main results of the paper concern the relationship between this hourly regulation and consumer electricity prices.

3) While the authors now better explain some mechanisms, they remain silent on others. For example, Figure 4a shows that the output variable “Relative domestic electricity cost” is strongly non-monotonic in the two input variables plotted on the x- and y-axes of the graph. Similarly, the output variable “Relative export hydrogen cost” in Figure 4b appears to be generally non-monotonic in the input variables plotted on the x- and y-axes of the graph. Both graphs beg the question: why? What’s going on here?

In addition to the preceding concerns, there are several minor issues:
• l. 58: “drices” should read “drives”
• l. 216: I would suggest deleting the part “leading the system closer to an equilibrium where marginal prices match the optimization’s objective function” because it is unclear what is meant in economic terms.
• Figure 7 shows a line chart for findings that change by category. This depiction is highly misleading as it suggests that the linear interpolations between the categories are actual results. I would strongly suggest providing some other depiction of the findings that avoid this issue.

Overall, I still cannot recommend this paper for publication in Nature Communications. It remains essential that the authors reconsider and clean up the economic approach and assumptions made in the analysis.

(Remarks on code availability)

Reviewer #4

(Remarks to the Author)
I co-reviewed this manuscript with one of the reviewers who provided the listed reports. This is part of the Nature

Communications initiative to facilitate training in peer review and to provide appropriate recognition for Early Career Researchers who co-review manuscripts.

(Remarks on code availability)

Version 2:

Reviewer comments:

Reviewer #2

(Remarks to the Author)
The authors have successfully addressed my final remarks. The paper now makes a valuable contribution to the scientific community by introducing a novel approach to evaluating export energy systems. I have no further comments.

(Remarks on code availability)

Reviewer #3

(Remarks to the Author)
The authors have further revised the manuscript and have addressed most of my earlier concerns. Yet, several items require further clarification.

1. In response to earlier comments, the authors have clarified that their analysis takes the perspective of a central planner. To further avoid confusion, I would suggest deleting the following two sentences:
a. l. 96 ff.: “The model assumes a central planner’s perspective, with the results being equivalent to a decentral market solution under the conditions outlined in the section Discussion.”
b. l. 366 f.: delete the part “based on economic theory, where market outcomes are derived under the assumption of complete and competitive markets.”

2. In the paper, the authors generally speak of the “costs for domestic electricity consumers.” Hereby, they seem to mean the total cost of the electricity system, not the electricity bills consumers pay. The former cost metric follows directly from the analysis. The latter cost metric would require further assumptions, for instance, about the market structure and the cost pass-through from utilities to end consumers. Since this can be easily misunderstood, I would ask the authors to clarify the language, possibly by avoiding the term “costs for domestic electricity consumers.”

3. The manuscript would also benefit from a more precise framing regarding whether the analysis is fundamentally static or dynamic. Some parts emphasize the level of mitigation and export (i.e., scenario endpoints), while others refer to the speed of transition. Since the core of the modeling approach appears to be static (optimization for 2030), such temporal references and discussions should be more carefully explained.

(Remarks on code availability)

Reviewer #4

(Remarks to the Author)
I co-reviewed this manuscript with one of the reviewers who provided the listed reports. This is part of the Nature Communications initiative to facilitate training in peer review and to provide appropriate recognition for Early Career Researchers who co-review manuscripts.

(Remarks on code availability)

Open Access This Peer Review File is licensed under a Creative Commons Attribution 4.0 International License, which permits use, sharing, adaptation, distribution and reproduction in any medium or format, as long as you give appropriate credit to the original author(s) and the source, provide a link to the Creative Commons license, and indicate if changes were made.
In cases where reviewers are anonymous, credit should be given to 'Anonymous Referee' and the source.
The images or other third party material in this Peer Review File are included in the article’s Creative Commons license, unless indicated otherwise in a credit line to the material. If material is not included in the article’s Creative Commons license and your intended use is not permitted by statutory regulation or exceeds the permitted use, you will need to obtain permission directly from the copyright holder.
To view a copy of this license, visit https://creativecommons.org/licenses/by/4.0/

Author Response to Reviews of

The impact of temporal hydrogen regulation on hydrogen exporters and their domestic energy transition

Leon Schumm, Hazem Abdel-Khalek, Tom Brown, Falko Ueckerdt, Michael Sterner, Davide Fioriti, Max Parzen
Nature Communications
Revision #1: November 13, 2024

RC: Reviewer Comment, AR: Author Response, ▌ *Manuscript text*

We thank the reviewers for their constructive comments and acknowledge the time and effort they have spent in assessing our work. We have revised the paper based on the feedback from the reviewers and hope that we can adequately address their primary concerns.

To follow the revisions made, we have highlighted differences compared with the previous submitted version of the paper in blue and red text in an attached file. Figure numbers in this response letter refer to the numbering in the revised version of the manuscript.

Reviewer #1

RC: The colormap in Figure 4 is not very intuitive, particularly the way it normalizes to the bottom of each column (in 4a) or row (in 4b), rather than to a single point on each plot. While this is explained reasonably well in the caption, it is not the way that heatmaps are typically presented and could lead to reader confusion (e.g., why does electricity cost not change when moving from 0% to 100% mitigation in 4a). I would recommend either normalizing to a single point on each plot, e.g. 0% mitigation and 1 TWh exports, or breaking these out into individual columns/rows rather than leaving them as 2d fields if the authors prefer to keep the current normalization approach.

AR: We are thankful for the comment, and understand that Figure 4 requires improvement. While we thankfully acknowledge the suggestion to either normalize to a single point or breaking these out into individual columns/rows, we would like to contribute to the discussion of those suggestions.

If we refrain from the normalization and show absolute costs, we indeed get first order insights: both scenario variables (hydrogen export quantity and domestic CO_2 mitigation) increase the costs as observable in Figure S8, which we have added to the supplementary material. This is an interesting, yet not novel, insight. Unfortunately, the increasing costs dominate both figures and

therefore conceal the impacts on hydrogen exporters and domestic electricity consumers.

However, to analyze how hydrogen exports impacts domestic electricity consumers or how domestic CO_2 mitigation affects hydrogen exporters, we have chosen the normalization approach revealing those second order insights. Even more so, those insights are no longer just second order but highly relevant to hydrogen exporters and domestic electricity consumers. This way, we can clearly isolate the effects of hydrogen exports on domestic consumers (Figure 4a) and domestic CO_2 mitigation on hydrogen exporters (Figure 4b). Without normalization, or a normalization to one specific point (e.g. 0% CO_2 mitigation and 1 TWh/a export), these effects are not outlined in a satisfactory manner.

Nonetheless, we fully agree with the reviewer, that the normalization approach applied here might lead to reader confusion. To address this, we have taken several measures.

First, we have corrected the previously misleading statement in line 239 "First, Figure 4a shows the cost for domestic electricity consumers subject to mitigation and hydrogen export volumes", regarding the fact that in Figure 4a, the cost for domestic electricity consumers is indeed not subject to mitigation but hydrogen export volumes only in line 139:

> *First, Figure 4a shows the cost for domestic electricity consumers subject to hydrogen export volumes.*

The similar misleading statement on Figure 4b in line 256 "Second, Figure 4b shows the cost of hydrogen exports subject to mitigation and hydrogen export volumes" is corrected in line 148:

> *Second, Figure 4b shows the cost of hydrogen exports subject to domestic CO_2 mitigation.*

Second, to further reduce the possibility of reader confusion, we have decided to elaborate in detail how the normalization is carried out, and how the figures should be interpreted ("vertical interpretation" for Figure 4a and "horizontal interpretation" for Figure 4b). This is carried out in the description of Figure 4:

> *Figure 4a shows the effect of hydrogen exports on domestic electricity cost. Therefore, the effect of hydrogen exports is isolated by normalizing the costs to 1 TWh/a hydrogen export in each column. This approach allows for a vertical interpretation in Figure 4a only, displaying the effects of hydrogen exports (vertical) at a certain domestic CO_2 mitigation level. Figure 4b shows the effect of domestic CO_2 mitigation on hydrogen export cost. Therefore, the effect of domestic CO_2 mitigation is isolated by normalizing the costs to 0% CO_2 mitigation in each row. This approach allows for a horizontal interpretation in Figure 4b only, displaying the effects of domestic CO_2 mitigation (horizontal) at a certain hydrogen export quantity.*

Third, we have added and integrated both Figures 4a and 4b in a non-normalized, absolute manner. We refer to it in the description of Figure 4:

> *See Figure S8 for absolute cost for domestic electricity consumers and hydrogen exporters.*

Forth, we have removed the pathway analysis in the contour plots of Figure 4a and 4b as well it's description. We fully recognize, that the conducted pathway analysis is not evident from the normalized data representation and might contribute to reader confusion.

To sum up, we fully recognize the reviewers criticism and have undertaken efforts to reduce possibility of reader confusion. While we feel that the normalization approach is indeed unusual but highlights the effects on hydrogen exporters and domestic consumers, we are open to use the non-normalized figure, breaking these out into individual columns/rows, or normalize to a single point instead, if the reviewer is in favour of those options.

RC: The authors should be clear which cases are and are not subject to temporal regulation (e.g. in Figures 3 and 4). If these Figures are subject to hourly matching, they should include supplementary figures for the same scenarios with no rules. Figure S13 may be an example of this, but it is not explained and is never referenced in the main text.

AR: We are thankful for the suggestion to add the counterparts without temporal regulation and clarify which cases are subject to temporal regulation. Indeed, both Figures 3 and 4 are subject to hourly temporal regulation, which we now refer to in the main text (description of Figure 3):

> *Electricity supply and demand at fixed export levels and increasing domestic CO_2 mitigation (3a) and vice versa (3b) with hourly temporal hydrogen regulation.*

and in the figure description of Figure 4:

> *Figure 4a shows the effect of hydrogen exports on domestic electricity cost with hourly hydrogen regulation. [. . .] Figure 4b shows the effect of domestic CO_2 mitigation on hydrogen export cost with hourly hydrogen regulation.*

In addition, we have added the counterparts without temporal matching in the Supplementary section "Electricity supply and demand without temporal hydrogen regulation" (counterpart for Figure 3) and "Cost for domestic electricity consumers and hydrogen exporters without hydrogen regulation" (counterpart for Figure 4). In addition, we reference to the case without temporal matching in the main text in line 126:

> *[. . .] is covered by coal power plants and CCGT (by increasing the capacity factor of existing brownfield coal and CCGT capacities) and an expansion of open-cycle gas turbines (OCGT) installation and supply as pointed out in Figure S10.*

Furthermore, the no-regulation counterpart of Figure 3 is referenced in the description of Figure 3:

> *See Figure S10 for electricity supply and demand if no hydrogen regulation is in place.*

and the no-regulation counterpart of Figure 4 is referenced in the description of Figure 4:

> *[. . .] see [. . .] Figure S12 for the interplay of hydrogen exports and domestic CO_2 mitigation if no hydrogen regulation is in place.*

RC: In general, the authors should ensure that supplementary figures are tied to the main text in some way.

AR: We acknowledge the suggestion to tie the supplementary figures to the main text. We have double-checked every supplementary Figure, both Figures S2 and S9 of the revised manuscript have already been tied. For all other Figures (including Figures added to the revised manuscript), we have tiem them to the main text.

Figure S1 in line 373:

> *A more detailed mathematical problem formulation is provided in Section Mathematical Model Formulation, and a system overview is provided in Figure S1.*

Figure S3, S4 and S5 in line 142:

> *In these ranges, the domestic electricity consumers profit from excess green electricity originating from additional renewable energy capacities required for hydrogen export, as pointed out in the overview on electricity supply, capacities and capacity factors in Figures S3, S4 and S5.*

Figure S6 in line 408:

> *Atlite obtains technology-specific time series of the weather year 2013 based on the ERA5 reanalysis data [69], displayed in Figure S6.*

Figure S7 in line 118:

> *Both electrolysers (s. Fig. S7) and BEVs drive the electricity demand substantially, up to two times of the domestic end-use electricity demand.*

Figure S8 and S12, S17 in the figure description of Figure 4:

> *See Figure S8 for absolute cost for domestic electricity consumers and hydrogen exporters, Figure S12 for the interplay of hydrogen exports and domestic CO_2 mitigation if no hydrogen regulation is in place and Figure S17 for hydrogen exports up to 200 TWh/a.*

Figure S10 and S16 in the figure description of Figure 3:

> *See Figure S10 for electricity supply and demand if no hydrogen regulation is in place and Figure S16 for hydrogen exports up to 200 TWh/a.*

Figure S13 and S14 in line 204:

> *See Figure S13 and Figure S14 for cost breakdown of hydrogen electrolysis with and without hourly temporal matching.*

Figure S15 in line 226:

> *They consist of (i) Quick exports and slow CO2 mitigation, (ii) Balanced exports and CO2 mitigation and (iii) Slow exports and quick CO2 mitigation as further elaborated in Figure S15a.*

Figure S18 in line 234:

> *This effect is even more pronounced at even higher export quantities, as observable in the sensitivity analysis carried out in Figure S18.*

Figure S19 in line 396:

> *[. . .], including the energy carriers electricity, hydrogen, natural gas, biomass, oil, and heat, as well as carbon dioxide in the form of emissions and feed-stock for the synthesis of other carriers (s. Fig. S19).*

Figure S20 in line 306:

> *[. . .] provides an additional flexibility to a certain extent but is limited by the operation flexibility of Fischer-Tropsch or Haber-Bosch processes (s. Fig. S20).*

RC: In the paragraph beginning on line 300 and in Figure 4, the use of 2030 and 2050 dates seems arbitrary and does not add explanatory value. By my reading this work uses a single-stage optimization and does not project to specific dates. The authors should consider simply using 'near-term' and 'long-term' or similar descriptors

AR: We confirm, that the study uses a single-stage optimization. We are thankful for the suggestion to use other descriptors. However, based on other reviewer comments rightfully criticizing the pathway analysis, which is not evident (not wrong, just not evident) from the normalization approach taken, we have decided to remove the pathway analysis in Figure 4 including the arrows with the descriptors "2030" and "2050" as well as the pathway analysis beginning on line 300 all together. Instead, we now outline various pathways in a fully revised Figure 7 (to which the pathway analysis is indeed applicable) and refrained from the arbitrary wording "2030" and "2050".

RC: Figure 7 and the text accompanying it (lines 396-417) need to be better explained. Are these scenarios individual points along the line for pathway 2 in Figure 4? What does "(realistic)" mean in the figure? I understand what the figure is communicating at a high level but have no idea what specific scenarios are shown.

AR: We are thankful for the remarks on Figure 7, specifically in regards to the scenario selection and the suggestion in a different comment ("I would therefore suggest merging Figure S14 into Figure 7 so that all three pathways can be shown side by side."). In addition, we agree that Figure S14 did not improve clarity but instead raised additional questions.

We have decided to fully address all raised concerns in a combined manner, and have i) completely revised Figure 7, taking all scenarios and pathways into account, ii) merged Figure S14 into Figure 7, iii) added a sensitivity analysis of high export scenarios on the Figure 7 in the appendix and iv) revised scenario description and the figure explaination in line 223:

> *Based on the effects of temporal hydrogen regulation in the scenario of 120 TWh/a export and 0% CO_2 mitigation presented in the previous section, this section explores the temporal hydrogen regulation across all mitigation (0–100%) and export (0-120 TWh/a) scenarios. We have grouped these scenarios into three illustrative mitigation-export pathways presenting various speeds of transformation. They consist of (i) Quick exports and slow CO_2 mitigation, (ii) Balanced exports and CO2 mitigation and (iii) Slow exports and quick CO_2 mitigation as further elaborated in Figure S15a.*
>
> *Figure 7 shows the relative change of electricity and hydrogen cost of all mitigation-export scenarios (grouped by speed of transformation) in dependence of temporal matching. The effects of temporal hydrogen regulation on domestic electricity prices are most dominant in the group Quick exports and slow CO_2 mitigation. In this group, the introduction of annual matching has already a striking effect, because the price spillover observed in Figure 5 decreases (domestic) electricity prices. The price spillover unfolds in fossil-dominated (hence low domestic CO_2 mitigation) systems, combined with high hydrogen exports providing a strong energy spillover effect. In the most extreme cases, characterized by high exports and no domestic CO_2 mitigation, the cost of electricity for domestic consumers reduces up to -55%. This effect is even more pronounced at even higher export quantities, as observable in the sensitivity analysis carried out in Figure S18.*
>
> *In the group of Balanced exports and CO_2 mitigation the effect of temporal hydrogen regulation is less dominant. In only two scenarios, hourly matching regulation increases the hydrogen cost above 7% (above 11% across all groups) compared to no temporal hydrogen regulation. This is the case for 0% CO_2 mitigation and 1 and 20 TWh/a export scenarios. However, even in the Balanced exports and CO_2 mitigation group the benefit of temporal hydrogen regulation for domestic consumers is substantial, reducing their cost of electricity up to -31%. In only two mitigation-export scenarios the annual or monthly matching decreases the cost for domestic electricity, whereas all other combinations of the Balanced exports and CO_2 mitigation group are only sensitive to the strictest – hourly matching – hydrogen regulation.*
>
> *In the slow exports and quick CO_2 mitigation group, only the hourly matching has observable effects, since the electricity system is already dominated by renewable electricity and only hourly matching phases out the remaining fossil generation. Therefore, the impacts on the cost for domestic electricity consumers (-11%) and hydrogen exporters (8%) are moderate.*
>
> *Figure 7 shows, that temporal hydrogen regulation hedges domestic electricity consumers against rising prices across all scenario groups. Putting the extreme scenarios of the Quick exports and slow CO_2 mitigation group aside (based on the assumption that export quantities above 80 TWh without domestic CO_2 mitigation are unlikely), hourly matching decreases the cost of electricity for domestic consumers by up to 31%. The cost for hydrogen exporters increase only slightly in the vast majority of all mitigation-export scenarios.*

RC: Figure S14 is similarly confusing, but even moreso due to the additional unexplained scenarios. The panel titles also appear to be cut off.

AR: We agree with the comment, and have fully revised Figure S14 and Figure 7 as well as their

explaination as outlined in the comment above ("Figure 7 and the text accompanying it (lines 396-417) [...]").

RC: The difference in the impacts and importance of temporal regulation across the three pathways seems to be a central result. I would therefore suggest merging Figure S14 into Figure 7 so that all three pathways can be shown side by side. The figure still needs to be better explained, as discussed above.

AR: We agree with the comment and are thankful for the suggestion. We have fully revised Figure S14 and Figure 7 as well as their explaination as outlined in the comment above ("Figure 7 and the text accompanying it (lines 396-417) [...]").

RC: The study models scenarios where national decarbonization is achieved by what is effectively a cap-and-trade policy. In the limitations section, the authors should note that these results may not be fully applicable to other policy contexts, e.g. those where decarbonization is driven by subsidies or fixed carbon pricing

AR: We thank the reviewer for this insightful suggestion. We have added a note in the limitations section to clarify the potential applicability of our results to other policy contexts in line 317:

> *Furthermore, this study achieves national decarbonization under what is effectively a cap-and-trade framework. As such, results may not fully generalize to other policy contexts, such as those driven by subsidies or fixed carbon pricing, which may influence decarbonization dynamics differently.*

RC: In line 123, should be "...such as low temporal..." rather than just "as"

AR: We agree and adjust in line 86:

> *Shortcomings of studies on highly renewable energy systems in Africa such as low temporal and spatial resolutions as well as the lack of sector-coupled energy models [...]*

RC: In Figure 3 (a), please adjust the y axis so that the bars do not hit the top of the frame

AR: Approved. We agree on the importance to clearly visualize the bar including a margin. We have discovered, that the same issue applies not just in Figure 3 (a), but also for Figure S12 (a) and S15 (a) and have adjusted all three graphs accordingly.

RC: In line 269, "this" rather than "is"

AR: We agree and adjust in line 135:

> *In an integrated analysis of domestic CO_2 mitigation (dimension 1) and hydrogen exports (dimension 2) this study outlines [...]*

Reviewer #2

RC: Line 288-313: As much as I like the analysis approach in these lines, I think this specific part of the analysis is not supported by the data and is misinterpreted from Figure 4. In general, Figure 4 has the following problem: In figure 4a) the z-axis is normalized to the x-axis, which means that only different values on the y-axis can be compared for ONE SPECIFIC x-value. Therefore, values at different x-values cannot be compared because they are normalized to different values. I like that you recognized this and point it out in line 242 ("displaying relative changes for domestic electricity consumers [. . .] at a certain domestic climate change mitigation"). Figure 4b) has the same issue only with the normalization of the y-axis. Until line 287, all analysis points respect this limitation of the plot. From line 288 on, I see a violation of this limitation. For example: "A slight and short increase of domestic electricity prices in the early transformation phase (2030) is compensated by later (2050) strong (pathway 1) and medium(pathway 2) benefits for domestic electricity customers, profiting from hydrogen exports." (line301). There, values are compares along the pathway. This is not possible, as your defined normalization of the electricity and hydrogen costs changes along the pathway. If the absolute costs at (0%|1TWH) would be 20ct/kWh and 10ct/kWh at (15%|1TWH) your described "slight and short cost increase" would be a cost decrease, contradicting your analysis. Therefore, the section from line 288-313 is not evident (not necessarily wrong, just not evident). I would recommend visualizing Figure 4 in absolute terms, as this plot is prone to misinterpretation, as the author himself has done. Apart from the normalization, the figure itself is well thought-out and the reader learns a lot from it, but the definition of relative costs really reduces the interpretability of it.

AR: We are thankful for the comment, and fully agree with the reviewer. Indeed, the selected normalization approach does not support the pathway analysis carried out, nor are the pathway arrows in Figure 4 applicable. We have removed the pathway analysis in lines 292-313. Regarding lines 288-291, we indeed see that in the range of medium mitigation (40–60%) and moderate to high hydrogen exports (50–100 TWh), both groups (domestic and export) profit from each other and therefore show a co-benefit. We have clarified in lines 168:

> *Hence, both domestic CO_2 mitigation and hydrogen exports have cost-reducing effects on hydrogen exporters and domestic electricity consumers respectively. They benefit from each other (co-benefit) at medium mitigation (40–60%) and moderate to high hydrogen exports (50–100 TWh).*

However, if the reviewer sees that this statement is not supported by the data of Figure 4, we are open to fully revise or remove this conclusion on co-benefits.

While we are thankful for the suggestion to refrain from the normalization but instead visualize it in absolute terms, we would like to contribute to the discussion of this suggestion.

If we refrain from the normalization and show absolute costs, we indeed get first order insights: both scenario variables (hydrogen export quantity and domestic CO_2 mitigation) increase the costs as observable in Figure S8, which we have added to the supplementary material. This is an

interesting, yet not novel, insight. Unfortunately, the increasing costs dominate both figures and therefore conceal the impacts on hydrogen exporters and domestic electricity consumers.

However, to analyze how hydrogen exports impacts domestic electricity consumers or how domestic CO_2 mitigation affects hydrogen exporters, we have chosen the normalization approach revealing those second order insights. Even more so, those insights are no longer just second order but highly relevant to hydrogen exporters and domestic electricity consumers. This way, we can clearly isolate the effects of hydrogen exports on domestic consumers (Figure 4a) and domestic CO_2 mitigation on hydrogen exporters (Figure 4b). Without normalization, or a normalization to one specific point (e.g. 0% CO_2 mitigation and 1 TWh/a export), these effects are not outlined in a satisfactory manner.

Nonetheless, we fully agree with the reviewer, that the normalization approach applied here might lead to reader confusion or misinterpretation. To address this, we have taken several measures.

First, we have corrected the previously misleading statement in line 239 "First, Figure 4a shows the cost for domestic electricity consumers subject to mitigation and hydrogen export volumes", regarding the fact that in Figure 4a, the cost for domestic electricity consumers is indeed not subject to mitigation but hydrogen export volumes only in line 139:

> *First, Figure 4a shows the cost for domestic electricity consumers subject to hydrogen export volumes.*

The similar misleading statement on Figure 4b in line 256 "Second, Figure 4b shows the cost of hydrogen exports subject to mitigation and hydrogen export volumes" is corrected in line 148:

> *Second, Figure 4b shows the cost of hydrogen exports subject to domestic CO_2 mitigation.*

Second, to further reduce the possibility of reader confusion, we have decided to elaborate in detail how the normalization is carried out, and how the figures should be interpreted ("vertical interpretation" for Figure 4a and "horizontal interpretation" for Figure 4b). This is carried out in the description of Figure 4:

> *Figure 4a shows the effect of hydrogen exports on domestic electricity cost. Therefore, the effect of hydrogen exports is isolated by normalizing the costs to 1 TWh/a hydrogen export in each column. This approach allows for a vertical interpretation in Figure 4a only, displaying the effects of hydrogen exports (vertical) at a certain domestic CO_2 mitigation level. Figure 4b shows the effect of domestic CO_2 mitigation on hydrogen export cost. Therefore, the effect of domestic CO_2 mitigation is isolated by normalizing the costs to 0% CO_2 mitigation in each row. This approach allows for a horizontal interpretation in Figure 4b only, displaying the effects of domestic CO_2 mitigation (horizontal) at a certain hydrogen export quantity.*

Third, we have added and integrated both Figures 4a and 4b in a non-normalized, absolute manner. We refer to it in the description of Figure 4:

> *See Figure S8 for absolute cost for domestic electricity consumers and hydrogen exporters.*

Forth, we have removed the pathway analysis in the contour plots of Figure 4a and 4b as well it's description. We fully recognize, that the conducted pathway analysis is not evident from the normalized data representation and might contribute to reader confusion.

To sum up, we fully recognize the reviewers criticism and have undertaken efforts to reduce possibility of reader confusion. While we feel that the normalization approach is indeed unusual but highlights the effects on hydrogen exporters and domestic consumers, we are open to use the non-normalized figure, breaking these out into individual columns/rows, or normalize to a single point instead, if the reviewer is in favour of those options.

RC: Line 443-499 in the discussion need to be revised with the updated results from the pathway analysis discussed above (Line 288-313). This is also true for parts of the conclusion and the abstract.

AR: We agree, that the discussion needs to be revised. As discussed above, we have removed the pathway analysis from Figure 4 (since it was not evident from the normalization approach) but instead introduced the pathway analysis in Figure 7, where we have presented three scenario groups/pathways and the impact of temporal hydrogen regulation on them. We have fully revised the discussion (previously line 288-313) in line 252:

> *The transition towards climate neutrality of Morocco's energy system and the ramping up of exports up to 120 TWh/a are a matter of decade(s) and undergo a certain pathway. However, the broad scenario design of this study allows conclusions and recommendations beyond the specific Morocco case. Various potential hydrogen exporting countries with different prerequisites can be found in the integrated analysis of benefits and burdens for domestic electricity consumers and hydrogen exporters among the dimensions of domestic CO_2 mitigation, hydrogen export and hydrogen regulation. Based on the main findings of the impact of temporal regulation on hydrogen exporters and their domestic CO_2 mitigation identified in Results, we present three possible pathways (aligned with the categorization in Figure 7) of reaching the aims of hydrogen exports and domestic CO_2 mitigation complemented with tailored temporal hydrogen regulation recommendations:*
>
> *First, at quick exports and slow CO_2 mitigation: Reaching high export levels before adequately mitigating domestic emissions leads to higher total emissions, if no strict temporal hydrogen regulation is in place. Instead, hourly matching reduces domestic electricity cost effectively and redistributes welfare from exporters to domestic consumers. This regulation plays a decisive role in this pathway, even annual or monthly matching triggers the price spillover and energy spillover effects.*
>
> *Second, at a Balanced exports and CO_2 mitigation: A balanced transition of both exports and mitigation offers benefits for both domestic electricity consumers and hydrogen exporters. At a medium (40–60%) CO_2 mitigation, domestic electricity consumers profit from progressing hydrogen exports. Vice versa, hydrogen exporters benefit from domestic CO_2 mitigation, if the hydrogen export volume is at 50–100 TWh and the domestic CO_2 mitigation advances from 0% to a medium range of (40–60%) CO_2 mitigation. A strict temporal hydrogen regulation enables these co-benefits in this pathway. Third, at Slow exports and quick CO_2 mitigation: If the transition speed of domestic CO_2 mitigation is quicker than the scale-up of hydrogen exports, there is no effect of annual or monthly temporal hydrogen regulation on costs for hydrogen exporters or domestic electricity consumers (s. Fig. 7). The temporal hydrogen regulation plays only a minor role in this pathway, since the electricity system is already highly or fully renewable electricity based and neither price spillover nor energy spillover are effective.*
>
> *The three illustrative pathways offer certain room for manoeuvre in policy-making for various countries and their export ambitions, speed of domestic CO_2 mitigation and temporal hydrogen regulation. However, these pathways do not reflect additional constraints as limited renewable energy ramp-up rates, the availability of workforce, additional prerequisites such as an effective carbon pricing system for carbon-based Power-to-X products and bureaucratic hurdles among others. A holistic approach and balanced policies are essential to guide the energy transition and support export ambitions effectively.*

In the conclusion, the statement on co-benefits in lines 594-598 requires a clarification, avoiding the oversimplification that both hydrogen exporters and domestic consumers profit in common export-mitigation scenarios. Instead, we have differentiated the statement as it is evident from the results (Figure 4) in line 348:

> *At a medium (40–60%) CO_2 mitigation, domestic electricity consumers profit from progressing hydrogen exports. Vice versa, hydrogen exporters benefit from domestic CO_2 mitigation, if the hydrogen export volume is at 50–100 TWh and the domestic CO_2 mitigation advances from 0% to a medium range of (40–60%) CO_2 mitigation.*

In the abstract, we have identified the following statement that relates to the pathway discussion and was not evident from the data presented so far: "[...] increasing hydrogen exports quickly in a system that is still dominated by fossil fuels can substantially raise domestic electricity prices, if green hydrogen production is not regulated." Previously we have not included the case of "no regulation"of Figure 4a, therefore the statement ("if [...] not regulated") was missing the counterpart. To overcome this, we have added the no-regulation sensitivity of Figure 4, where we see that the domestic electricity cost increase without regulation. This sensitivity is displayed in Figure S12 and referenced in the description of Figure 4:

> *[...] see [...] Figure S12 for the interplay of hydrogen exports and domestic CO_2 mitigation if no hydrogen regulation is in place.*

In the figure description of Figure S12, we outline that:

> *[...] increasing hydrogen exports quickly in a system that is still dominated by fossil fuels substantially raises domestic electricity prices, if green hydrogen production is not regulated.*

While any diagonal interpretation of Figure 4a is not valid as outlined by the previous comments, a vertical interpretation in Figure 4a indeed is. In our understanding, the statement is valid in terms of the interpretation, because the domestic CO_2 mitigation is fixed ("still dominated by fossil fuels") and we increase the (vertical) parameter of hydrogen exports ("increasing hydrogen exports quickly").

By integrating the no-regulation case of Figure 4, we have backed the statement with additional data. However, if the reviewer still sees additional issues with the abstract, conclusion or discussion regarding the pathway analysis, we are open to revise those as well.

RC: Line 1-21: there are no sources for your statements, please add

AR: We have added nine additional sources for the statements in the introduction, mostly from high-impact journals highlighting the importance of green hydrogen in low-carbon energy systems and the role of various countries exploring hydrogen production and use in line 2 onwards:

> *The global energy environment is experiencing fundamental upheaval, driven by the need to reduce greenhouse gas emissions and transition to a low-carbon future [1,2]. In this context, hydrogen has emerged as a viable clean energy carrier capable of addressing the issues of decarbonizing numerous sectors such as industry, transport, heating and power generation [3-7]. A growing number of countries are investigating green hydrogen production and use as a crucial component of their strategy to cut greenhouse gas emissions and meet ambitious climate targets [3,8]. Simultaneously, several countries are positioning themselves as potential exporters of hydrogen and Power-to-X products, discovering an opportunity to leverage their renewable energy resources and technological advances [9].These countries are expected to play a substantial role in the global energy market by supplying clean hydrogen and therefore contributing to global decarbonization efforts [9]. However, pursuing both (on-grid) hydrogen exports and national energy transition raises questions on welfare redistribution, prices and co-benefits that require in-depth analysis, additionally shining a light on the role of temporal hydrogen regulation.*

RC: Line 34: : https://doi.org/10.1016/j.ijhydene.2023.05.012 is another interesting source in this regard, as this paper present an approach to design local infrastructure for hydrogen export

AR: We agree, as this paper is motivated by comparable conditions as the other cited sources, deals with the African continent and is added in line 30 as additional source number 24:

> *These conditions are the motivation of several studies [16-24] examining the potential of hydrogen in Africa and synergies with European demand*

RC: Line 49: I found one really new studies in this gap: https://doi.org/10.1016/j.ijhydene.2024.04.153. Please also add this to the literature and re-elaborate the research gap (No worries, I still see a relevant research gap you close, but please update it with the newer source if applicable)

AR: We are very thankful for this valuable contribution to the literature research. Indeed, this study is of high relevance, since it combines both energy transition pathways and green hydrogen exports. Prior to this, there was no study known to us which has made this link. We have integrated the study and adjusted the literature review accordingly. While this new study is a great contribution to the research gap, it only applies low temporal and spatial resolution models, has a limited set of scenarios, does not identify synergies and conflicts for hydrogen exporters and domestic electricity consumers nor does it differentiate between those interest groups. Lastly, it does not consider the impact of temporal hydrogen regulation on hydrogen exports and energy transition pathways. However, their study design successfully combines the dimension of domestic energy transition and green hydrogen exports, and we are very thankful to this valuable contribution. We have outlined those points in line 38:

> *A recent study by Müller et al. [30] contributes to this research gap by analysing energy transition pathways and green hydrogen exports in Algeria. They identify impacts on total system cost along four scenarios by applying an energy model with limited spatial (one node) and temporal (288 time slices per year) resolution. While this approach is a valuable contribution to this research gap, the study does not specifically outline synergies and conflicts for hydrogen exporters and domestic electricity consumers and differentiate between those interest groups. Due to their limited spatial and temporal resolution, this approach is unsuitable to identify transmission bottlenecks, infrastructure expansion requirements, and detailed system dynamics. However, their study design successfully combines the dimension of domestic energy transition and green hydrogen exports.*

In addition to analysing this study in depth, we have adjusted our summary of studies where we have previously stated that "studies focus exclusively on either hydrogen exports, domestic climate mitigation or temporal hydrogen regulation, without discussing the complex relationship between these three dimensions". Since this new study does not exclusively focus on hydrogen exports or domestic CO_2 mitigation but indeed combines those two, we modified line 59:

> *While most of the studies presented above focus exclusively on either hydrogen exports, domestic climate mitigation, or temporal hydrogen regulation, only one study [30] does combine two of these aspects. However, none of them address the complex relationship between all three dimensions.*

RC: Line 45: The paper https://doi.org/10.48550/arXiv.2407.07573 could be interesting to mention in this regard, as this paper presents a methodology for evaluating the impact of hydrogen export on social factors and local jobs

AR: This line of research blends in with the listed studies and is added in an additional line 35:

> *Ishmam et al. [29] assesses the impact of hydrogen exports on social factors and domestic jobs, going beyond a simplified energy potential analysis.*

RC: Line 57: “point out benefits of … ” on what? hydrogen cost, emissions, electricity costs..?

AR: We agree and clarify in line 49 onwards that the effect is on project design, economics, and power sector emissions:

> *[…], whereas Ruhnau et al. [32] points out the effect of relaxing simultaneity requirements of RE supply and hydrogen generation on project design, economics, and power sector emissions.*

RC: Line 59 ff: If the source is worth mentioning, please also state their relevant outcome: ”The effect of regulatory options on social welfare and carbon emissions is assessed in Brauer et al.”, with the outcome of …

AR: We agree that the relevant outcomes are worth mentioning and add them in line 50:

> *The effect of regulatory options on social welfare and carbon emissions is assessed in Brauer et al. [33]. They recommend that the regulation on the spatial dimension may not be too stringent, however they outline that the benefits of strict temporal matching (e.g. a decline in emissions) exceed the moderate economic disadvantages. In addition, the need for such regulation diminishes in highly renewable electricity systems.*

RC: Line 70: “Numerous studies focus on” but no citations. What does this exactly refer to? The stated studies above? Please clarify

AR: Indeed, this refers to the stated studies above. The sentence is clarified to avoid confusions in line 59:

> *While most of the studies presented above focus exclusively on either hydrogen exports, [. . .]*

RC: Line 79: “sector coupled” please specify which sector are coupled. Probably, electricity and hydrogen are meant, but in theory various sectors could be coupled.

AR: The sector-coupled model presented in this study spans across various sectors. While multiple energy carriers are included (electricity, hydrogen, natural gas, biomass, oil, and heat, as well as carbon dioxide in the form of emissions and feed-stock for the synthesis of other carriers), we distinguish various demand sectors: Residential, Services, Transport (road, rail, aviation and navigation), Industry, Agriculture. This is explained in detail in the Methods Section in line 394:

> *The resulting temporal and spatial energy demands of the sectors (i) residential, (ii) services, (iii) industry, (iv) transport (road, rail, aviation and navigation) and (v) agriculture are integrated in the sector coupled energy model, including the energy carriers electricity, hydrogen, natural gas, biomass, oil, and heat, as well as carbon dioxide in the form of emissions and feed-stock for the synthesis of other carriers (s. Fig. S19).*

However, we appreciate the remark to include a specification earlier, as suggested by the reviewer in line 64:

> *This study introduces a sector-coupled energy model (encompassing the residential, services, industry, transport and agriculture demand sectors) for the target region, [. . .]*

RC: Line 85: This overview is nice and helpful to understand the scope of analysis. Maybe this is a good point to explain the difference between “no regulation” and “annual” matching as raised in my comment for line 325

AR: We highly appreciate the suggestion to explain the difference between "no regulation" and "annual" matching in this overview as outlined in the comment for line 325. The clarification is added in the overview, line 71:

> *The temporal matching regulation is implemented via a mimimum constraint, stating that the renewable electricity generation is required to be equal or higher than the electricity demand for electrolytic hydrogen within every year (annual matching), month (monthly matching) or hour (hourly matching). In the no regulation case, no such minimum constraint is applied.*

RC: Line 85: "Domestic climate change mitigation %" or "Domestic CO2 mitigation %"? That's a difference. Generally, you seem to use climate change mitigation and CO2 mitigation as a synonym, which they are not. Please explain or adapt.

AR: Indeed, we have used both the terms *domestic climate change mitigation* and *domestic* CO_2 *mitigation*, without differentiating precisely. We have removed the term *climate change mitigation* throughout the whole manuscript, and now only refer to *domestic* CO_2 *mitigation* only. The "CO_2 "-abbreviation is first introduced in line 46:

> *Apart from hydrogen exports and domestic carbon dioxide* (CO_2) *mitigation, [...]*

RC: Line 111: Probably the data source for global administrative areas (GADM?) is missing

AR: Indeed, the data source was added in line 84:

> *Since the 14 nodes align with the Global Administrative Areas level 1 regions [37], [. . .]*

RC: Line 160: if the citation is meant for the whole paragraph, it should be behind the point

AR: Indeed, the citation of the Moroccan Hydrogen Strategy is meant for the whole paragraph. While adding the citation for the whole paragraph behind the point of the last sentence of the paragraph is a valid solution for most disciplines and journals, we have decided to integrate the citation wherever necessary within the paragraph to prevent possible confusions. The revised paragraph starts in line 13:

> *Figure 1 shows the hydrogen strategy of Morocco [9], including national and export demands. By 2030, the total hydrogen demand adds up to 13.9 TWh/a and ramps up to 67.9 TWh/a in 2040 and 153.9 TWh/a in 2050, clearly listing higher demands for export than for domestic use. The hydrogen generation is backed by 5.2 GW of RE in 2030, 23 GW in 2040, and 57.4 GW in 2050 in the export sector [9]. To cover the national demand of hydrogen, a Renewable Energy deployment of 1.6 GW in 2030, 7.0 GW in 2040 and 10.3 GW in 2050 is planned in Morocco's Hydrogen Strategy [9].*

RC: Line 167: abbreviation LULUCF without explanation (Land Use, Land Use-Change and Forestry?)

AR: We agree with the reviewer, LULUCF was used as an abbreviation for Land Use, Land Use-Change and Forestry but it was not explained. Following the guidelines of the journal which requests to keep the number of abbreviation to a minimum, we decided to write out the abbreviation in line 19:

> *Morocco aims to limit historically rising greenhouse gas emissions to 75 MtCO_2e (conditional) respectively 115 MtCO_2e (unconditional) excluding emissions of Land Use, Land Use-Change and Forestry by 2030 [10].*

RC: Figure 4: Please explain the definition of relative costs in the figure as well. The x label could be more specific like "Domestic CO_2 mitigation %"

AR: We have revised the definition of relative costs in the figure description of Figure 4:

> *Figure 4a shows the effect of hydrogen exports on domestic electricity cost. Therefore, the effect of hydrogen exports is isolated by normalizing the costs to 1 TWh/a hydrogen export in each column. This approach allows for a vertical interpretation in Figure 4a only, displaying the effects of hydrogen exports (vertical) at a certain domestic CO_2 mitigation level. Figure 4b shows the effect of domestic CO_2 mitigation on hydrogen export cost. Therefore, the effect of domestic CO_2 mitigation is isolated by normalizing the costs to 0% CO_2 mitigation in each row. This approach allows for a horizontal interpretation in Figure 4b only, displaying the effects of domestic CO_2 mitigation (horizontal) at a certain hydrogen export quantity. [. . .]*

We are thankful for the suggestion, and have changed the x-label of Figure 4 (and all other contour plots in the main text and supplementary material too) to:

> *Domestic CO_2 mitigation in %*

RC: Line 248: "electricity system profits from excess green electricity for H2 export". Why so? Is the curtailment decreased at higher export rates?

AR: We are thankful for the comment. The term "electricity system" was not selected carefully, since we want to express the benefits for domestic electricity consumers and not the system itself. We have adjusted in line 142:

> *In these ranges, the domestic electricity consumers profit from excess green electricity originating from additional renewable energy capacities required for hydrogen export.*

RC: Lien 263: "decreases the hydrogen export prices up to -9%." Its actual costs not prices as named in the figure 4.

AR: We agree, the terminology *costs* is correct in this context. We have adjusted in line 150:

> *At hydrogen export volumes of 25–75 TWh, an increase of domestic CO_2 mitigation up to 40–60% decreases the cost for hydrogen exporters up to -9%.*

Furthermore, to avoid reader confusion, we have clarified the terminology on *costs* and *prices* throughout the whole manuscript and briefly described in line 97:

> *We use costs referring to model inputs and system costs, while prices denote endogenous shadow prices (representing idealized market prices) derived from the model. Payments made by consumers (calculated as energy price times energy volume) are described as (consumer) costs or, alternatively, (consumer) payments to highlight their financial impact.*

RC: Line 263: "decreases the hydrogen export prices up to -9%." Why so? I think this point and the point from Line 248 are relevant for your conclusion in the following way: If the reason for both results is a universal property of exporting hydrogen energy systems under CO2 mitigation, then your conclusion in line 490 ("The three illustrative pathways offer certain room for manoeuvre in policy-making for various countries") is valid. If the reason for decreasing hydrogen cost just that this specific energy system situation comes from e.g. specifically cheap potentials in this area for Morocco, then the conclusion is only valid for Morocco. I think a short evaluation helps supporting your conclusions drawn.

AR: Thank you for this insightful comment, we support the suggestion to add an evaluation to clarify why hydrogen export prices decrease at moderate export volumes under increased domestic CO_2 mitigation. This effect results from synergies in shared infrastructure, which reduce costs within this hydrogen export range. At higher export levels, the effect diminishes as the hydrogen exports surpass the domestic electricity system, and at even higher CO_2 mitigation levels, less optimal renewable generation sites drive costs up. Those effects (benefits of common infrastructure, hydrogen exports surpassing the domestic electricity system) as well as the remark on line 248 (outlined above) are universal across various energy systems, supporting the broader applicability of our conclusions. We have added the evaluation in line 150:

> *At hydrogen export volumes of 25–75 TWh, an increase of domestic CO_2 mitigation up to 40–60% decreases the hydrogen export prices up to -9%. Increasing the domestic CO_2 mitigation requires additional system flexibility through BEV charging, energy storage and transmission capacities (s. Fig. 3a), which benefits hydrogen exporters as well and effectively reduces hydrogen export costs. However, at even higher levels of domestic CO_2 mitigation (70–100%), further renewable energy expansion is required, tapping into sites with less favorable weather conditions in Morocco, which ultimately raises electricity costs (s. Fig. S13d).This effect diminishes at higher export volumes, where the hydrogen export system becomes significantly larger than the domestic electricity system, reducing the relative impact of shared infrastructure as shown in Figure 4b (see also the sensitivity on higher exports in Figure S17b).*

RC: Line 271: failed reference probably to figure S10b)

AR: The reference failed, indeed the reference is meant to point at Figure 10b. In addition, the full stop at the end of the sentence was missing and is now added in line 160 along with the figure reference:

> *[. . .], where we observe high electrolysis capacity factors of above 80% (s. Fig. S13b).*

RC: Line 325: "hourly basis": the model is at a resolution of 3 hours (line 97); how can the time matching be at an hourly basis?

AR: We agree with the reviewer, that the statement "hourly basis" seems to contradict the applied 3-hourly temporal model resolution.

Fist of all, we would like to emphasize why we have chosen a three-hourly resolution in the first place: Given the broad set of scenarios (three dimensions: temporal matching, CO_2 mitigation and hydrogen export) and the high spatial resolution, we have chosen a three-hourly temporal resolution (instead of e.g. hourly) in our study design to stay within computationally feasible limits and still capture energy system dynamics (e.g. RE generation profiles, energy storage operation) with its diurnal and seasonal variations in energy supply and demand. We elaborate this decision in detail in line 78 in detail:

> *A 3-hourly resolution is chosen to capture energy system dynamics (e.g. renewable energy (RE) generation profiles, energy storage operation) with its diurnal and seasonal variations in energy supply and demand. Similar studies [35,36] find a minor underestimation of short-term battery storage and onshore wind and a minor overestimation of solar photovoltaics (PV) and hydrogen storage compared to an hourly resolution, overall justifying the reduction of the model size.*

However, we fully agree with the reviewer, that the statements "three-hourly model" and "hourly basis" seem logically contrary. Nonetheless, the effects of hourly temporal matching can indeed be investigated with a three-hourly model. Assuming an hourly model resolution, the hourly matching constraints the electricity demand for hydrogen electrolysis *at all timesteps, 8760 steps per year.* The same mechanism applies for a three-hourly model, since the "hourly matching" constraints the electricity demand for hydrogen electrolysis *at all timesteps, 8760/3=2920 steps per year* as well.

This is well complemented by the mechanism of the other temporal regulation regimes (monthly, annual, no regulation) we have investigated in our study. E.g. applying monthly matching, the electricity demand for electrolysis may indeed be supplied by non-renewable electricity at certain hours, as long as the monthly balance equal. In this case (monthly or annual matching), the description *at all timesteps* does not hold, unlike at the hourly matching regardless whether it is modelled with hourly or three-hourly resolution.

To conclude, the technically correct term of hourly matching which holds for hourly as well as three-hourly model resolution would be *"matching at all times"*, *full matching*, or similar. However, the underlying mechanism of *"matching at all times"* and *hourly basis* are the same. We understand, that the term *hourly basis* may confuse the reader in this context and adjust in line 176:

> *As the temporal matching becomes stricter, additional renewable capacities and complementing hydrogen storage are required to meet the electricity demand of electrolysis on a time base defined by the temporal matching constraint, as shown in Figure S11.*

In addition, we are also aware that the term *hourly matching* might confuse readers. Given it's policy relevance and comparability to other studies[1, 2], we would prefer keeping *hourly matching* over *"matching at all times"*, *full matching*, or similar. However, if the reviewer prefers those statement, we are open to consider using them instead of *hourly matching* throughout the whole study. Either way, to avoid that confusion, we specify in line 73 where the term *hourly matching* is first introduced:

> *We use the term hourly matching despite our three-hourly model because the principle of matching electricity demand for electrolysis and renewable electricity supply at each timestep is the same. This term effectively captures the mechanism, regardless of the model's resolution.*

RC: Line 325: Figure S16 implies that you optimized the operation of 1 year. If so, what is the difference between "no policy" and "annual"? for annual, can only green hydrogen be exported and for no policy, also hydrogen from brown coal can be exported? Please clarify

AR: Indeed, the annual temporal matching policy describes that the renewable electricity generation is required to be equal or higher than the electricity demand for electrolytic hydrogen within one year. Analogue to this, the electricity generation within every single month (for monthly matching) or hour (for hourly matching) is required to be equal or higher than the electricity demand for electrolytic hydrogen. In the no policy/rule case, no such minimum constraint is applied, hence the electricity demand for hydrogen electrolysis could be partly met by brown coal or other energy carriers. This can be observed in Fig. 6a, where in the annual case the electricity demand for hydrogen electrolysis is met, whereas in the no policy/rule case the electricity generation from renewables is lower than the demand for hydrogen electrolysis. As suggested in a previous comment, this clarification is added in line 71:

> *The temporal matching regulation is implemented via a minimum constraint, stating that the renewable electricity generation is required to be equal or higher than the electricity demand for electrolytic hydrogen within every year (annual matching), month (monthly matching) or hour (hourly matching). In the no regulation case, no such minimum constraint is applied.*

RC: Line 326: typo: "marginal cost OF fossil [...]"

AR: We appreciate the careful review. The phrase "high marginal cost fossil generation" is grammatically correct, with "high marginal cost" serving as adjectives describing the higher marginal cost of fossil generation in comparison to the lower marginal cost of renewable generation. The structure mirrors common usage, where adjectives modify a noun directly (e.g., "low carbon emissions"). However, we recognize the lack of readability and possible confusion, therefore we propose to rephrase it as "fossil generation with high marginal costs" as edited in line 178:

> *These renewable capacities i.) push out fossil generation with high marginal costs (price spillover) and ii.) provide excess electricity to the domestic electricity system (energy spillover).*

RC: Line 343: missing reference

AR: Thank you for the careful review, the figure reference was missing. Instead of simply adding the reference, we have decided to rephrase the sentence to improve the clarity, avoid unnecessary references to figures in the supplementary information, and to better link the explanation to the main Figure 6a. The sentence is rephrased in line 190:

> *[…], the electricity supply of coal and OCGT is substantially higher than in the annual, monthly, or hourly case.*

RC: Figure 6: What is exactly the difference to Figure S9? S9 shows the total annual cost which are strictly increasing as the feasible space is further confined by temporal limitations. Until this point, I can follow your results. F6b) has decreasing "cost for consumer" with them being only part of the total annual costs. What are those cost exactly and why do they decrease? Do the consumers not have to pay the full total annual cost? Please clarify the difference.

AR: Thank you for this observation. We fully agree, the total annual cost are strictly increasing as the feasible space is further confined by temporal limitations. The differences between total system costs and consumer costs result from contributions to existing brownfield coal power assets, which are excluded from the optimization objective function but factored into consumer costs. Stricter temporal hydrogen regulations lead to reduced coal utilization, decreasing costs for domestic electricity consumers, while hydrogen exporters see a slight increase due to additional renewable electricity installation costs. This distinction, along with the reasons for the decreasing discrepancy between system costs and consumer costs under stricter regulations, is clarified in the revised manuscript in line 206:

> *The combined cost borne by domestic electricity consumers and hydrogen exporters decreases with stricter temporal hydrogen regulation, while the overall system cost increases, as shown in Figure S11. This difference arises because electricity consumers (both domestic and hydrogen electrolysis) pay a contribution margin to the capital costs of existing coal power plants, which are pre-existing brownfield assets. Since these brownfield capacities are not part of the objective function, their capital costs are excluded from the total system cost (s. Fig. S11) but are included in consumer costs (s. Fig. 6b). Under stricter temporal hydrogen regulation, coal power phases out, reducing the capital contributions required from consumers for these coal assets. For domestic electricity consumers, this leads to a direct decrease in their cost. However, for hydrogen exporters, the cost slightly increases, as they bear the cost for the additional renewable capacities (s. Fig. S11) required to meet temporal hydrogen regulation. As coal capacity declines with stricter hydrogen regulation, the discrepancy between consumer costs and the total system cost decreases. This observation also holds if the coal capacity is decreased under high decarbonization scenarios where fossil fuel dispatch is minimized, leading the system closer to an equilibrium where marginal prices match the optimization's objective function.*

If the reviewer suggests adding a graphical representation (e.g. in the supplementary material, along with Figure S11) of these mechanisms, we would welcome an opportunity to do so.

RC: Line 388: something is missing. maybe "steers the redistribution OF CONSUMER COSTS between ..."

AR: We appreciate the proposal and agree, that not the redistribution of *exports or consumers* is steered by the temporal hydrogen regulation, but in instead the *costs* are. We accept the proposal to include the "of costs" and dismiss the "consumer" in this context, since the costs refer to not only the (domestic) consumer cost but also to the hydrogen export cost (which are no consumers). However, if the proposed term "of consumer costs" provides more clarity, we are open to accept the proposal as is. The modification is displayed in line 217:

> *It steers the redistribution of costs between hydrogen exports and domestic electricity consumers [...]*

RC: Line 392: "The energy spillover effect not only decarbonizes hydrogen exports...". I don't see how the "energy spillover" decarbonizes the hydrogen export. You defined the energy spillover as "provide excess electricity to the domestic electricity system" (line 327). So, when the green hydrogen production is limited by the temporal matching, excess green electricity is dispatched to the domestic grid reducing the dispatch of coal and therefore grid and total emissions. But by that definition energy spillover cannot impact the hydrogen emissions, as it only takes leftover energy to decarbonize local emissions, right? Also, with temporal matching of renewables to electrolysis, the exported hydrogen should not have any emissions at all. The introduction of temporal matching leads to a reduction of export hydrogen emissions as it introduces a policy for green hydrogen, but the spillover does not.

We are thankful for the thoughtful comment on the mechanism on the energy spillover. We fully agree with the reviewer, the mechanism of energy spillover originates from temporal matching of hydrogen exports and effects the domestic grid (emissions), and not the other way round as implied by our statement *"The energy spillover effect not only decarbonizes hydrogen exports . . . "*. We correct the logic of the sentence and state the cause (temporal matching) and the effect (decarbonizes hydrogen exports and grid emissions) as originally intended (but previously expressed wrongly) in line 219:

> *Second, the temporal hydrogen regulation not only decarbonizes hydrogen exports, but additionally decreases grid emissions of the domestic electricity demand via the energy spillover effect.*

Second, we would like to clarify the reviewer comment *"with temporal matching of renewables to electrolysis, the exported hydrogen should not have any emissions at all"*. In principal, we agree with this assessment. Especially under the hourly temporal matching, the electricity demand for hydrogen electrolysis is covered by renewable electricity at every single hour of the year, hence the exported hydrogen does not have any emission at all (if life cycle emissions of renewables/-electrolyzers etc. are not considered). In the case of annual matching however, the electricity demand for hydrogen electrolysis needs to be covered by renewable electricity on an annual basis only. In this case, there may be single hours within a certain year, in which the demand is (partly) covered by fossil dispatch. In return, renewable electricity is dispatched to the grid to fulfill the annual matching balance. Depending on where those emissions are accounted to (grid or hydrogen electrolysis), hydrogen exports are only fully, at all times, zero-emission if hourly matching is applied. This finding is supported by recent studies, e.g. Zeyen et al. [1].

To sum up, we are hope that this differentiation contributes to the understanding of this reviewer comment and our reply. If the reviewer would like to see this detailed differentiation in the manuscript as well, we would welcome the opportunity to do so. However, we think the main mechanisms are outlined sufficiently in the manuscript already, especially from line 339 onwards of the original draft.

RC: Figure 7: I like that you chose a scale starting from 0 to show the actual deviation and therefore being unbiased.

AR: Thank you for your positive feedback on Figure 7. We are glad the choice of scale effectively conveys the main message in an unbiased manner.

RC: Line 401: “across the pathway”: as far as I understood the model right, there is no pathway modelled. Do you mean the “annual optimized 2030 energy system designs across the pathway”? Because this somehow implies that you calculated a coherent pathway, which I think you did not.

AR: Indeed, the model does not consider coherent pathways but single, annual energy system designs are modelled. In line 401, we have completely revised the discussion on pathways and therefore removed the statement "across the pathway" in line 401 of the original manuscript. In addition, we now introduce the concept of pathways earlier, and define it more precisely line 225:

> *We have grouped these scenarios into three illustrative mitigation-export pathways presenting various speeds of transformation, [...] further elaborated in Figure S15a:*

RC: Line 521: On the other side exporting hydrogen is a chance to boost local economies as shown in https://doi.org/10.48550/arXiv.2407.07573

AR: Thank you for the reference suggestion. We agree with the importance of highlighting the role of hydrogen exports in boosting local economies. We decided to incorporate the citation one paragraph earlier, as it aligns well with the discussion on opportunities for domestic businesses and households. However, if the reviewer prefers, we can also include it in the location initially suggested. The reference is added in line 282:

> *The export of hydrogen in combination with strict temporal hydrogen regulation provides the opportunity of decreasing domestic electricity prices, providing opportunities for domestic businesses and households. The chance to boost local economies by exporting hydrogen is also highlighted in Ishmam et al. [20].*

RC: Line 540: For the limitation, you should mention that you did not calculate transition pathways but instead approximated them by using myopic energy systems for 2030 and if there are limitations arising therefrom. Maybe a short comparison to https://doi.org/10.1016/j.ijhydene.2024.04.153 helps spotting methodology differences in myopic energy systems on a transition path vs. actual transition paths.

AR: Thank you for your valuable feedback. We appreciate your suggestion to clarify our approach regarding the use of a fixed, single year in comparison to actual transition paths. Since we have added a detailed elaboration on the fixed-year approach on request of other reviewers in the methodology section, we have decided to subsequently discuss the limitations of our approach highlighting the methodological differences in line 373:

> *The model is based on the economic year of 2030, optimizing the time span of a full year. We have chosen the year 2030 to account for the relevance of hydrogen exports and domestic CO_2 mitigation in the near-term. However, we do not expect or imply that both substantial hydrogen export volumes and the domestic CO_2 mitigation fully materialize by the year 2030 given the time span required for the ramp up of the required technologies (Renewable electricity, hydrogen electrolyis, etc.). By fixing the modelling year to 2030 instead of modelling transformation pathways, we can isolate the effects of 0-100% mitigation (to today) and 0-120 TWh hydrogen export and contextualize our results in a comparable setting. However, this fixed-year approach has limitations. Modeling actual transition pathways (instead of a fixed year) accounts for evolving factors over time, such as technology costs and regulatory shifts. In comparison, Müller et al. [30] employs such an approach by exogenously defining the final energy demand, hydrogen exports and emission constraint across a transition path up to 2050. Based on these input parameters, the cost-optimal power sector is modelled endogenously. This allows for a more detailed exploration of transition pathways, and enables insights of reaching (annual) climate goals under a (cumulative) CO_2 budget.*

However, if the reviewer suggests moving the discussion of our approach and methodological differences to Müller et al. [3] to the limitations sections, we would welcome an opportunity to do so.

RC: Line 565: There is a publication which exactly quantifies the effect of storage sizes for LH2 export: https://doi.org/10.1016/j.ijhydene.2023.05.012 Figure 6 evaluates the cost share of hydrogen storages for export being below 5% cost for solar regions. Just as a further reference.

AR: Approved. We are thankful for the additional source, it quantifies our assumption and is therefore added in line 311:

> *As findings of Franzmann et al. [24] point out, the cost share of hydrogen storage for export is below 5% of the final product costs in solar regions, while slightly higher for wind-dominated regions.*

RC: Line 633: The setting of the model seems to unclear. Based on the methodology a transformation pathway is optimized? Is only on year optimized with the assumed CO2 reduction of 0-100% to today? If yes, which economic year and why? Is it 2030 because of line 715? What time span was optimized? As Figure S16 implies one year? Please define the general setting of the model (in methodology or the model section in the introduction). Also, a system overview (e.g. in the supplementary data) would be helpful, as you also described the impact of hydrogen demand on P2X for combustion engines demand. From the description within the manuscript, I would have never initially guessed, that this is modelled as well.

AR: Thank you for your comment. We agree, that a clear definition of the model setting is beneficial for the understanding of the study. We have added a detailed description in the Method section in line 373:

> *The model is based on the economic year of 2030, optimizing the time span of a full year. We have chosen the year 2030 to account for the relevance of hydrogen exports and domestic climate change in the near-term. However, we do not expect or imply that both substantial hydrogen export volumes and the domestic CO_2 mitigation fully materialize by the year 2030 given the time span required for the ramp up of the required technologies (Renewable electricity, hydrogen electrolyis, etc.). By fixing the modelling year to 2030 instead of modelling transformation pathways, we can isolate the effects of 0-100% mitigation (to today) and 0-120 TWh hydrogen export and contextualize our results in a comparable setting.*

We also agree on the benefit of adding a system overview. We have added it to the supplementary material and refer to it in the main text in line 372:

> *A more detailed mathematical problem formulation is provided in Section Mathematical Model Formulation, and a system overview is provided in Figure S1.*

RC: Line 641: Maybe you can add a sentence about the demand of fuels, as this would explain the following sentence below Figure S9: "The large share of oil OPEX is independent of temporal hydrogen regulation, since these costs are mainly linked to combustion engine cars with demands independent of temporal hydrogen regulation."

AR: We agree, that an additional sentence about the demand of fuels supports the understanding of the study in general and Figure S9 in particular. Following other reviewer comments, the section on energy demand is extensively revised and better outlines the relationship between energy services, final energy and primary energy. In addition, and in specific reply to this comment, we add in line 402:

> *In the case of low domestic CO_2 mitigation, a low BEV share results in a high fuel demand for e.g. Internal Combustion Engine Cars, as observable in Figure S11 [author's note: previsouly Figure S9]. This reflects a constant energy service demand (transportation) while the primary energy is subject to CO_2 mitigation.*

RC: Line 677: Which weather year was used for the model?

AR: We appreciate the remark to specify the weather year, the year 2013 was used for the study. In addition, it is clarified that the ERA5 reanalysis dataset is the only weather data source which is used, whereas the SARAH2 dataset was not applied unlike previously implied. These modifications are added in line 407:

> *Atlite obtains technology-specific time series of the weather year 2013 based on the ERA5 reanalysis data [70], [...]*

RC: Line 678: "[...] of 36". I know the rule to not cite numbers directly but always add the name in front.

AR: We agree, the name is added in front of the citation in line 406:

> *The sector-coupled energy model follows the data pipeline of Parzen et al. [58] by incorporating solar PV, onshore wind and hydro power using the open source package Atlite [69].*

RC: Line 707: same as for Line 678

AR: The authors agree, the name is added in front of the citation in line 423:

> *Following the workflow of Parzen et al. [58], Figure [...]*

RC: Line 715: the link is wrong; the right one is probably: https://github.com/PyPSA/technology-data. But still, there are several data set linked. Please cite the techno economic data clearly, as they are highly relevant for comparison and evaluation. Ideally, you are listing them directly in the paper

or supplementary data to avoid confusion. I can only guess that the techno-economic data are coming from https://github.com/PyPSA/technology-data/blob/master/inputs/costs_PyPSA.csv

AR: We are thankful for the thorough test of the repository link, it is indeed wrong in the line stated. We fixed the link to the (high-level, general) technology-data repository. In addition, we have added the precise version, year and dataset applied in this study as an additional link. We highly agree on the importance of listing the economic data clearly, to foster the comparability across models and studies. If the reviewer would prefer a complete list in the paper or supplementary data, we are happy to fulfill that request. The modification can be found in line 427:

> *All technology and cost assumptions are based on the year 2030, taken from the technology-data repository. This study applies version 0.4.0 of the repository, which is available at technology-data v0.4.0.*

In addition, we have specified the precise version, year and dataset in the *Data availability* section in line 476:

> *A dataset of the model results is available on Zenodo under a CC-BY-4.0 license. Technology data was taken from the technology-data repository. This study applies version 0.4.0 of the repository, which is available at technology-data v0.4.0.*

RC: Line 716: Currently, in the academic of global hydrogen the global interest rates are a highly discussed topic. Is this number "13%" just assumed or derived from a source. If second, please provide a proper citation.

AR: We agree, the interest rate is highly discussed and crucial to CAPEX-intensive technologies investigated in this study. Therefore, we have added a comprehensive explanation and sources reasoning the interest rate of 13% applied in this study in line 428:

> *Based on a comprehensive survey of industry and academia in 2024 by Fernandez et al. [78], which reports a combined Market Risk Premium and Risk-Free Rate of 12.9% for Morocco, we follow their findings and apply a discount rate of 13% in this study. While IRENA [79] reports a technology-specific interest rate of 9.1% for utility-scale PV in Morocco, we apply a more conservative approach following Fernandez et al. [78] taking into account possible higher risks of less-established technologies as water electrolysis and hydrogen infrastructure compared to utility-scale solar PV.*

RC: Technology is unclear: what kind of electrolysis are you using? PEM; SOFC, alkaline electrolysis? Please mention that

AR: We agree with the reviewer, that the type of deployed electrolysers is relevant with regards to cost of hydrogen production and system operation in general. We respond by elaborating on the electrolyser type in detail in line 471:

> *For the electrolysis, we use alkaline electrolysers due to their maturity, durability, and lower costs compared to proton exchange membrane electrolysers [45,89]. Alkaline electrolysers are well-suited for large-scale use, though they have slower start-up times and a more limited operating range than proton exchange membrane electrolysers [89,90], which, in contrast, have shorter cell lifetimes as pointed out in Staffell et al. [89].*

RC: Line 735 and 737: direct use of citation numbers in text

AR: We agree with the reviewer comment, the names are added in front of the citation in line 442:

> *[…] due to treatment chemicals and high salinity as discussed by Thomann et al. [84], Dresp et al. [85] and Tonelli et al. [86], highlighting the importance of a careful site selection of desalination plants. A minimum distance of four kilometers from marine protected areas is recommended in Thomann et al. [84].*

RC: The code is made available to a high degree. It includes a well-documented code package as well as installation guides and can therefore be reproduced. This is true for the code of the optimization model as well as the conducted result analysis. The only thing not being available are the input data as well as the result data.

AR: Thank you for reviewing the code and the documentation and the positive feedback on it's reproducibility. Apart from the source code, we have also published all result data in an open repository on Zenodo. To make users aware of it, we have now added the link to the results to the Readme of the GitHub repository of this study and explicitly mention it in line 476 in the manuscript:

> *A dataset of the model results is available on Zenodo https://doi.org/10.5281/zenodo.10951650 under a CC-BY-4.0 license.*

In addition, all techno-economic input data is collected in an open repository and linked in line 476 in the manuscript:

> *Technology data was taken from the technology-data repository https://github.com/pypsa/technology-data. This study applies version 0.4.0 of the repository, which is available at https://github.com/PyPSA/technology-data/blob/v0.4.0/outputs/costs_2030.csv.*

Additional input data (as weather data, power generation and transmission, industry data, etc.) is fetched and processed in the PyPSA-Earth-Sec model. The full data pipeline is documented on GitHub and now also linked on the Readme of the GitHub repository of this study.

Reviewer #3

RC: 1. The key issue of the methodology is that the authors essentially treat the energy economy of a country as an engineering problem. That is, they take the perspective of a central planner and calculate the lowest cost required for the generated (and transmitted) energy to meet given demands under different constraints. But economies almost never work that way. They rely on markets and market participants making their own decisions. Regulators can set market requirements and incentivize market participants to pursue certain goals (e.g., emission reductions). The problem with the approach taken by the authors is that it fundamentally limits the contribution and real-world insights of the analysis. The approach can effectively only characterize particular relationships (e.g., between the export volume of electrolytically produced hydrogen, the emissions from power generation, and the total cost of the power generation system). Quantitative estimates of specific outcomes ignore the complexity of market economies and are, therefore, prone to error.

AR: We appreciate your thoughtful feedback, particularly regarding the potential limitations of treating the energy economy as an engineering problem from the perspective of a central planner. We would like to clarify our approach and its applicability within the context of normative scenario analysis.

In normative models, it is common to explore socially optimal solutions, where the goal is to determine the most efficient way to design systems under various constraints (e.g., emissions reduction, cost minimization). This "central planner" approach allows us to evaluate idealized outcomes and serves as a benchmark for policymakers. The first fundamental theorem of welfare economics also supports the concept that, under certain conditions, market outcomes can mirror socially optimal solutions through the coordination of decentralized decision-making by endogenous prices. In our model, these endogenous prices align the decisions of decentralized actors, which supports the pathway to globally optimal outcomes. Therefore, our results can offer valuable insights by highlighting the direction in which market policies should aim. Furthermore, this approach is widely used in energy modeling studies and reports [1, 3, 4, 5, 6, 7, 8, 9, 10, 11, 12, 13, 14, 15, 16, 17]. These studies, among many others, rely on normative scenarios to assess pathways toward achieving societal goals, such as decarbonization or cost reductions. The use of these models reflects their importance in exploring optimal pathways, which can then guide the design of policies that bring real-world outcomes closer to these ideals.

We fully recognize that market economies function through decentralized decision-making, with numerous actors and unpredictable behaviors. However, models like ours provide a framework to explore key trade-offs and pathways, contributing to the broader discussion on how policy and regulatory frameworks can incentivize market actors to move toward more socially optimal outcomes. Importantly, the endogenous prices within our model helps coordinate decentralized actors' decisions, thus reflecting some market dynamics while also adhering to the principles of welfare economics.

The energy modeling community is aware of these limitations. While our model provides one perspective, it is crucial to note that policy design plays a decisive role in shaping actual market outcomes. We agree that this is only one type of model among many that aim to understand the complexities of energy economies. By framing our analysis as a tool to identify potential pathways rather than predicting exact market outcomes, we believe it retains its relevance and utility. We are thankful for the remarks on the limitations of our applied methodology (and of similar studies using normative scenarios in general) and have addressed these concerns in the revised manuscript in line 340:

> *Additionally, our model employs a normative scenario approach, which assumes that energy systems are designed to achieve socially optimal outcomes under given constraints (e.g., cost minimization, emissions reduction). While this approach is commonly used in energy system models [2,3,8,30,31,38,53-62], it does not fully capture the complexity of real-world market dynamics, where decentralized decision-making by multiple market participants plays a critical role. Nevertheless, by including endogenous prices to coordinate decentralized actors, our model provides a representation of market-based coordination. Thus, the results should be interpreted as idealised scenarios that highlight possible pathways, with actual market outcomes likely to differ due to a variety of economic and behavioral factors as outlined in Tan et al. [63].*

RC: 2. Given the approach taken, the authors should focus on characterizing relationships between the variables of interest. Instead, however, they primarily present their estimates of these variables without adequately explaining the mechanisms driving the estimates. As a result, the analysis offers limited real-world insights, restricting the implications to the specific calculations conducted by the authors.

AR: Thank you for your insightful comment. We appreciate your feedback on the analysis. In the original manuscript, we have made an effort to highlight the relationships between the variables of interest and have provided some, although not sufficient, examples of existing mechanisms driving the estimates. We fully accept the criticism and have added additional explanations characterizing the relationships between key variables in the revised manuscript.

We elaborate, how the cost-competitiveness of renewable electricity (mechanism) drives the deployment of renewable capacities (result) in line 111:

> *While mitigation targets can (in part) be met with technologies like fossil gas with CCS or Direct Air Capture, the model finds that increasing renewable capacities, particularly solar PV and wind, is the most cost-effective solution (s. Fig. 3a). As mitigation efforts intensify, renewables become the cost-optimal choice for decarbonizing the energy system due to their cost-competitiveness and large-scale availability.*

We discuss, how the temporal hydrogen regulation (mechanism) influences the deployment of renewable capacities and supply from fossil generation (result) in line 128:

> *The cost-optimal solution of the model shows, that increasing hydrogen exports do not necessarily and exclusively require additional renewable capacities. Instead, the electricity demand for hydrogen electrolysis is covered by additional electricity supply from fossil generation. However, if the cost-optimal solution is constrained by the green hydrogen policy, an increase of hydrogen exports goes along with additional renewable electricity capacities as defined in the EU's delegated Act.*

Additionally, we outline the role of the system flexibility necessary for domestic CO_2 mitigation (mechanism) reducing the cost for hydrogen exporters (result) in line 150:

> *At hydrogen export volumes of 25–75 TWh, an increase of domestic CO_2 mitigation up to 40–60% decreases the cost for hydrogen exporters up to -9%. Increasing the domestic CO_2 mitigation requires additional system flexibility through BEV charging, energy storage and transmission capacities (s. Fig. 3a), which benefits hydrogen exporters as well and effectively reduces hydrogen export costs. However, at even higher levels of domestic CO_2 mitigation (70–100%), further renewable energy expansion is required, tapping into sites with less favorable weather conditions in Morocco, which ultimately raises electricity costs (s. Fig. S13d). This effect diminishes at higher export volumes, where the hydrogen export system becomes significantly larger than the domestic electricity system, reducing the relative impact of shared infrastructure as shown in Figure 4b (see also the sensitivity on higher exports in Figure S17b).*

Furthermore, we elaborate on how short-run marginal costs of fossil and renewable generation (mechanism) reduce fossil generation but still play a complementary role (result) in line 182:

> *Stricter hydrogen regulation pushes the price duration curve towards the left, as additional renewable electricity capacities phase out fossil generation, which has higher short-run marginal costs due to fuel costs, compared to renewable electricity. As renewables generate power when available, they reduce the dispatch of fossil generators, whose higher marginal costs make them less competitive in the short term. However, fossil fuels still play a complementary role, stepping in to fill gaps during periods of low renewable generation. This mechanism is fully captured in our capacity expansion and operation model, which accounts for both types of generation in response to price changes and regulatory shifts.*

For the sake of completeness, we have added two examples of explanations carried out in the original manuscript. We outline, how the temporal matching (mechanism) and hence required additional renewable capacities decarbonize the grid emissions (result) in line 193:

> *Figure 6a highlights the energy spillover effect: going beyond the electricity demand of hydrogen electrolysis, renewable capacities installations necessary to meet the temporal matching decarbonize the domestic electricity demand and decrease the grid emissions. This effect is steered by the strictness of temporal matching, hence stricter temporal matching rules enable a further decrease in emissions. The slight increase in total dispatch for annual and monthly matching is balanced by resistive heaters in the heating sector.*

Additionally, we explain how the electrolysis capacity factor (mechanism) influences the price of hydrogen and the resulting consumer cost for hydrogen exporters (result) in line 157:

> *The only relative cost increase observable in the range of our scenarios is observable at low export volumes below 20 TWh. Here, advances in domestic CO_2 mitigation increase the hydrogen export cost by up to 19% compared to no domestic CO_2 mitigation. This cost increase results from lower electrolysis capacity factors compared to the 0% CO_2 mitigation scenario, where we observe high electrolysis capacity factors of above 80% (s. Fig. 13b).*

In sum, we have revised the manuscript substantially to address the concern, that an adequate explanation of the mechanisms driving the results is necessary to provide real-world insights. Furthermore, we would like to emphasize that the added explanations intend to provide universal insights which are not limited to specific calculations. Where limitations exist – such as the availability of specific potentials in Morocco – we have clearly outlined them to ensure transparency (e.g. in line 154):

> *[...] tapping into sites with less favorable weather conditions in Morocco, [...]*

RC: 3. In addition to the preceding methodological concerns, the methodology (i.e., the optimization problem and the solution process employed) is not clearly explained. The authors should formally introduce the model elements, discuss the decision and outcome variables, and clarify the assumptions for the decision variables. Without this information, the methodology lacks transparency.

AR: We are thankful for the remark that the methodology is not explained to a satisfactory extent. We agree, that the manuscript did not state the model equations, outcome variables and assumptions for the decision variables explicitly, and we address this issue in an additional section on the mathematical model formulation in the Supplementary Information:

S1. Mathematical Model Formulation

The core mathematical model formulation of the sector-coupled energy model is based on the European model PyPSA-Eur described in Neumann et al.[8] and the global model PyPSA-Earth and it's sector-coupled extension presented in Abdel-Khalek et al.[18]. The objective of the optimisation is minimizing the annual energy system costs including annualized investment costs and operational expenditures of generation, storage, transmission and conversion infrastructure[8] and is described in Abdel-Khalek et al.[18] as follows:

$$\min_{\substack{G_{n,k},E_{n,k},C_{n,k},F_{l,c}\\ \dot{G}_{n,k,t},\dot{E}_{n,k,t},\dot{C}_{n,k,t},\dot{F}_{l,c,t}}} \overbrace{\sum_{n\in N}\sum_{k\in K} c_k G_{n,k} + \sum_{n\in N}\sum_{k\in K} c_k E_{n,k} + \sum_{n\in N}\sum_{k\in K} c_k C_{n,k} + \sum_{l\in L} c_l F_{l,c}}^{\text{annualized capital costs}}$$
$$\underbrace{+ \sum_{t\in T}\left[w_t\left(\sum_{n\in N}\sum_{k\in K} o_k \dot{G}_{n,k,t} + \sum_{n\in N}\sum_{k\in K} o_k \dot{E}_{n,k,t} + \sum_{n\in N}\sum_{k\in K} o_k \dot{C}_{n,k,t} + \sum_{l\in L} o_l \dot{F}_{l,c,t}\right)\right]}_{\text{marginal costs}} \tag{R1}$$

where the decision variables cover both the optimal capacity of the technology and the optimal hourly dispatch. The model operates on a set of nodes N, set of carriers C, set of technologies K, set of inter-nodal connections L, and a timeframe T, which in this study is a full year. $G_{n,k}$ denotes the optimal generation capacity of an asset of technology k at node n and $\dot{G}_{n,k,t}$ denotes the optimal dispatch of that asset at timestep t. Similarly, the decision variables of storage, converters and energy flow are presented as $E_{n,k}$, $C_{n,k}$ and $F_{l,c}$ respectively where k uniquely identifies the technology for each category, c uniquely identifies the carrier, and l uniquely identifies the inter-nodal connections. The coefficients represent the different cost components: c_k represents the annualized capital cost in technology k while o_k represent the operating costs. The w_t weight variable represents the duration of the timestep t and is decided by the model configuration, in this study it is three-hourly.

In addition to the core mathematical formulation, the model consists of linear constraints on the spatio-temporal availability of renewable energy sources, storage consistency equations, generation, storage, conversion and transmission infrastructure, a limit for CO_2 emissions and transmission and a multi-period linearised optimal power flow (LOPF) formulation. All constraints are described in detail in Neumann et al.[8] resulting in a linear problem (LP).

This added Section in the supplementary material is linked to the main text, in the Section *Methods* describing the sector-coupled model in line 372:

A more detailed mathematical problem formulation is provided in Section S1.

In addition, we would like to emphasize that we are fully aware of the importance of transparency, open data and open source code. To fulfill this aspiration, we have published our full source code, all technology and cost assumptions on GitHub as well as the full model results on Zenodo.

RC: 4. It also appears that the total energy demand per sector is assumed to be constant. This assumption is not convincing since the energy demand per sector is usually affected by factors such as regulations and climate change mitigation targets. There is a substantial amount of research dedicated to estimating future energy demand as economies transition from fossil fuel to renewable electricity as the primary energy source.

AR: We acknowledge the concern regarding energy demand assumptions. We would like to clarify that the assumption of constant demand refers specifically to energy services, not primary or final energy. We agree, that the statement in the original manuscript may lead to confusion in line 667:

> *The inland energy demand per sector remains constant throughout the scenarios presented in the Introduction, [. . .]*

Therefore, we have fully revised this statement (see quotation at the end of this comment).

However, primary and final energy adjust in response to changes in technologies and climate targets in our model. For example, the shift of internal combustion engine cars to battery electric vehicles reduces the oil consumption, as outlined in detail in Figure S7 and discussed in line 117:

> *Battery Electric Vehicle (BEV) diffusion is counterbalancing the demand for oil products [. . .]*

In addition, the final energy demand in the heating sector also shifts from fossil (final) energy demand to electricity with the increased market penetration of heat pumps in dependent on CO_2 mitigation targets as discussed in line 119:

> *Additionally, the electricity demand for air heat pumps increases, supporting the defossilisation in the heating sector [. . .]*

Our methodology and results reflect the exact dynamics the reviewer mentions, where economies shift from fossil fuels to renewable electricity as the primary energy source. We acknowledge the lack of differentiation between primary/final energy and energy services in the statement of the original manuscript and clarify in line 397 tackling the reviewer's concern:

> *The domestic energy services per sector remain constant throughout the scenarios presented in the Introduction, regardless of endogenous hydrogen export volumes or national carbon emission reductions. However, while energy services (e.g., demand for transportation or heating) are fixed, the primary and final energy consumption are subject to CO_2 mitigation targets. To reflect the impact of increasing shares of battery electric vehicles (BEVs) in the Moroccan energy system, the share of BEVs is linked to emission reduction ambitions (s. Fig. S2) from 2% (today's levels) up to a share of 88% at 100% domestic CO_2 mitigation in accordance with Rim et al. [68].*

RC: 5. As discussed above, the analysis in the paper ignores the complexities of market economies. One such complexity is the structure of the market (e.g., is the market highly regulated or liberalized?).

This can substantially affect the extent to which changes in the costs of power generation lead to changes in consumer electricity prices. One finding of the paper is that under hourly temporal regulation, domestic electricity prices do not increase. At the same time, it remains completely unclear what market structure is assumed in the analysis, making it impossible to properly interpret the previous finding.

AR: Thank you for your comment. We acknowledge that the original manuscript did not specify the market structure assumed in our analysis and agree, that the study and the interpretation of its findings substantially benefits from clarifying what market structure is assumed. Our study assumes a liberalized market structure, which allows for dynamic price responses to changes in electricity generation costs.

Morocco's electricity market is partly liberalized and is progressively moving toward a more open and competitive framework, particularly in the renewable energy sector [19]. While the state-owned utility Moroccan National Office for Electricity and Potable Water (Office National de l'Electricité et de l'Eau Potable) (ONEE) historically had a monopoly over generation, transmission, and distribution, this structure has evolved since 2009, when the national energy strategy aimed to increase energy security and integrate renewable energy sources [19, 20]. As part of this liberalization, the Moroccan Agency for Sustainable Energy (MASEN) and Law 13-09 opened up renewable electricity generation to independent power producers (IPPs) through Power Purchase Agreements (PPAs) as outlined in Bentaibi et al. [19]. This allows private actors to generate and sell electricity, primarily in the renewable sector.

The presence of such PPAs is key to the mechanisms analyzed in our study. The introduction of hourly temporal regulation requires additional renewable electricity generation. The resulting, low marginal price excess electricity from these sources penetrates the market, reducing prices for domestic consumers. This dynamic does not rely on the specific market structure alone but rather on the availability of competitive renewable electricity that influences marginal prices.

Furthermore, even in a more regulated market with a vertically integrated utility, this mechanism would hold. Additional renewable installations would still result in times when low-cost excess electricity penetrates the market, hence reducing prices. Thus, the relationship between hourly regulation and consumer electricity prices is relatively independent of the market design. However, we fully recognize, that in a highly regulated market the lower marginal prices resulting from hourly temporal matching might not be passed on to the domestic customers and might be set by the vertically integrated utility in a non-competitive market.

However, not just Morocco is moving towards a fully liberalized electricity market, but this is a trend seen globally [21, 22, 23, 24]. For this reason, we assume a liberalized market structure in our study, providing greater relevance to future scenarios and reflecting long-term trends.

We are thankful for the reviewer to outline the importance of the assumed market structure and fully agree, that the study and the interpretation of its findings substantially benefits from clarifying what market structure is assumed in the introduction in line 96:

> *The model applied in this study assumes a fully liberalized market structure, the possible implications of which are discussed in the Chapter Discussion.*

Furthermore, we outline the implications of the assumed market structure in the limitations in line 330:

> *Another limitation of this study concerns the assumed liberalized market structure in the Moroccan electricity sector, particularly for renewable energy integration. While Morocco is progressively moving toward market liberalization, with increasing penetration of renewables and independent power producers, the electricity market is still partially regulated [48]. In a regulated market structure, the lower marginal prices resulting from hourly temporal matching might not be passed on to the domestic customers and might be set by the vertically integrated utility in a non-competitive market. However, the main findings of the paper still hold, since in both fully liberalized and highly regulated markets, additional renewable installations (due to hourly temporal matching) would still result in times when low-cost excess electricity penetrates the market, hence reducing prices. In a fully competitive market, this benefit would be passed on to domestic electricity consumers. However, not just Morocco is moving towards a fully liberalized electricity market, but this is a trend seen globally [49-52]. For this reason, we assume a liberalized market structure in our study, providing greater relevance to future scenarios and reflecting long-term trends.*

RC: 6. The analysis also implicitly assumes that increasing hydrogen exports and more ambitious mitigation targets will lead to higher installed renewable power capacity. But this is not necessarily the case. The authors should explore the factors influencing such investment decisions more thoroughly. They should also distinguish more clearly between short-term and long-term analysis. For example, the authors state that fossil fuel power generation has "higher marginal costs" compared to renewable energy sources. However, it remains unclear, whether the authors are referring to long-run or short-run marginal costs.

AR: Thank you for your comment. We acknowledge that increasing hydrogen exports and more ambitious mitigation targets do not necessarily lead to higher installed renewable power capacity. Instead, the additional demand can also be covered by higher capacity factors of fossil power generation or by installing additional fossil generators (e.g., gas, coal, oil). In our results in line 219 of the original draft, we observe and explicitly outline that possibility:

> *Without the temporal hydrogen regulation, the additional electricity demand of hydrogen exports is covered by coal power plants and CCGT (by increasing the capacity factor of existing brownfield coal and CCGT capacities) and an expansion of open-cycle gas turbines (OCGT) installation and supply as pointed out in Figure S8.*

However, when applying the Delegated Act's Green Hydrogen definition, hydrogen production from water electrolysis must be covered by renewable electricity (by definition), as investigated in line 216 of the original draft:

> *The deployment of hydrogen exports requires a scale up of onshore wind and solar PV accordingly, as defined by the green hydrogen constraint outlined in [...]*

Following the reviewers recommendation, we have investigated the investment decisions more thoroughly and have added an explanation exploring the relationship between increasing hydrogen exports and additional renewable power capacity in line 128:

> *The cost-optimal solution of the model shows, that increasing hydrogen exports do not necessarily and exclusively require additional renewable capacities. Instead, the electricity demand for hydrogen electrolysis is covered by additional electricity supply from fossil generation. However, if the cost-optimal solution is constrained by the green hydrogen policy, an increase of hydrogen exports goes along with additional renewable electricity capacities as defined in the EU's delegated Act.*

While higher mitigation targets could also be achieved through other technologies, such as fossil electricity generation with CCS or Direct Air Capture, our model is designed to find the cost-optimal solution. In this context, the model identifies additional renewable capacities as the most cost-effective approach and thus deploys them. Due to the cost structure of renewable generation and their potentials in Morocco, they are the least-cost option to achieve CO_2 mitigation targets. We agree with the reviewer to investigate the factors influencing such investments more thoroughly and have added and complementary explanation in line 111:

> *While mitigation targets can (in part) be met with technologies like fossil gas with CCS or Direct Air Capture, the model finds that increasing renewable capacities, particularly solar PV and wind, is the most cost-effective solution (s. Fig. 3a). As mitigation efforts intensify, renewables become the cost-optimal choice for decarbonizing the energy system due to their cost-competitiveness and large-scale availability.*

We agree with the reviewer, that the distinction between short-term and long-term analysis needs to be addressed more clearly. In the original draft, we have used the term *fossil generation with higher marginal costs than renewable [...]* in the description of Figure 5, in line 326 and in line 337. To clarify, the statement refers to short-run marginal costs, where fossil fuels have higher costs due to fuel costs, while renewables have near-zero fuel costs. However, we optimize on long-run marginal costs, which account for capital investments, such as the installation of renewable energy infrastructure. We agree with the reviewer, that those statements are lacking clarity in regard to short-term and long-term costs and therefore specify in line 182:

> *Stricter hydrogen regulation pushes the price duration curve towards the left, as additional renewable electricity capacities phase out fossil generation, which has higher short-run marginal costs due to ongoing fuel costs, compared to renewable electricity.*

Additionally, we specify in the description of Figure 5:

> *[...], as additional renewable electricity capacities phase out fossil generation with higher short-term marginal costs than renewables.*

Furthermore, we specify in line 178:

> *These renewable capacities i.) push out fossil generation with high short-run marginal costs (price spillover) and [...]*

We hope to have fully addressed the reviewers concern on thorough explanation of investment decisions and on a clear distinction between short-term and long-term costs. However, if the reviewer sees additional improvements can be made, we welcome the opportunity to complement our response accordingly.

RC: 7. The study should also provide more information on technical aspects, such as the employed technologies, specifically which types of electrolyzers are used. This is important, as electrolyzers substantially differ in aspects such as cost or capacity factors and may need to be coupled with other electricity storage assets, impacting the cost of hydrogen production.

AR: We agree with the reviewer, that the type of deployed electrolysers is relevant with regards to cost of hydrogen production and system operation in general. Since our presented model optimizes both capacity expansion and operation, the capacity factor of the electrolysers highly depends on the interaction with the energy system and their cost-minimal solution. However, the technical aspects (as part-load behaviour) influence the operation as suggested by the reviewer. We respond by elaborating on the electrolyser type in detail in line 471:

> *For the electrolysis, we use alkaline electrolysers due to their maturity, durability, and lower costs compared to proton exchange membrane electrolysers [45, 89]. Alkaline electrolysers are well-suited for large-scale use, though they have slower start-up times and a more limited operating range than proton exchange membrane electrolysers [89,90], which, in contrast, have shorter cell lifetimes as pointed out in Staffell et al. [89].*

RC: 8. The concept of price spillover also requires further elaboration. Are fossil fuels entirely considered substitutes for renewables, or can they be complementary in some cases? Exploring this relationship is important because it affects how energy markets respond to price changes and policy regulations.

AR: Thank you for your comment. The concept of price spillover in our model refers to the mechanism, that the additional installation of renewable capacities (e.g. due to temporal matching requirements) increasingly penetrates the electricity market. Their low short-term marginal costs (due to near-zero fuel costs) reduces the dispatch of fossil generators. Hence electricity generated by renewables and fossil fuels is interchangeable when meeting demand, but they also act as complementary sources: renewables produce electricity when available, while fossil fuels fill the gaps during periods of low renewable output.

This co-existence reflects real-world energy systems, where fossil fuels are often dispatched to complement variable renewable generation. The interaction between these energy types, and their response to price changes and regulations, is captured in our capacity expansion and operational model. We hope this clarifies the price spillover dynamics in our analysis, and we have added an explanation of these mechanisms in line 182:

> *Stricter hydrogen regulation pushes the price duration curve towards the left, as additional renewable electricity capacities phase out fossil generation, which has higher short-run marginal costs due to fuel costs, compared to renewable electricity. As renewables generate power when available, they reduce the dispatch of fossil generators, whose higher marginal costs make them less competitive in the short term. However, fossil fuels still play a complementary role, stepping in to fill gaps during periods of low renewable generation. This mechanism is fully captured in our capacity expansion and operation model, which accounts for both types of generation in response to price changes and regulatory shifts.*

RC: The paper seems to use the terms "cost" and "price" interchangeably, which is generally not correct.

AR: We agree, *cost* and *price* are not interchangeably. We have addressed it by clarifying the distinction between *costs* and *prices* in the manuscript. Specifically, we now use *costs* for model inputs and system costs, and *prices* to denote endogenous shadow prices from the model. We also define *consumer costs* and *consumer payments* to specify payments by consumers, ensuring consistent terminology throughout. Apart from adjusting the terminology throughout the whole manuscript, we have explained it in line 97:

> *We use costs referring to model inputs and system costs, while prices denote endogenous shadow prices (representing idealized market prices) derived from the model. Payments made by consumers (calculated as energy price times energy volume) are described as (consumer) costs or, alternatively, (consumer) payments to highlight their financial impact.*

RC: The writing style could be less belligerent, and there are several issues and typos (e.g., the authors write on p. 11: "Hourly matching (from 1.1.2030 onwards, until 31.12.2029 monthly)," which is logically incorrect).

AR: We agree with the reviewer, that the writing style is partly belligerent. We have identified various paragraphs and adjusted them accordingly.

Original manuscript:

> *A holistic approach and well-balanced policies is required to steer the energy transition and export ambitions.*

Less belligerent (line 227):

> *A holistic approach and balanced policies are essential to guide the energy transition and support export ambitions effectively.*

Original manuscript:

> *Shifting such issues of acceptance to Morocco by deploying renewable energy and scaling up their hydrogen exports raises moral questions and should be dealt with carefully.*

Less belligerent (line 289):

> *Shifting acceptance challenges to Morocco through renewable energy deployment and expanding hydrogen exports raises important ethical considerations that require thoughtful handling*

Original manuscript:

> *This study shows that the price of electricity highly depends on hydrogen export volumes and temporal hydrogen regulation.*

Less belligerent (line 281):

> *This study demonstrates that electricity prices are closely influenced by hydrogen export volumes and temporal hydrogen regulation.*

Original manuscript:

> *Furthermore, none of these studies looked into exports in depth.*

Less belligerent (line 57):

> *Furthermore, these studies do not explore exports in depth.*

Original manuscript:

> *However, none of them address the complex relationship between all three dimensions.*

Less belligerent (line 60):

> *However, none of them fully address the complex relationship between all three dimensions.*

If the reviewer identifies even more paragraphs with belligerent writing style, we welcome the opportunity to respond again.

Furthermore, we thank the reviewer for pointing out typos and unclear phrasings. We recognize that the original phrasing was unclear. The intended meaning was that the matching is monthly until 31.12.2029 and becomes hourly from 1.1.2030 onwards. The sentence is revised for clarity in line 451:

> *Temporal correlation: Monthly matching until 31.12.2029, and hourly matching from 1.1.2030 onwards [...]*

Furthermore, we have identified and resolved multiple issues and typos.

First, in line 70 (original draft) it is stated that "Numerous studies focus on [...]" but no citations are provided. Since they refer to the stated studies above, The sentence is clarified accordingly to avoid confusions in line 59:

> *While most of the studies presented above focus exclusively on either hydrogen exports, [...]*

Second, in line 26 (original draft) the statement "marginal cost fossil generation" may lead to confusion. The phrase "high marginal cost fossil generation" is grammatically correct, with "high marginal cost" serving as adjectives describing the higher marginal cost of fossil generation in comparison to the lower marginal cost of renewable generation. The structure mirrors common usage, where adjectives modify a noun directly (e.g., "low carbon emissions"). However, we recognize the lack of readability and possible confusion, therefore we propose to rephrase it as "fossil generation with high marginal costs" as edited in line 178:

> *These renewable capacities i.) push out fossil generation with high marginal costs (price spillover) and ii.) provide excess electricity to the domestic electricity system (energy spillover).*

Third, in line 388 (original draft) the statement "It steers the redistribution between hydrogen exports and domestic electricity consumers [...]" is imprecise and logically incorrect, since neither the exports nor the consumers itself are steered, but the respective costs are. We therefore include the term "of costs" in line 217:

> *It steers the redistribution of costs between hydrogen exports and domestic electricity consumers [...]*

Forth, in line 123 (original draft) it should be "...such as low temporal..." rather than just "as low temporal" which is modified in line 86:

> *Shortcomings of studies on highly renewable energy systems in Africa such as low temporal and spatial resolutions as well as the lack of sector-coupled energy models [...]*

Fith, in line 269 (original draft), it is written "[...] is study outlines [...] which is a typo ("this" rather than "is") and is modified in line 135:

> *In an integrated analysis of domestic CO_2 mitigation (dimension 1) and hydrogen exports (dimension 2) this study outlines [...]*

RC: Some figures are not referenced correctly, and some GitHub links do not work properly (e.g., on pages 4, 6, and 10).

AR: We appreciate the thorough review and identification of failed figure and GitHub links. We have fixed the failed figure reference on page 4, which points at Figure 13b. In addition, the full stop at the end of the sentence was missing and is now added in line 160 along with the figure citation:

> *[...], where we observe high electrolysis capacity factors of above 80% (s. Fig. 13b).*

In addition, the figure reference on page 6 was missing. Instead of simply adding the reference, we have decided to rephrase the sentence to improve the clarity, avoid unnecessary references to figures in the supplementary information, and to better link the explanation to the main Figure 6a. The sentence is rephrased in line 190:

> *[...], the electricity supply of coal and OCGT is substantially higher than in the annual, monthly, or hourly case.*

Furthermore, we fixed the link to the (high-level, general) technology-data repository on GitHub on page 10. In addition, we have added the precise version, year and dataset applied in this study as an additional link in line 427:

> *All technology and cost assumptions are based on the year 2030, taken from the technology-data repository. This study applies version 0.4.0 of the repository, which is available at technology-data v0.4.0.*

RC: The referencing style should be more consistent.

AR: We are thankful for the comment and highly support a consistent referencing style. Therefore, we have identified and resolved multiple inconsistencies. First of all, in the original draft line 160, a citation was used for the whole paragraph. While adding the citation for the whole paragraph behind the point of the last sentence of the paragraph is a valid solution for most disciplines and journals, we have decided to integrate the citation wherever necessary within the paragraph to prevent possible confusions and have a consistent referencing style. The revised paragraph starts in line 13:

> *Figure 1 shows the hydrogen strategy of Morocco [9], including national and export demands. By 2030, the total hydrogen demand adds up to 13.9 TWh/a and ramps up to 67.9 TWh/a in 2040 and 153.9 TWh/a in 2050, clearly listing higher demands for export than for domestic use. The hydrogen generation is backed by 5.2 GW of RE in 2030, 23 GW in 2040, and 57.4 GW in 2050 in the export sector [9]. To cover the national demand of hydrogen, a Renewable Energy deployment of 1.6 GW in 2030, 7.0 GW in 2040 and 10.3 GW in 2050 is planned in Morocco's Hydrogen Strategy [9].*

Second, in line 678 (original draft) the number was cited directly without adding the author's name in front, which is resolved in line 406:

> *The sector-coupled energy model follows the data pipeline of Parzen et al. [58] by incorporating solar PV, onshore wind and hydro power using the open source package Atlite [69].*

Third, the same appeared in the original draft line 707, which is resolved in line 423:

> *Following the workflow of Parzen et al. [58], Figure [. . .]*

Forth, the same appeared in the original draft line 735 and 737, which is resolved in line 442:

> *[. . .] due to treatment chemicals and high salinity as discussed by Thomann et al. [84], Dresp et al. [85] and Tonelli et al. [86], highlighting the importance of a careful site selection of desalination plants. A minimum distance of four kilometers from marine protected areas is recommended in Thomann et al. [84].*

We are confident to have caught all inconsistent referencing styles. However, if the reviewer has identified further inconsistencies, we welcome the opportunity to respond again.

Reviewer #4

RC: (Remarks on code availability) I reviewed the code available on GitHub but did not perform a comprehensive analysis to rerun it and verify the reproducibility of the results. Our review primarily focused on the paper's content and its contributions rather than on reproducing the results using the code.

AR: Thank you for reviewing the code on GitHub. We appreciate your feedback and understand that the focus of the review was on the paper's content and contributions rather than reproducing the results. We tested the installation and running of the code from scratch to ensure reproducibility. The source code, input data and results have been made available to ensure transparency and reproducibility, and we are happy to assist with any further inquiries or clarifications regarding its use.

References

[1] E. Zeyen, I. Riepin, T. Brown, Temporal regulation of renewable supply for electrolytic hydrogen, Environmental Research Letters 19 (2) (2024) 024034. doi:10.1088/1748-9326/ad2239.

[2] M. A. Giovanniello, A. N. Cybulsky, T. Schittekatte, D. S. Mallapragada, The influence of additionality and time-matching requirements on the emissions from grid-connected hydrogen production, Nature Energy (2024) 1–11doi:10.1038/s41560-023-01435-0.
URL https://www.nature.com/articles/s41560-023-01435-0

[3] V. P. Müller, W. Eichhammer, D. van Vuuren, Paving the way: Analysing energy transition pathways and green hydrogen exports in developing countries – The case of Algeria, International Journal of Hydrogen Energy 67 (2024) 240–250. doi:10.1016/j.ijhydene.2024.04.153.
URL https://www.sciencedirect.com/science/article/pii/S0360319924014265

[4] E. Zeyen, M. Victoria, T. Brown, Endogenous learning for green hydrogen in a sector-coupled energy model for Europe, Nature Communications 14 (1) (Jun. 2023). doi:10.1038/s41467-023-39397-2.

[5] M. Victoria, K. Zhu, T. Brown, G. B. Andresen, M. Greiner, Early decarbonisation of the European energy system pays off, Nature Communications 11 (1) (Dec. 2020). doi:10.1038/s41467-020-20015-4.

[6] L. Franken, A. Hackett, J. Lizana, I. Riepin, R. Jenkinson, A. Lyden, L. Yu, D. Friedrich, Power system benefits of simultaneous domestic transport and heating demand flexibility in Great Britain's energy transition, Applied Energy 377 (2025) 124522. doi:10.1016/j.apenergy.2024.124522.
URL https://www.sciencedirect.com/science/article/pii/S0306261924019056

[7] F. Neumann, J. Hampp, T. Brown, Energy Imports and Infrastructure in a Carbon-Neutral European Energy System (Apr. 2024). arXiv:2404.03927.
URL http://arxiv.org/abs/2404.03927

[8] F. Neumann, E. Zeyen, M. Victoria, T. Brown, The Potential Role of a Hydrogen Network in Europe, Joule 7 (8) (2023) 1793–1817. arXiv:2207.05816, doi:10.1016/j.joule.2023.06.016.
URL http://arxiv.org/abs/2207.05816

[9] F. Neumann, V. Hagenmeyer, T. Brown, Assessments of linear power flow and transmission loss approximations in coordinated capacity expansion problems, Applied Energy 314 (2022) 118859. doi:10.1016/j.apenergy.2022.118859.

[10] F. Neumann, T. Brown, The near-optimal feasible space of a renewable power system model, Electric Power Systems Research 190 (2021) 106690. doi:10.1016/j.epsr.2020.106690.

[11] M. Parzen, H. Abdel-Khalek, E. Fedotova, M. Mahmood, M. M. Frysztacki, J. Hampp, L. Franken, L. Schumm, F. Neumann, D. Poli, A. Kiprakis, D. Fioriti, PyPSA-Earth. A new global open energy system optimization model demonstrated in Africa, Applied Energy 341 (2023) 121096. doi:10.1016/j.apenergy.2023.121096.

[12] J. Hörsch, F. Hofmann, D. Schlachtberger, T. Brown, PyPSA-Eur: An open optimisation model of the European transmission system, Energy Strategy Reviews 22 (2018) 207–215. doi:10.1016/j.esr.2018.08.012.

[13] S. Collins, P. Deane, B. Ó. Gallachóir, S. Pfenninger, I. Staffell, Impacts of inter-annual wind and solar variations on the european power system, Joule 2 (10) (2018) 2076–2090. doi:10.1016/j.joule.2018.06.020.

[14] A. S. Oyewo, S. Sterl, S. Khalili, C. Breyer, Highly renewable energy systems in Africa: Rationale, research, and recommendations, Joule 7 (7) (2023) 1437–1470. doi:10.1016/j.joule.2023.06.004.

[15] A. S. Oyewo, G. Lopez, M. ElSayed, T. Galimova, C. Breyer, Power-to-X Economy: Green e-hydrogen, e-fuels, e-chemicals, and e-materials opportunities in Africa, Energy Reports 12 (2024) 2026–2048. doi:10.1016/j.egyr.2024.08.011.
URL https://www.sciencedirect.com/science/article/pii/S2352484724005055

[16] F. Bauer, M. Sterner, Impacts of lifestyle changes on energy demand and greenhouse gas emissions in Germany, Renewable and Sustainable Energy Reviews 207 (2025) 114944. doi:10.1016/j.rser.2024.114944.
URL https://www.sciencedirect.com/science/article/pii/S1364032124006701

[17] I. E. Agency, Net zero by 2050. A roadmap for theGlobal energy sector, Tech. rep., INTERNATIONAL ENERGY AGENCY (2021).

[18] H. Abdel-Khalek, L. Schumm, E. Jalbout, M. Parzen, C. Schauß, D. Fioriti, Sector-Coupled Pypsa-Earth, a Global Open-Source Multi-Energy System Model (Feb. 2024). doi:10.2139/ssrn.4743242.
URL https://papers.ssrn.com/abstract=4743242

[19] W. Bentaibi, B. Pape, G. L. Nouel, Electricity regulation in Morocco: Overview (2021).

[20] B. Brand, J. Zingerle, The renewable energy targets of the Maghreb countries: Impact on electricity supply and conventional power markets, Energy Policy 39 (8) (2011) 4411–4419. doi:10.1016/j.enpol.2010.10.010.
URL https://linkinghub.elsevier.com/retrieve/pii/S0301421510007603

[21] N. Fatras, Z. Ma, H. Duan, B. N. Jørgensen, A systematic review of electricity market liberalisation and its alignment with industrial consumer participation: A comparison between the Nordics and China, Renewable and Sustainable Energy Reviews 167 (2022) 112793.

doi:10.1016/j.rser.2022.112793.
URL https://www.sciencedirect.com/science/article/pii/S1364032122006773

[22] R. Faia, F. Lezama, J. Soares, T. Pinto, Z. Vale, Local electricity markets: A review on benefits, barriers, current trends and future perspectives, Renewable and Sustainable Energy Reviews 190 (2024) 114006. doi:10.1016/j.rser.2023.114006.
URL https://www.sciencedirect.com/science/article/pii/S136403212300864X

[23] C. Xu, F. Wen, I. Palu, Electricity market regulation: Global status, development trend, and prospect in China, Energy Conversion and Economics 1 (3) (2020) 151–170. doi:10.1049/enc2.12020.
URL https://onlinelibrary.wiley.com/doi/abs/10.1049/enc2.12020

[24] B. Fattouh, A. Sen, R. Poudineh, Electricity Markets in the Resource-Rich Countries of the MENA: Adapting for the Transition Era, Economics of Energy and Environmental Policy 10 (1) (Jan. 2021).
URL https://eprints.soas.ac.uk/32789/

Author Response to Reviews of

The impact of temporal hydrogen regulation on hydrogen exporters and their domestic energy transition

Leon Schumm, Hazem Abdel-Khalek, Tom Brown, Falko Ueckerdt, Michael Sterner, Max Parzen, Davide Fioriti
Nature Communications
Revision #2: February 19, 2025

RC: Reviewer Comment, AR: Author Response, ▌ *Manuscript text*

We thank the reviewers for their constructive comments and acknowledge the time and effort they have spent in assessing our work. We have revised the paper based on the feedback from the reviewers and hope that we can adequately address their primary concerns.

To follow the revisions made, we have highlighted differences compared with the previous submitted version of the paper in blue and red text in an attached file. Figure numbers in this response letter refer to the numbering in the revised version of the manuscript.

Reviewer #1

RC: I commend the authors for comprehensively addressing my critiques and those of the other reviewers, and I am ready to recommend the paper for acceptance.

AR: We sincerely appreciate your thoughtful feedback throughout the review process and are delighted to hear that our revisions have addressed your critiques. We are grateful for your recommendation for acceptance.

Reviewer #2

RC: Most of my concerns were addressed appropriately, and the authors have added valuable background information, specifications, and refinements to the methodology description. Additionally, the inclusion of raw data provides a more detailed understanding of the work. However, my main concern remains unresolved. As noted in my previous comments, Figure 4 is prone to misinterpretation, which previously led to incorrect conclusions being drawn, as acknowledged in the first revision. While the problematic conclusions have been adequately removed and the

integration of transformation pathways has improved, Figure 4 itself has not changed. Reviewer #1 also highlighted that the chosen visualization does not effectively represent the data, as the normalization approach appears unintuitive and misaligned with the plot type. Even if one understands its limitations, it is not entirely clear what interpretations the figure allows, and what might be misinterpretations. After further consideration, I think I understand why you chose this approach to normalization and the corresponding plots. For instance, in Figure 4(a), are you aiming to show the effect of external stakeholder interests—specifically hydrogen exports—on the local benefits of electricity costs? If so, it would make sense to examine the partial derivative of electricity costs with respect to hydrogen exports while holding CO_2 mitigation constant as you did. Similarly, for Figure 4(b), are you illustrating how internal stakeholder interests in CO_2 reduction result in benefits for external stakeholders through reduced hydrogen export costs? If this is your intention, then the plot becomes more logical. This storyline, however, does not come across clearly in the current manuscript. I recommend revising lines 139–147 and 148–165 to emphasize this narrative. With these updates, your analysis would better support the adapted conclusions presented in line 166 and beyond. Additionally, to help readers avoid misinterpreting the figure, I suggest incorporating visual aids to guide interpretation. As this is hard to describe, I have included a suggestion in the attached files (Suggestion_Figure_4.png) to illustrate what would have helped me interpret the figure correctly from the outset. If my interpretation of Figure 4 does not align with your intent, this indicates residual ambiguity that should be even more addressed before publication.

AR: We are pleased to hear that most of the concerns have been addressed appropriately and that the additional background information, methodological refinements, and raw data have improved the manuscript. We are thankful for the further consideration of Figure 4. We fully agree with your interpretation:

- Figure 4a aims to show the effect of external stakeholder interests—specifically hydrogen exports—on the local benefits of electricity costs and
- Figure 4b illustrates how internal stakeholder interests in CO_2 reduction result in benefits for external stakeholders through reduced hydrogen export costs.

This is precisely the intended message of Figure 4. We also appreciate your suggestion to incorporate visual aids to improve clarity. We have implemented them with only minor adjustments in terminology to ensure consistency with the rest of the manuscript. In addition, we have revised the description of Figure 4a:

> *Impact of hydrogen exports on domestic interests: Relative cost of electricity for domestic consumers (normalized to 1 TWh/a hydrogen export)*

and Figure 4b:

> *Impact of domestic CO_2 mitigation on hydrogen exporter interests: Relative cost of hydrogen for exporters (normalized to 0% domestic CO_2 mitigation)*

emphasizing the main purpose of these Figures to improve comprehensibility.

Furthermore, we have adapted the storyline in lines 139–147 and 148–165 to emphasize this narrative more clearly. We have done so by adding an introductory sentence highlighting the main message of each Figure, revising the explanation of negative values and overhauling the whole description conveying the main message more precisely. In line with the remarks of an other reviewer's comment, we have extended our analysis on the underlying mechanisms driving the results. The revised text now reads in lines 140-177:

> *First, Figure 4a illustrates how domestic electricity consumers benefit from a stronger hydrogen export industry at different decarbonization targets. The costs are normalized to the cost level at 1 TWh/a hydrogen export for each domestic CO_2 mitigation level, showing the relative impact of hydrogen exports on domestic electricity costs. Negative values indicate that hydrogen exports reduce average electricity costs for domestic consumers. Figure 4a shows, that hydrogen exports decrease the relative domestic electricity costs by up to 45%, especially at mitigation levels below 40% and exports above 50 TWh. In these ranges, the domestic electricity consumers profit from excess green electricity originating from additional renewable energy capacities required for hydrogen export. These renewable capacities i.) push out fossil generation with high short-run marginal costs (price spillover) and ii.) provide excess electricity to the domestic electricity system (energy spillover), elaborated in detail in the section on the impacts of hydrogen regulation on electricity prices and in the Supplementary Fig. 12, Supplementary Fig. 13 and Supplementary Fig. 14. However, as the domestic electricity system undergoes deeper decarbonization (domestic CO_2 mitigation above 50–60%), the cost-reducing effect of hydrogen exports weakens. Here, the impact of additional renewable electricity capacities is only marginal, since the electricity system is already highly or fully renewable electricity based and neither price spillover nor energy spillover are effective as outlined in the section Discussion. The only scenario in which an increase of hydrogen export leads to higher domestic electricity costs is at a low CO_2 mitigation level of 10% and exports of 20 TWh. This effect arises due to the initial increase in electricity demand from hydrogen electrolysis, which raises electricity cost, as seen in Supplementary Fig. 6a (without temporal regulation). Without regulation, this cost increase continues with higher exports. However, with regulation, the cost-reducing effect of hydrogen exports eventually dominates through the effect of energy and price spillovers, as discussed in the section on impacts of hydrogen regulation on electricity prices, mitigating further cost increases and significantly decreasing the cost for domestic electricity consumers.*

> *Second, Figure 4b shows how hydrogen exporters benefit from domestic CO_2 mitigation. The costs are normalized to the cost level at 0% CO_2 mitigation for each hydrogen export volume, displaying the relative impact of domestic CO_2 mitigation on hydrogen export costs. At hydrogen export volumes of 25–75 TWh, increasing domestic CO_2 mitigation up to 40–60% reduces hydrogen export costs by up to -9%. This cost reduction is driven by improvements in system flexibility – such as BEV charging, energy storage, and expanded transmission capacities (s. Fig. 3a) – which also benefit hydrogen exporters. However, at even higher domestic CO_2 mitigation levels (70–100%), further renewable expansion is required, tapping into sites with less favorable weather conditions in Morocco. This ultimately raises electricity costs, as seen in Supplementary Fig. 7d. At higher hydrogen export volumes, this effect diminishes. As the hydrogen export system grows significantly larger than the domestic electricity system, shared infrastructure effects become less relevant as shown in Figure 4b and in the sensitivity analysis for higher exports in Supplementary Fig. 10b. The only scenario where domestic CO_2 mitigation leads to a notable increase in hydrogen export costs occurs at low export volumes below 20 TWh. Here, advances in domestic CO_2 mitigation increase hydrogen export costs by up to 19% compared to a scenario without domestic CO_2 mitigation. This cost increase results from lower electrolysis capacity factors compared to the 0% CO_2 mitigation scenario, where we observe high electrolysis capacity factors of above 80% (see Supplementary Fig. 7b). The top-left region of Figure 4b indicates that at certain export levels, hydrogen export costs become largely independent of domestic CO_2 mitigation. This is driven by temporal hydrogen regulation further investigated in the section on the impacts of hydrogen regulation on electricity prices. Here, hydrogen export infrastructure decouples from the domestic electricity system, the cost is increasingly independent of domestic CO_2 mitigation. When hydrogen exports reach twice the size of domestic electricity demand (s. Fig. 4b), the influence of the domestic electricity system on hydrogen export costs becomes negligible.*

We believe these improvements enhance the figure's interpretability and alignment with the adapted storyline in lines 140–158 and 159–177. Thank you again for your valuable (visual) input.

RC: The code is made available to a high degree. It includes a well-documented code package as well as installation guides and can therefore be reproduced. This is true for the code of the optimization model as well as the conducted result analysis. Also the input data as well as the result data are available.

We appreciate your positive feedback regarding the availability and reproducibility of our code, input data, and results. We are pleased that our updates to the manuscript and repositories have addressed your previous concerns.

Reviewer #3

RC: As pointed out before, the analysis takes the perspective of a central planner who effectively makes all decisions in the model economy. The authors argue that this approach is common in

energy system modeling and can provide some real-world insights, even if it is not reflective of most market economies. I agree with this. However, the authors also state that they assume a liberalized market. This contradicts the modeling approach! Either the analysis assumes a central planner seeking to minimize the total cost of the energy system to meet a given energy demand. Or the analysis assumes a liberalized market in which individual market participants maximize their profits in equilibrium. Both approaches require specific assumptions. Importantly, the results of the two modeling approaches are often fundamentally different!

AR: Thank you for your comment. We apologize for causing a misunderstanding and want to clarify: We do not model liberalized markets. We revised the text, cite economic theory and list the conditions for the central planner results to being equivalent with the decentral market solution, while we also discuss - in line with your argument - that these conditions are often not fully valid in real-world liberalized markets, where market participants maximize their profits in equilibrium, resulting in different outcomes compared to an ideal-world central planner: When markets are complete and competitive, i.e. with perfect information, free entry and exit, many participants and no transaction costs, externalities or market failures, the equilibrium reached by price-taking profit-maximizing firms and utility-maximizing consumers coincides with the solution a benevolent central planner would choose to minimize total system costs (or maximize total welfare if we include the combined utility of all the perfectly inelastic demand). This is the First Fundamental Welfare Theorem and we have now been clearer about the conditions for it to hold. In real-world liberalized markets these assumptions are not always valid: some actors are missing full information, some generators have market power, and there are various government interventions that move us away from equilibrium, for example.

We clarify this in the introduction in lines 96-98:

> *The model assumes a central planner's perspective, with the results being equivalent to a decentral market solution under the conditions outlined in the section Discussion.*

Furthermore, we have fully revised the paragraph on the market structure, now clearly stating that we do not model liberalized markets. Instead we point out, under which conditions the central planner results are being equivalent with the decentral market solution in lines 342-354:

> *Another limitation of this study concerns the market structure in the Moroccan electricity sector, particularly regarding the integration of renewable energy. While Morocco is progressively moving toward market liberalization, with increasing penetration of renewable electricity and independent power producers, the electricity market is still partially regulated [49]. However, we want to clarify that our study does not model a liberalized market, in which individual market participants maximize their profits in equilibrium. Instead, we assume a central planner's perspective based on economic theory, where market outcomes are derived under the assumption of complete and competitive markets. In a perfectly competitive market with perfect information, free entry and exit, many participants, and no transaction costs or market failures, the equilibrium reached by price-taking firms and utility-maximizing consumers coincides with the solution a benevolent central planner would choose to minimize total system costs. This is consistent with the First Fundamental Welfare Theorem [50,51]. However, in real-world liberalized markets, these assumptions are often not fully valid: some actors may lack full information, some generators may have market power, and various government interventions can move us away from equilibrium. Despite the fact that the conditions for equivalence between central planner and market outcomes are not fully satisfied in practice, we argue that our study remains relevant for future scenarios, where these conditions may increasingly hold as markets evolve towards liberalization globally [52-55].*

RC: In response to point 5 of my earlier comments, the authors write: "low marginal price excess electricity from these [renewable] sources penetrates the market, reducing prices for domestic consumers. This dynamic does not rely on the specific market structure alone but rather on the availability of competitive renewable electricity that influences marginal prices. [...] [E]ven in a more regulated market with a vertically integrated utility, this mechanism would hold. [...] Thus, the relationship between hourly regulation and consumer electricity prices is relatively independent of the market design." Again, the authors confuse economic concepts. High renewable power generation in some hours suppresses prices in the wholesale electricity market in those hours. But whether this temporary price suppression in the wholesale market results in lower electricity prices for residential consumers depends on many factors, including the market structure, market regulation, retail electricity tariffs, and wholesale market prices during hours of low renewable power generation. So-called "hourly regulation" may lead to more renewable energy deployment and thus to more frequent and larger suppressions of wholesale market prices. Yet, the relationship between this hourly regulation and consumer electricity prices is generally not independent of the market design. This issue is crucial because the author's assumptions about the market design remain unclear. At the same time, the main results of the paper concern the relationship between this hourly regulation and consumer electricity prices.

AR: We agree that this statement is highly dependent on the market design. In markets where consumers have dynamic tariffs, like many consumers in Norway or Spain, this effect will be there because the market price is passed through to consumers on an hourly basis. Our idealised market setting essentially assumes that all consumers have dynamic tariffs and see the hourly market price fluctuations. In other markets where monthly or yearly baseload future prices are decisive for setting the production part of electricity rates, like Germany, this effect will be indirectly there

(via the effect of hourly price reductions on the average price), but not all markets have these mechanisms to pass through price reductions. We have revised the manuscript and emphasize in line 354:

> *In this context, we emphasize that the effect of temporal hydrogen regulation on domestic electricity consumers observed in the section results is only valid where the market design is such that market prices are passed through to electricity consumers.*

RC: While the authors now better explain some mechanisms, they remain silent on others. For example, Figure 4a shows that the output variable "Relative domestic electricity cost" is strongly non-monotonic in the two input variables plotted on the x- and y-axes of the graph. Similarly, the output variable "Relative export hydrogen cost" in Figure 4b appears to be generally non-monotonic in the input variables plotted on the x- and y-axes of the graph. Both graphs beg the question: why? What's going on here?

AR: We appreciate the acknowledgment that we have improved the explanation of some mechanisms and agree with the observation that the output variables of Figures 4a and 4b exhibit non-monotonic behavior. We have revised the manuscript and now provide a more detailed explanation of the mechanisms driving the results in Figure 4a and 4b in lines 140-177:

First, Figure 4a illustrates how domestic electricity consumers benefit from a stronger hydrogen export industry at different decarbonization targets. The costs are normalized to the cost level at 1 TWh/a hydrogen export for each domestic CO_2 mitigation level, showing the relative impact of hydrogen exports on domestic electricity costs. Negative values indicate that hydrogen exports reduce average electricity costs for domestic consumers. Figure 4a shows, that hydrogen exports decrease the relative domestic electricity costs by up to 45%, especially at mitigation levels below 40% and exports above 50 TWh. In these ranges, the domestic electricity consumers profit from excess green electricity originating from additional renewable energy capacities required for hydrogen export. These renewable capacities i.) push out fossil generation with high short-run marginal costs (price spillover) and ii.) provide excess electricity to the domestic electricity system (energy spillover), elaborated in detail in the section on the impacts of hydrogen regulation on electricity prices and in the Supplementary Fig. 12, Supplementary Fig. 13 and Supplementary Fig. 14. However, as the domestic electricity system undergoes deeper decarbonization (domestic CO_2 mitigation above 50–60%), the cost-reducing effect of hydrogen exports weakens. Here, the impact of additional renewable electricity capacities is only marginal, since the electricity system is already highly or fully renewable electricity based and neither price spillover nor energy spillover are effective as outlined in the section Discussion. The only scenario in which an increase of hydrogen export leads to higher domestic electricity costs is at a low CO_2 mitigation level of 10% and exports of 20 TWh. This effect arises due to the initial increase in electricity demand from hydrogen electrolysis, which raises electricity cost, as seen in Supplementary Fig. 6a (without temporal regulation). Without regulation, this cost increase continues with higher exports. However, with regulation, the cost-reducing effect of hydrogen exports eventually dominates through the effect of energy and price spillovers, as discussed in the section on impacts of hydrogen regulation on electricity prices, mitigating further cost increases and significantly decreasing the cost for domestic electricity consumers.

> *Second, Figure 4b shows how hydrogen exporters benefit from domestic CO_2 mitigation. The costs are normalized to the cost level at 0% CO_2 mitigation for each hydrogen export volume, displaying the relative impact of domestic CO_2 mitigation on hydrogen export costs. At hydrogen export volumes of 25–75 TWh, increasing domestic CO_2 mitigation up to 40–60% reduces hydrogen export costs by up to -9%. This cost reduction is driven by improvements in system flexibility – such as BEV charging, energy storage, and expanded transmission capacities (s. Fig. 3a) – which also benefit hydrogen exporters. However, at even higher domestic CO_2 mitigation levels (70-–100%), further renewable expansion is required, tapping into sites with less favorable weather conditions in Morocco. This ultimately raises electricity costs, as seen in Supplementary Fig. 7d. At higher hydrogen export volumes, this effect diminishes. As the hydrogen export system grows significantly larger than the domestic electricity system, shared infrastructure effects become less relevant as shown in Figure 4b and in the sensitivity analysis for higher exports in Supplementary Fig. 10b. The only scenario where domestic CO_2 mitigation leads to a notable increase in hydrogen export costs occurs at low export volumes below 20 TWh. Here, advances in domestic CO_2 mitigation increase hydrogen export costs by up to 19% compared to a scenario without domestic CO_2 mitigation. This cost increase results from lower electrolysis capacity factors compared to the 0% CO_2 mitigation scenario, where we observe high electrolysis capacity factors of above 80% (see Supplementary Fig. 7b). The top-left region of Figure 4b indicates that at certain export levels, hydrogen export costs become largely independent of domestic CO_2 mitigation. This is driven by temporal hydrogen regulation further investigated in the section on the impacts of hydrogen regulation on electricity prices. Here, hydrogen export infrastructure decouples from the domestic electricity system, the cost is increasingly independent of domestic CO_2 mitigation. When hydrogen exports reach twice the size of domestic electricity demand (s. Fig. 4b), the influence of the domestic electricity system on hydrogen export costs becomes negligible.*

We hope that the revised explanation clarifies the mechanisms behind the non-monotonic behavior observed in Figures 4a and 4b. By detailing the interactions between hydrogen exports and domestic CO_2 mitigation, including its underlying effects, we aim to address the concerns and provide a more comprehensive understanding of the underlying dynamics. We appreciate the reviewer's feedback, which has helped improve the clarity of our analysis.

RC: In addition to the preceding concerns, there are several minor issues:

- l. 58: "drices" should read "drives"
- l. 216: I would suggest deleting the part "leading the system closer to an equilibrium where marginal prices match the optimization's objective function" because it is unclear what is meant in economic terms.
- Figure 7 shows a line chart for findings that change by category. This depiction is highly misleading as it suggests that the linear interpolations between the categories are actual results. I would strongly suggest providing some other depiction of the findings that avoid this issue.

AR: We are thankful for the further suggestions. We have corrected the typo and now line 55 reads:

> *...highlighting how additionality drives the emissions impact of temporal hydrogen regulation.*

Furthermore, we have deleted the part "leading the system closer to an equilibrium where marginal prices match the optimization's objective function." to enhance readability and avoid confusion for the reader. The revised sentence in line 277 now reads:

> *This observation also holds if the coal capacity is decreased under high decarbonization scenarios where fossil fuel dispatch is minimized.*

In addition, we appreciate the feedback regarding the potential for misinterpretation in Figure 7, which suggests that the linear interpolations are actual results. In response, we have fully revised Figure 7 (and the according sensitivity analysis in Figure S11) to avoid any misleading interpolation between categories. The updated figure now uses violin plots, which more accurately represent the distribution of mitigation-export scenarios across different transformation speeds (categories). Additionally, we have revised the caption of Figure 7 and S11:

> *Relative change of electricity (s. Fig. 7a) and hydrogen consumer cost (s. Fig. 7b) depending on the temporal hydrogen regulation. Violin plots indicate median (white dot), interquartile range (thick black bar), and 1.5x interquartile range (thin black bar), using kernel density estimation to show the distribution shape of the mitigation-export scenarios, grouped into different speeds of transformation (1,2,3). [...]*

and the corresponding description in the main text to reflect these changes in lines 248 onwards:

> *In the group of Balanced exports and CO_2 mitigation the effect of temporal hydrogen regulation is less dominant. Hourly matching reduces the cost for domestic electricity consumers by up to -31% (s. Fig. 7a). In only two single mitigation-export scenarios the annual or monthly matching decreases the cost for domestic electricity, whereas all other combinations of the Balanced exports and CO_2 mitigation group are only sensitive to the strictest – hourly matching – hydrogen regulation. The cost increase for hydrogen exporters (s. Fig. 7b) is minimal, the median of the Balanced exports and CO_2 mitigation scenarios indicates a 1% increase. In only two scenarios, hourly matching regulation increases the hydrogen cost above 7% (above 11% across all groups) compared to no temporal hydrogen regulation. This is the case for 0% CO_2 mitigation and 1 and 20 TWh/a export scenarios.*

We believe this revised depiction more clearly communicates the findings and avoids the issue of interpolation between categories.

Reviewer #4

RC: I co-reviewed this manuscript with one of the reviewers who provided the listed reports. This is part of the Nature Communications initiative to facilitate training in peer review and to provide appropriate recognition for Early Career Researchers who co-review manuscripts.

AR: We appreciate the efforts of both reviewers in providing detailed and constructive feedback. We also value the Nature Communications initiative supporting Early Career Researchers in peer review, which we believe strengthens the scientific community.

Author Response to Reviews of

The impact of temporal hydrogen regulation on hydrogen exporters and their domestic energy transition

Leon Schumm, Hazem Abdel-Khalek, Tom Brown, Falko Ueckerdt, Michael Sterner, Max Parzen, Davide Fioriti
Nature Communications
Revision #3: June 24, 2025

RC: Reviewer Comment, AR: Author Response, ▌ *Manuscript text*

We thank the reviewers for their constructive comments and acknowledge the time and effort they have spent in assessing our work. We have revised the paper based on the feedback from the reviewers and hope that we can adequately address their primary concerns.

To follow the revisions made, we have highlighted differences compared with the previous submitted version of the paper in blue and red text in an attached file. Figure and line numbers in this response letter refer to the numbering in the revised version of the manuscript.

Reviewer #2

RC: The authors have successfully addressed my final remarks. The paper now makes a valuable contribution to the scientific community by introducing a novel approach to evaluating export energy systems. I have no further comments.

AR: We sincerely appreciate your thoughtful feedback throughout the review process and are delighted to hear that our revisions have addressed your final remarks. We are grateful for your assessment.

Reviewer #3

RC: The authors have further revised the manuscript and have addressed most of my earlier concerns. Yet, several items require further clarification.

AR: Thank you for your continued engagement and for acknowledging our revisions. We appreciate your detailed review and will address the remaining points requiring clarification in the following responses.

RC: 1. In response to earlier comments, the authors have clarified that their analysis takes the perspective of a central planner. To further avoid confusion, I would suggest deleting the following two sentences:

- a. l. 96 ff.: "The model assumes a central planner's perspective, with the results being equivalent to a decentral market solution under the conditions outlined in the section Discussion."
- b. l. 366 f.: delete the part "based on economic theory, where market outcomes are derived under the assumption of complete and competitive markets."

AR: Thank you for your suggestion. To improve clarity and avoid any potential confusion regarding our modeling perspective, we have followed your recommendation and deleted both sentences as suggested.

RC: 2. In the paper, the authors generally speak of the "costs for domestic electricity consumers." Hereby, they seem to mean the total cost of the electricity system, not the electricity bills consumers pay. The former cost metric follows directly from the analysis. The latter cost metric would require further assumptions, for instance, about the market structure and the cost pass-through from utilities to end consumers. Since this can be easily misunderstood, I would ask the authors to clarify the language, possibly by avoiding the term "costs for domestic electricity consumers."

AR: Thank you for this important clarification. We agree that the term "costs for domestic electricity consumers" can be misleading, as it may be interpreted as referring to actual electricity bills—which would include taxes, regulated tariffs, and cost pass-through mechanisms not captured by our model.

The market-based costs for domestic electricity consumers do not represent the total system cost, nor do they correspond to real-world electricity bills. Rather, they capture the share of modeled system expenditures borne by domestic electricity consumers based on their consumption and market-based pricing. This is distinct from other consumer groups—such as electricity demand for hydrogen electrolysis—which also contribute to total system costs through their electricity demand.

To avoid this confusion, we have revised the terminology throughout the manuscript and refrain from using the term. Instead, we now refer to "market-based costs for domestic electricity consumers." This terms better reflect what we actually quantify: The costs for domestic electricity consumers based on (idealized) market prices, calculated as the product of three-hourly nodal electricity prices (in €/MWh) and electricity consumption (in MWh) at each timestep and node. These prices are model-derived shadow prices representing idealized market prices.

To clarify this distinction explicitly, we have revised the paragraph in the Introduction that defines the use of costs, prices, and consumer payments. It now reads in lines 98 ff.:

> *We use costs referring to model inputs and system costs, while prices denote endogenous shadow prices (representing idealized market prices) derived from the model. Payments made by consumers (calculated as energy price times energy volume) are described as market-based (consumer) costs based on idealized market prices or alternatively (consumer) payments to highlight their financial impact. These payments only translate into one component of consumer electricity bills if whole-sale electricity prices are passed through to the retail tariff. Additional components in retail electricity prices depend on region-specific regulation and are not included here.*

We have also reviewed the terminology throughout the manuscript (see lines 94, 96, 140, 160, 247, 255, 264, captions of Figures 4 and 6), Abstract and Supplementary Information (captions of Supplementary Figures 6 and 17). We hope that this revised terminology addresses the concern. However, we are happy to consider alternative phrasing if the reviewer has a preferred term that reflects the market-based costs for domestic electricity consumers.

RC: 3. The manuscript would also benefit from a more precise framing regarding whether the analysis is fundamentally static or dynamic. Some parts emphasize the level of mitigation and export (i.e., scenario endpoints), while others refer to the speed of transition. Since the core of the modeling approach appears to be static (optimization for 2030), such temporal references and discussions should be more carefully explained.

AR: Thank you for this helpful comment. We agree that the core of the modeling approach is fundamentally static, based on optimizations for the year 2030, and does not model transition pathways. To avoid potential confusion and clarify our interpretative approach, we have revised the manuscript throughout to ensure a consistent and transparent framing. Specifically, we now explain that the variation of the scenarios is used to explore different sequences of policy ambition, that is, whether the hydrogen export or domestic decarbonization is prioritized earlier while keeping the model itself fixed to a single year. We have implemented the following changes:

In the Introduction, we clarify the scope in lines 76 f.:

> *While our model is static, the scenario design allows exploration of different sequences in export and mitigation ambition.*

In the Results, we explicitly introduce this framing at the beginning of the broader scenario analysis in lines 237 ff.:

> *Based on the effects of temporal hydrogen regulation in the scenario with 120 TWh/a exports and 0% CO_2 mitigation presented in the previous section, we now broaden the analysis to the full scenario space of mitigation (0–100%) and export levels (0–120 TWh/a). Although the model is static and optimized for 2030, we interpret the scenario variation as reflecting different sequences of policy ambition – that is, whether hydrogen exports or domestic decarbonization are prioritized earlier on the pathway toward shared long-term goals. For instance, a scenario with high exports and low CO_2 mitigation can reflect a pathway where export infrastructure is deployed early, with decarbonization following later and vice versa. To explore this sequencing, we group scenarios into three illustrative transition strategies: (i) Quick exports and slow CO_2 mitigation, (ii) Balanced exports and CO_2 mitigation, and (iii) Slow exports and quick CO_2 mitigation as further elaborated in Supplementary Fig. 3a. These groupings help illustrate the impacts of temporal hydrogen regulation on, e.g., domestic electricity cost, even within a static modeling framework.*

In the Discussion section, we remind the reader of this framing in lines 279 f.:

> *As outlined in Results, we use static scenarios to reflect different sequences of policy ambition toward shared long-term goals.*

In the caption of Figure 7, we avoid the term "speeds of transformation" and instead write:

> *[...] three stylized transition strategies [...] reflecting different sequences of export and mitigation ambition [...]*

Additionally, we revised the description of Figure 7 to align with the clarified terminology and framing. The updated sentence now reads in lines 246 f.:

> *Figure 7 shows the relative change of electricity and hydrogen cost of all mitigation-export scenarios (grouped by transition strategy) in dependence of temporal matching.*

In the caption of Supplementary Figure 3a, we now write:

> *These groupings reflect different sequences of policy ambition evaluated at a fixed modeling year.*

Finally, the Methods section section already included a clarification prior to this revision that the model is fixed to 2030 and does not represent dynamic pathways. We retain this paragraph, which states in lines 402 ff.:

> *By fixing the modelling year to 2030 instead of modelling transformation pathways, we can isolate the effects of 0–100% mitigation (to today) and 0–120 TWh hydrogen export and contextualize our results in a comparable setting. However, this fixed-year approach has limitations. Modeling actual transition pathways (instead of a fixed year) accounts for evolving factors over time, such as technology costs and regulatory shifts.*

We hope these changes fully clarify the distinction between our static model and the qualitative interpretation of policy sequencing across scenarios.

Reviewer #4

RC: I co-reviewed this manuscript with one of the reviewers who provided the listed reports. This is part of the Nature Communications initiative to facilitate training in peer review and to provide appropriate recognition for Early Career Researchers who co-review manuscripts.

AR: We appreciate the efforts of both reviewers in providing detailed and constructive feedback. We also value the Nature Communications initiative supporting Early Career Researchers in peer review, which we believe strengthens the scientific community.

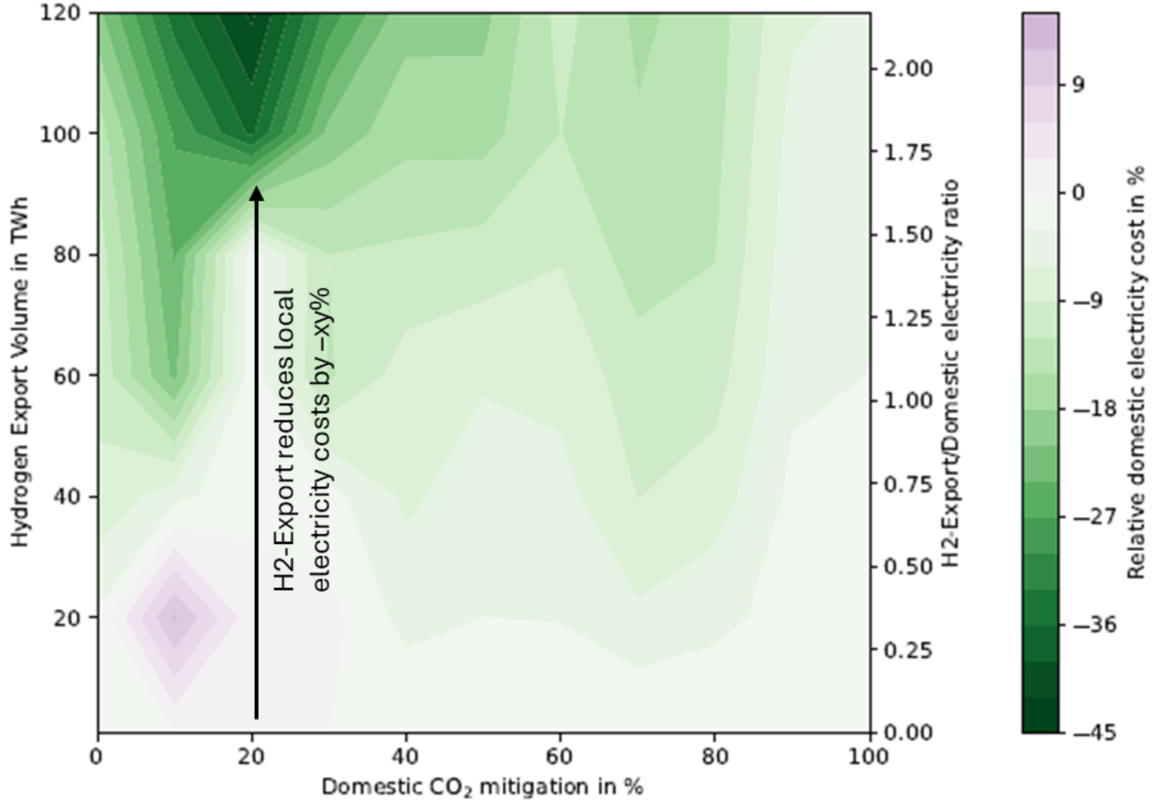

(a) Relative cost of electricity for domestic customers (normalized to 1 TWh/a hydrogen export)

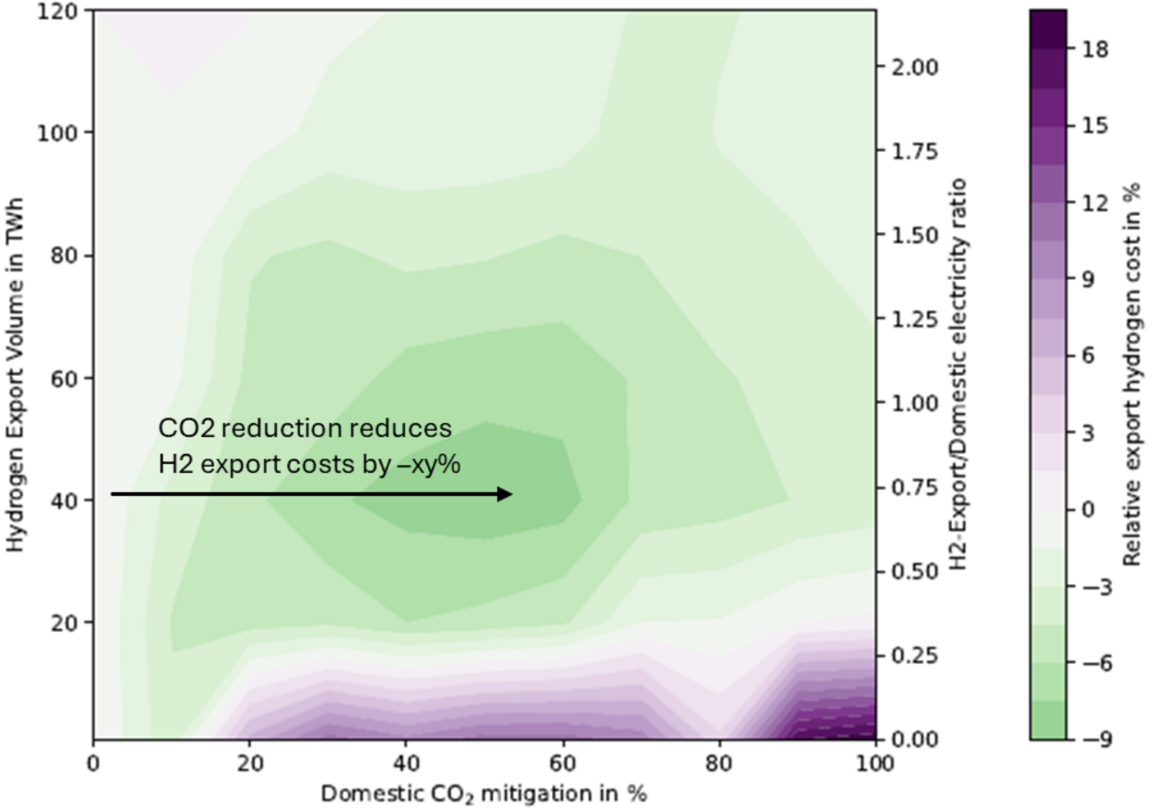

(b) Relative cost of hydrogen for exporters (normalized to 0% domestic CO_2 mitigation)
